# Multifunctional Graphdiyne Enables Efficient Perovskite Solar Cells via Anti-Solvent Additive Engineering

**DOI:** 10.1007/s40820-024-01630-y

**Published:** 2025-01-28

**Authors:** Cong Shao, Jingyi He, Jiaxin Ma, Yirong Wang, Guosheng Niu, Pengfei Zhang, Kaiyi Yang, Yao Zhao, Fuyi Wang, Yongjun Li, Jizheng Wang

**Affiliations:** 1 CAS Key Laboratory of Organic Solids, Institute of Chemistry, Beijing National Laboratory for Molecular Sciences, Chinese Academy of Sciences, Beijing, 100049 People’s Republic of China; 2 University of Chinese Academy of Sciences, Beijing, 100049 People’s Republic of China; 3 CAS Key Laboratory of Engineering Plastics, Institute of Chemistry, Chinese Academy of Sciences, Beijing, 100049 People’s Republic of China; 4 CAS Key Laboratory of Photochemistry, Institute of Chemistry, Chinese Academy of Sciences, Beijing, 100049 People’s Republic of China; 5 National Centre for Mass Spectrometry in Beijing, CAS Key Laboratory of Analytical Chemistry for Living Biosystems, Beijing National Laboratory for Molecular Sciences, Chinese Academy of Sciences, Beijing, 100049 People’s Republic of China

**Keywords:** Perovskite solar cells, Graphdiyne, Anti-solvent additive engineering, Crystallization, Defect passivation

## Abstract

Finding ways to produce dense and smooth perovskite films with negligible defects is vital for achieving high-efficiency perovskite solar cells (PSCs). Herein, we aim to enhance the quality of the perovskite films through the utilization of a multifunctional additive in the perovskite anti-solvent, a strategy referred to as anti-solvent additive engineering. Specifically, we introduce ortho-substituted-4′-(4,4″-di-tert-butyl-1,1′:3′,1″-terphenyl)-graphdiyne (o-TB-GDY) as an AAE additive, characterized by its *sp*/*sp*^2^-cohybridized and highly *π*-conjugated structure, into the anti-solvent. o-TB-GDY not only significantly passivates undercoordinated lead defects (through potent coordination originating from specific high *π*–electron conjugation), but also serves as nucleation seeds to effectively enhance the nucleation and growth of perovskite crystals. This markedly reduces defects and non-radiative recombination, thereby increasing the power conversion efficiency (PCE) to 25.62% (certified as 25.01%). Meanwhile, the PSCs exhibit largely enhanced stability, maintaining 92.6% of their initial PCEs after 500 h continuous 1-sun illumination at ~ 23 °C in a nitrogen-filled glove box.

**Supplementary Information:**

The online version contains supplementary material available at 10.1007/s40820-024-01630-y.

## Highlights

- The use of novel nanographdiyne (o-TB-GDY) via anti-solvent additive engineering significantly enhances the nucleation and growth of perovskite crystals, leading to improved film quality, reduced film defects and suppressed non-radiative recombination.
- o-TB-GDY primarily remains on the surface of the perovskite films after crystallization, where it strongly interacts with the undercoordinated Pb defects for effective passivation.
- The optimized perovskite solar cells achieve a champion power conversion efficiency of 25.62% (certified as 25.01%) with good stability.

**Supplementary Information:**

The online version contains supplementary material available at 10.1007/s40820-024-01630-y.

## Graphical abstract

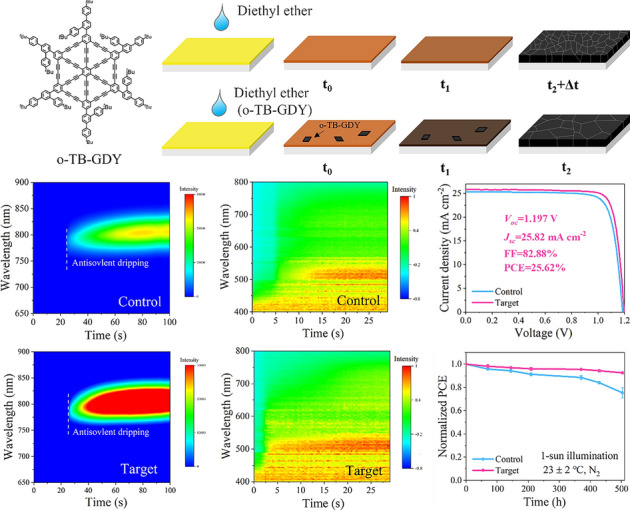

## Introduction

FAPbI_3_-based perovskite solar cells (PSCs) have drawn tremendous attention during the past decade and reached certified efficiencies of 26.7% in single-cell devices [1]. Such remarkable progress mainly comes from improvements in perovskite film quality [2–16]. Among the various methods capable of producing high-quality perovskite films, the one-step deposition process is especially appealing for industrial production due to its simplicity and cost-effectiveness. Initially, the conventional one-step deposition process had several critical drawbacks, including poor film coverage on the substrate due to challenges in controlling perovskite nucleation and crystallization [17, 18]. The anti-solvent approach was subsequently developed, wherein anti-solvents like toluene and chlorobenzene were dropped on the substrate during spin-coating of perovskite precursors [19, 20]. This process extracts residual solvents from the perovskite film almost instantaneously, resulting in uniform and smooth films with minimal pinholes. However, such solution-processed polycrystalline perovskite films still present abundant defects, such as undercoordinated Pb^2+^ and halide (I^−^) ions, leading to serious non-radiative recombination and uncompetitive device performance [21–23]. To address this issue, various effective additives have been incorporated into the perovskite precursor solution to control crystallization [24, 25]. Nevertheless, it is still a significant challenge to simultaneously achieve surface passivation and improved perovskite grain growth. Thus, a promising approach known as anti-solvent additive engineering (AAE) has been introduced. Specifically, functional additives, such as 2-hydroxyethyl faacrylate (HEA), 2-amidinopyrimidine hydrochloride (APC) and lead(II) 2-ethylhexanoate (LDE), have been added into the anti-solvent [26–28]. Although AAE has shown promise, there are currently few reports on AAE-based PSCs, and only a limited number of additives have been explored.

Graphdiyne (GDY), as a branch of the 2D carbon family, possesses *sp*/*sp*^2^-cohybridized and highly π-conjugated structure [29]. Owing to this unique structure, GDY exhibits high carrier mobility, a tunable band gap and strong light absorption, and has been widely applied in optoelectronics [30–34]. Nonetheless, in-depth investigations into GDY-regulated perovskite nucleation and crystallization remain extremely rare, crucial for fabricating high-quality perovskite films and enhancing device performance and stability. Moreover, despite early studies demonstrating the potential of GDY in PSC applications, effectively utilizing GDY to exceed the 25% efficiency threshold alongside enhanced stability remains a significant challenge.

In this study, we introduced a novel nanographdiyne (o-TB-GDY) as an additive into the anti-solvent to simultaneously enhance crystallization and passivate surface defects of the perovskite film. o-TB-GDY possesses a wheel-shaped structure comprising six dihydrobenzo annulenes, facilitating π–electron conjugation along its giant π core. The high π-conjugation enables robust coordination with uncoordinated Pb defects at the triangular poles of o-TB-GDY. Furthermore, the AAE technique facilitates the deposition of o-TB-GDY at varying depths within the perovskite film, forming a gradient distribution near the interface. The gradient-distributed o-TB-GDY serves as nucleation seeds, significantly enhancing the nucleation and growth of perovskites. Consequently, the o-TB-GDY-treated PSCs with an n–i–p structure exhibit a champion PCE of 25.62% (certified as 25.01%).

## Experimental Section

### Materials

SnO_2_ colloid precursor (tin(IV) oxide, 15% in H_2_O), N,N-dimethylformamide (DMF, 99.8%) and dimethyl sulfoxide (DMSO, 99.8%) were purchased from Alfa Aesar. Chlorobenzene (99.8%) and isopropanol (99.5%) were purchased from Sigma-Aldrich. Acetonitrile (99.9%) was purchased from Acros. PbI_2_ (99.999%) was purchased from TCI. CsI (99.999%) was purchased from Sigma-Aldrich. FAI (99.9%) and MACl (99.9%) were purchased from Advanced Election Technology Co., Ltd. MAPbBr_3_ (99%), MeO-PEAI (99%), spiro-OMeTAD (99.9%), 4-tert-butylpyridine (96%) and LiTFSI (99%) were purchased from Xi’an Yuri Solar Co., Ltd.

### Preparation of o-TB-GDY Solution

The o-TB-GDY synthesis method was described in previous report [35], which was realized through the sixfold intramolecular Eglinton coupling in the hexabutadiyne precursors obtained by the sixfold Cadiot–Chodkiewicz cross-coupling of hexaethynylbenzene. o-TB-GDY solution was made by dispersing o-TB-GDY (1 mg) in chlorobenzene (CB) (1 mL) and then ultrasonically treated at 25 °C for 24 h. Then, o-TB-GDY solution was diluted in CB, resulting in designed concentrations (0.2, 0.6 and 1 mg mL^−1^). Afterward, the solutions were diluted in diethyl ether (DE) to obtain wanted concentrations (0.01, 0.03 and 0.05 mg mL^−1^), the ratio of DE:CB in all solutions is 95:5 v:v%.

### Preparation of Anti-Solvent

Control: Diethyl ether was used as anti-solvent; reference 1: 0.03 mg mL^−1^ o-TB-GDY was spin-coated on the surface of perovskite; reference 2: DE:CB (95:5 v:v%) was used as anti-solvent; and target: 0.03 mg mL^−1^ o-TB-GDY in DE:CB (95:5 v:v%) was used as anti-solvent. The corresponding perovskite films are named as the control, reference 1, reference 2 and target films.

### Preparation of Perovskite Precursor Solution

#### ***Preparation of Cs***_***0.05***_***FA***_***0.95***_***PbI***_***3***_

The Cs_0.05_FA_0.95_PbI_3_ precursor solution contained 1.4 M FAI, 0.07 M CsI, 1.58 M PbI_2_ and 0.49 M MACl in a mixed solvent of DMF and DMSO (volume ratio: 8:1). The perovskite solution (60 μL) was spin-coated onto the SnO_2_-coated indium tin oxide (ITO) substrate at 1,000 and 5,000 rpm for 10 and 30 s, respectively. Different anti-solvent (400 µL) was added dropwise onto the perovskite film at 15–20 s from the start of the spin-coating process. The as-prepared films were annealed at 120 °C for 1 h under an ambient atmosphere with ~ 20% RH.

#### ***Preparation of (FAPbI***_***3***_***)***_***0.99***_***(MAPbBr***_***3***_***)***_***0.01***_

The (FAPbI_3_)_0.99_(MAPbBr_3_)_0.01_ precursor solution contained 1.4 M FAI, 1.53 M PbI_2_ (9 mol% excess), 0.014 M MAPbBr_3_, 0.49 M MACl in a mixed solvent of DMF and DMSO (volume ratio: 8:1). The perovskite solution (60 μL) was spin-coated onto the SnO_2_-coated ITO substrate at 1,000 and 5,000 rpm for 10 and 30 s, respectively. Different anti-solvent (400 µL) was added dropwise onto the perovskite film at 15–20 s from the start of the spin-coating process. The as-prepared films were annealed at 120 °C for 1 h under an ambient atmosphere with ~ 20% RH.

### Device Fabrication

ITO glass was cleaned sequentially with detergent, deionized water, acetone and isopropanol. Before use, the ITO glass was dried with a nitrogen gun and treated with oxygen plasma for 10 min to improve its wettability. Then the SnO_2_ (1:2 v:v% diluted by water) was spin-coated on the ITO glass at 4,000 rpm for 30 s and annealed in ambient air at 170 °C for 30 min. The perovskite was deposited via a one-step spin-coating method as mentioned above. Then MeO-PEAI solution (3 mg mL^−1^ in IPA) was spin-coated on the perovskite film for passivation at 4,000 rpm for 30 s. Then the perovskite film was annealed at 100 °C for 5 min. The spiro-OMeTAD solution (50 μL), which consisted 72.3 mg of spiro-OMeTAD, 29 μL of tBP, 35 μL of LiTFSI solution (260 mg mL^−1^ in acetonitrile) and 1 mL CB, was spin-coated onto the perovskite layer at 4,000 rpm for 30 s. Finally, 80 nm Au electrode was deposited by thermal evaporation.

### Density Functional Theory Calculations

First-principles calculations were performed in the framework of the density functional theory (DFT) using the program package DMol^3^, the Perdew–Burke–Ernzerhof (PBE) within the generalized gradient approximation (GGA) was used in the module for structural optimization, and double numerical atomic orbits enhanced by d-polarization functions (DNP basis sets) were used for supercell optimization. To calculate the total energy of nanoGDY, it was placed inside a 25.4 Å × 25.4 Å × 27.7 Å lattice while allowing their atomic positions to vary. The simulations stopped when the total energies converged to 2.0 × 10^–5^ Ha, forces on each unconstrained atom were smaller than 4 × 10^–3^ Ha Å^−1^, and displacements were < 0.005 Å.

To calculate the adsorption energies of nanoGDY on the FAI-terminated FAPbI_3_ (001) surface, we modeled the first five layers of this surface. To obtain the most stable structure, structural optimization was performed by fixing the bottom three layers and setting the thickness to 15 Å vacuum. Adsorption energies were calculated by the following equation:

1 $$\Delta {E}_{ad}=\Delta {E}_{{\text{FAPbI}}_{3}/\text{nanoGDY}-\text{Me}}-\Delta {E}_{{\text{FAPbI}}_{3}\_001}-\Delta {E}_{\text{nanoGDY}-\text{Me}}$$

where $\Delta {E}_{{\text{FAPbI}}_{3}/\text{nanoGDY}-\text{Me}}$, $\Delta {E}_{{\text{FAPbI}}_{3}\_001}$ and $\Delta {E}_{\text{nanoGDY}-\text{Me}}$ represent the total energies of the adsorption system, FAI-terminated FAPbI_3_ (001) surface and nanoGDY–Me, respectively.

### Measurements

The current density–voltage (*J–V*) characteristics of the devices were measured using a Keithley 2420 under AM 1.5 sunlight at an irradiance of 100 mW cm^−2^ provided by a solar simulator (Newport, Oriel Sol3A Class AAA, 94043A). Light intensity was calibrated using a monocrystalline silicon reference cell with a KG5 window (Newport, Oriel 91,150). The *J–V* curves were obtained in the range from 1.2 to 0.0 V with a scan step of 20 mV from both reverse and forward scan directions. The area of the cell is 0.1225 cm^2^, and a mask of 0.09881 cm^2^ (certificated by NIM, China. The certificate No.: CDjc2023-08390) was used to determine the effective area of the device before the test. External quantum efficiency (EQE) measurements were recorded by an Enli Technology (Taiwan) EQE system. The top-view and cross-sectional scanning electron microscopy (SEM) images were obtained using Hitachi S-4800 at the accelerating voltage of 5.0 kV. The surface roughness of perovskite film was measured by an atomic force microscopy (AFM) (Nanoscope V, Vecco) in tapping mode under the ambient atmosphere. X-ray diffraction measurements were taken by using a PANalytical Empyrean with a Cu Kα radiation (*λ* = 1.5406 Å). 2D-XRD spectra were measured using a Rigaku SmartLab X-ray diffractometer (XRD) with Cu Kα1 (1.54060 Å) and a HyPix-3000 2D hybrid pixel array detector. All samples for XRD testing were prepared on quartz glass substrates. The steady photoluminescence (PL) spectra and photoluminescence quantum yield (PLQY) were recorded by Horiba FluoroMax + fluorescence spectrometer with an excitation at 490 nm. Time-resolved PL was measured by the FLS980 fluorescence spectrometer with excitation wavelength at 485 nm. PL, PLQY and time-resolved PL (TRPL) samples were prepared on ITO glass substrates. ToF–SIMS samples were analyzed using a ToF–SIMS 5 instrument (IONTOF) with a Bi + primary beam (10 keV and 1 pA) and Cs^+^ sputter beam (3 keV and 5 nA). The UV absorption spectra of perovskite films were measured using a SHIMADZU UV-2600 spectrophotometer. X-ray photoelectron spectroscopy (XPS)/ultraviolet photoelectron spectroscopy (UPS) measurements were obtained using an XPS/UPS system (ESCALAB250XI, Thermo Fisher Scientific). Fourier transform infrared spectroscopy (FTIR) was performed on a HITACHI F-4500IR spectrometer with samples prepared as KBr tablets. Raman spectra were recorded using an NT-MDT NTEGRA Spectra system. EIS and M-S tests were measured with an electrochemical workstation (Modulab XM, USA).

## Results and Discussion

### Interaction Between o-TB-GDY and Perovskite

**Fig. 1:**
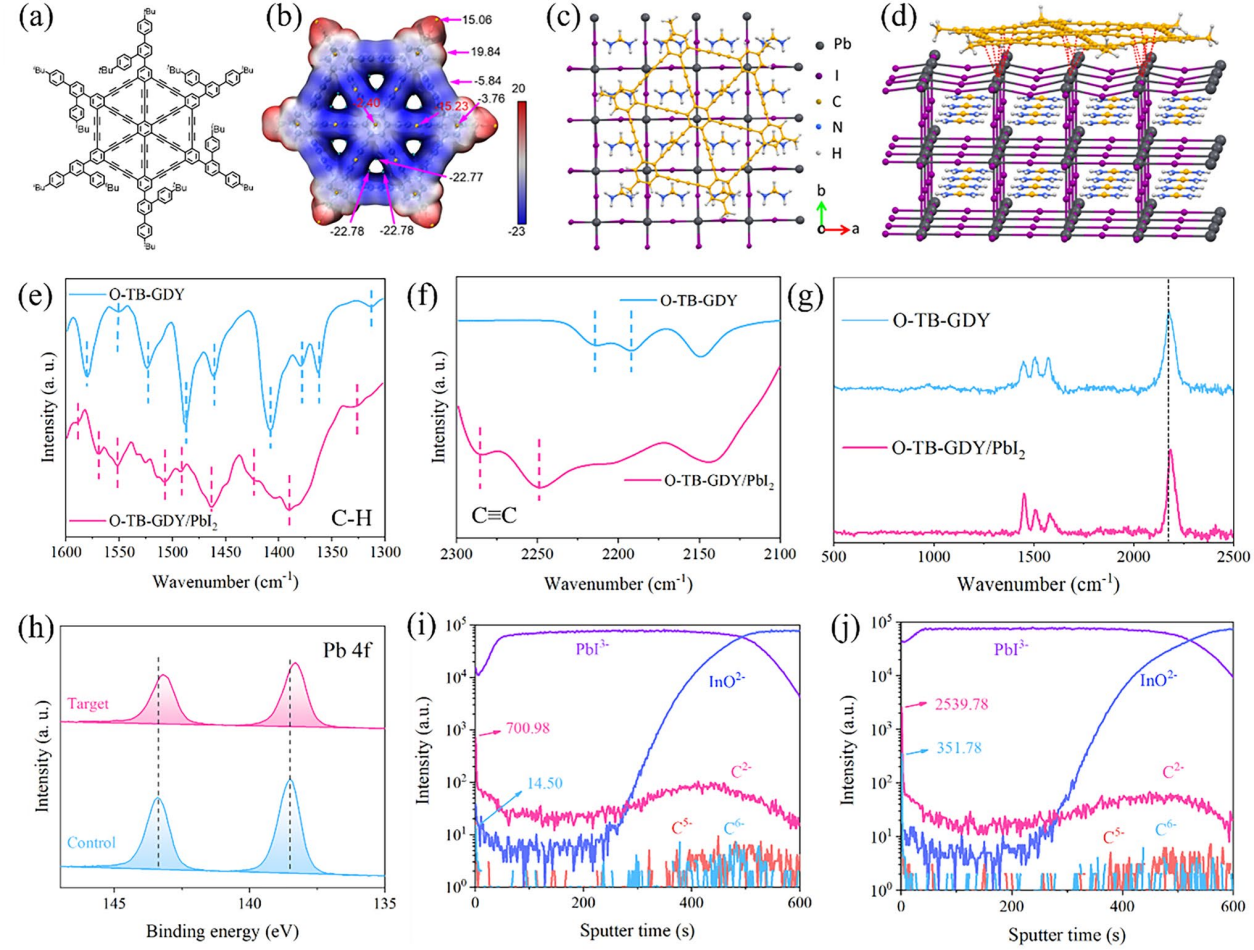
Interaction between o-TB-GDY and perovskite.** a** Chemical structure of o-TB-GDY. **b** Calculated electrostatic potential on van der Waals surface of nanoGDY with methyl groups as outside substitutes (nanoGDY–Me) [35]. **c, d** Top view and side view of the crystal structure model of nanoGDY–Me interaction with FAPbI_3_. **e, f** FTIR spectra of o-TB-GDY and o-TB-GDY/PbI_2_. (The blue dotted lines and the pink dotted lines correspond to each other, illustrating the displacement.). **g** Raman spectra of o-TB-GDY and o-TB-GDY/PbI_2_. **h** Pb 4*f* XPS spectra of the control and target films. **i, j** TOF–SIMS of the control and target films

The structure of o-TB-GDY is given in Figs. 1a and S1. Figure 1b shows the electrostatic potential of o-TB-GDY, where positive ESP regions (red) are exclusively present on the outside of peripheral benzene rings, and the triangle poles exhibit the most negative ESP (blue). This suggests that the molecular poles of o-TB-GDY are capable of forming strong electrostatic interaction with cations [35], *e.g.,* the undercoordinated lead. We calculated the binding energy between nanographdiyne with methyl groups as outside substitutes (nanoGDY–Me) (to simplify calculations) and the FAPbI_3_ surface terminated by lead(II) iodide (PbI_2_), as the PbI_2_-terminated surface can be easily formed during the evaporation of the solvents from the film. DFT calculations show that nanoGDY–Me is strongly bonded to the PbI_2_-terminated surface with a binding energy of 4.08 eV (Fig. 1c, d and Table S1). Thus, nanoGDY–Me is predicted to bind and stabilize perovskite grain boundaries. To verify this hypothesis, we conducted FTIR to investigate the interaction between o-TB-GDY and PbI_2_, as depicted in Fig. 1e, f. The signals around 1300–1600 cm^–1^ correspond to the ring stretch of the central benzene and the C–H bending vibration on the peripheral benzene rings, with an overall shift observed after PbI_2_ incorporation. Correspondingly, the C≡C stretching at different locations shifts from 2215/2180 to 2285/2245 cm^−1^, and a similar shift is observed from Raman spectra (Fig. 1g) [36]. Overall, these observations strongly suggest that o-TB-GDY establishes vigorous electrostatic interaction with PbI_2_, which is expected to stabilize Pb^2+^ either on the crystal surface or at the grain boundaries [37–39].

We then performed XPS analysis. Figure 1h shows the Pb 4*f* XPS profiles for the control and target (with o-TB-GDY) perovskite films, and the binding energy of 4*f*_5/2_ (4*f*_7/2_) for divalent Pb^2+^ shifts from 143.38 (138.50) to 143.18 (138.30) eV. This observation strongly suggests that o-TB-GDY exhibits electron-donating properties, which enable it to passivate uncoordinated Pb^2+^ [40–42]. To investigate the distribution of o-TB-GDY in the perovskite film, time-of-flight secondary-ion mass spectrometry (ToF–SIMS) was adopted to observe the depth profiles of the atomic species in the perovskite films with or without o-TB-GDY treatment (Fig. 1i, j). The ToF–SIMS ion image reveals surface intensities of 700.98 (C^2−^) and 14.50 (C^6−^) for the control film. In contrast, the target film exhibits increased surface intensities, reaching 2539.78 (C^2−^) and 351.78 (C^6−^). However, the two signals (C^2−^ and C^6−^) have no significant difference within the bulk of the films, indicating that the AAE technique primarily distributes o-TB-GDY on the perovskite film surface. In addition, to further verify the distribution of o-TB-GDY, we employed high-resolution transmission electron microscopy (HRTEM). The TEM specimen was prepared using cryogenic focused ion beam (cryo-FIB) technology, ensuring minimal alteration to the intrinsic structures of the perovskite layer. As shown in Fig. S2, a substantial amount of o-TB-GDY is observed near the interface, with its content gradually decreasing deeper into the perovskite layer. This gradient distribution suggests that AAE technique can infiltrate a small amount of o-TB-GDY into the perovskite layer, forming the gradient structure observed in Fig. S3, as reported previously [26, 43].

We then dispersed o-TB-GDY in the perovskite precursor and conducted dynamic light scattering (DLS) measurements. As depicted in Fig. S4, the characteristic particle size over 1 μm is observed in the target precursor. We infer that o-TB-GDY could serve as nucleation seeds to induce perovskite crystallization [44–46]. The crystallization process of perovskite can be explained by the classical model of Gibbs free energy. As shown in Fig. S5, the crystallization of perovskite requires two processes: nucleation and growth. The crystallization kinetics can be explained by the Gibbs free energy (ΔG) that is sum of the surface free energy (ΔG_S_) and the bulk free energy (ΔG_V_). The formation of nuclei is strongly dependent on the critical radius (r^*^); when nuclei radii is below the r^*^, the nuclei are dissolved back to the solution, but when nuclei radii is greater than r^*^, the nuclei are thermodynamically stable and would grow spontaneously. Therefore, in the target film, o-TB-GDY is introduced as the seeds of the following perovskite nucleation. The seeds could significantly reduce the interface energy and the perovskite nucleation barrier, enabling nucleation occurs at a lower saturation, thereby accelerating the nucleation process. This rapid nucleation is crucial for forming high-quality perovskite films with extensive coverage according to the LaMer model [47].

### Characterization of the Perovskite Films

**Fig. 2:**
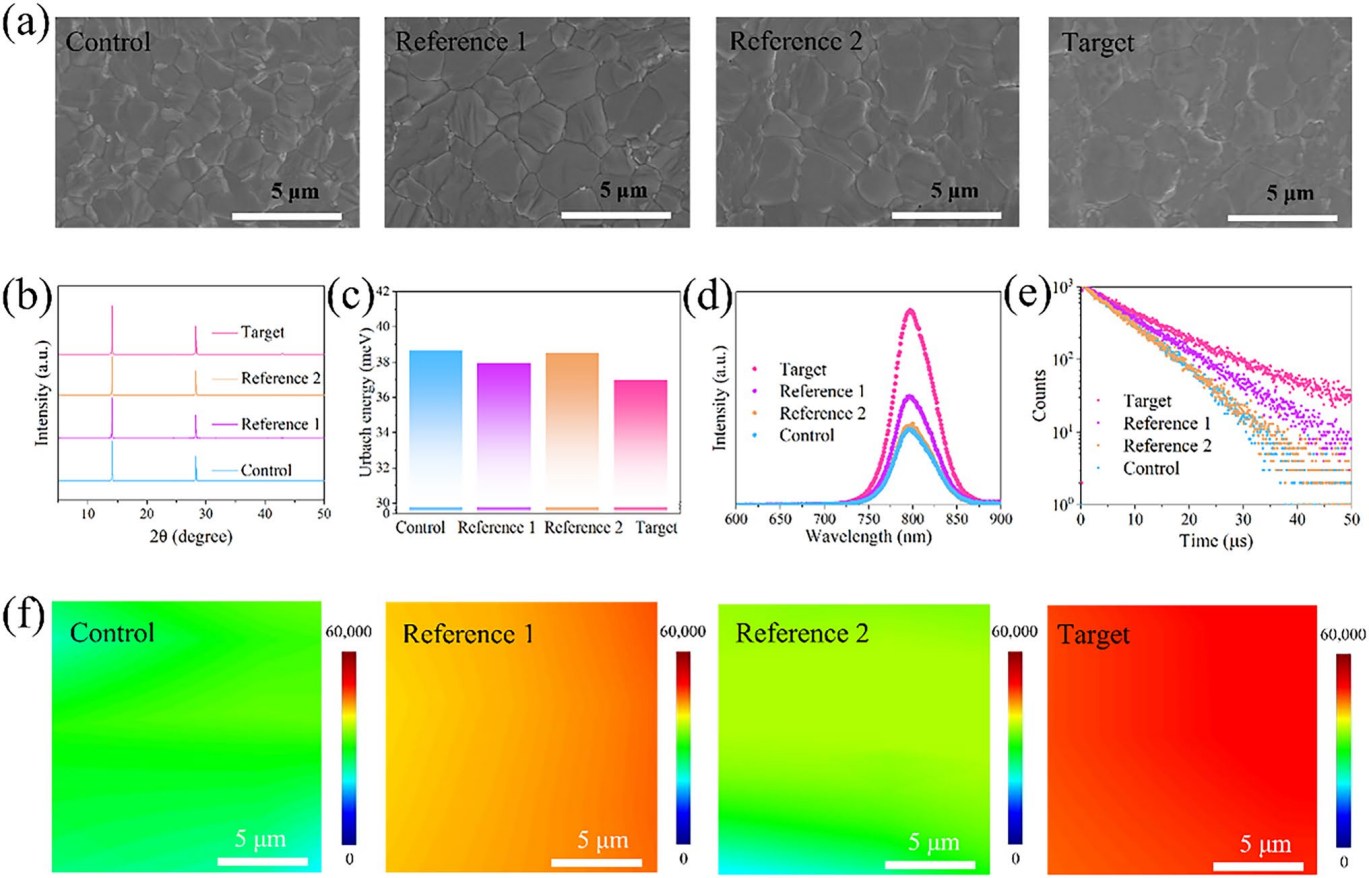
Characterizations of the perovskite films. **a** SEM images. **b** XRD patterns. **c** Urbach energy calculated from UV–Vis absorption spectra. **d** Steady-state PL spectra.** e** Time-resolved PL spectra. **f** PL mapping images

The surface morphologies of the perovskite films naming control, reference 1, reference 1 and target were studied by SEM (Figs. 2a and S6). The four films are differentiated as follows: (1) Control: diethyl ether (DE) was used as anti-solvent. (2) Reference 1: o-TB-GDY was spin-coated on top of the control film. (3) Reference 2: DE:CB (95:5 v:v%) was used as anti-solvent. (4) Target: DE:CB (95:5 v:v%) with o-TB-GDY (0.03 mg mL^−1^) was used as anti-solvent.

We observed that all the four films exhibit uniform surface morphology without pinholes. Comparing reference 2 with the control films, it can be judged that the introduction of a small fraction of CB to DE anti-solvent has negligible effects on perovskite growth (Fig. S6). Notably, the use of o-TB-GDY in the DE:CB anti-solvent markedly enhances crystal growth, and the average grain size increases from 1.60 to 1.71 and 2.01 μm as the o-TB-GDY content is augmented from 0 to 0.01 mg mL^−1^ and then to 0.03 mg mL^−1^ (Fig. S7). However, upon further increasing the o-TB-GDY content to 0.05 mg mL^−1^, the average grain size is slightly reduced to 1.80 μm (Fig. S8). Therefore, we used the film made with an o-TB-GDY content of 0.03 mg mL^−1^ as the target film. These results indicate that the change in grain size is due to o-TB-GDY rather than a small amount of CB in the DE:CB anti-solvent. In addition, cross-sectional SEM images (Fig. S9) reveal that with o-TB-GDY integrated into the anti-solvent, the crystals are monolithic with indistinguishable grain boundaries. The perovskite films were also studied by AFM (Fig. S10), which further demonstrates that adding o-TB-GDY into the DE:CB anti-solvent significantly increases the grain size without affecting the surface morphology and roughness. These results demonstrate that o-TB-GDY effectively facilitates perovskite crystallization and leads to a high-quality perovskite film with enlarged grain size.

Figure 2b shows the XRD patterns of the perovskite films. No peak shifts or additional peaks are observed, indicating that the o-TB-GDY treatment does not change the cubic perovskite structure. Furthermore, the (001) and (002) peaks are stronger in the target film compared to the control film, suggesting enhanced crystallinity [48]. Besides, the target film exhibits a narrow and sharp full width at half maximum (FWHM) for the (001) diffraction peak. The FWHM value of the target film decrease from 0.073 to 0.067, further suggesting that the o-TB-GDY treatment improves the crystallinity of the perovskite films [49]. To further probe the crystal orientations, 2D X-ray diffraction (2D-XRD) measurements were taken. As illustrated in Fig. S11, all the films exhibit sharp Bragg spot of (001)/(002) planes along the out-of-plane(q_z_) direction [50], indicating a well-aligned α-FAPbI_3_ structure. To assess the structural quality of the films, Urbach energies (${E}_{u}$) were calculated from the ultraviolet (UV)–visible absorption spectra using the following equation:

2 $$\alpha ={\alpha }_{0}\text{exp}(h\upnu /{E}_{u})$$

where α represents the absorption coefficient and $h\upnu$ signifies the photon energy. The ${E}_{u}$ values of the four films are 38.67, 37.94, 38.51 and 36.98 meV, respectively (Figs. 2c and S13). The fact that the target film owns the lowest *E*_u_ indicates that it has the highest structural quality [12]. Furthermore, the band gaps of the films are all estimated to be 1.53 eV (Fig. S12).

Steady-state PL and TRPL measurements were taken for the perovskite films. The control and reference 2 films exhibit similar PL intensities, while reference 1 film shows a stronger PL intensity, which is attributed to the surface passivation induced by o-TB-GDY. Notably, the target film displays significantly higher PL intensity than all the other films (Fig. 2d), indicating enhanced film quality. Meanwhile, the carrier lifetime of the target film (*τ* = 11.43 μs) largely surpasses those of the control, reference 1 and reference 2 films (*τ* = 7.73, 9.68, 7.54 μs) (Fig. 2e and Table S2). The high PL intensity and prolonged carrier lifetime of the target film can be attributed to the effective passivation of surface defects and enhanced crystallization facilitated by o-TB-GDY [51]. Further detailed PL and TRPL studies with varying concentrations of o-TB-GDY are provided in Figs. S14, S15 and Table S3.

Additionally, when a 2,2′,7,7′-tetrakis (N,N-di-p-methoxyphenyl-amine)-9,9′-spirobifluorene (spiro-OMeTAD) layer was deposited atop, all the films exhibit significantly reduced carrier lifetimes of ~ 40 ns (Fig. S16 and Table S4), indicating efficient charge extraction from the perovskite to the hole transporting layer (HTL). Consistent results are also obtained for the steady-state PL spectra (Fig. S17) [52]. Furthermore, the hole diffusion length is calculated using a simplified 1D diffusion model:

3 $$\frac{{\text{L}}_{\text{D}}}{\text{L}}=\frac{2}{\pi }\sqrt{\frac{1}{{\uptau }_{\text{D}}/\uptau }-1}$$

where ${L}_{\text{D}}$ represents the diffusion length, $\text{L}$ is the thickness of the perovskite film, ${\tau }_{\text{D}}$ and $\tau$ denote the lifetimes of the perovskite film with and without the HTL, respectively. The estimated diffusion lengths are provided in Fig. S18. The target film exhibits a hole diffusion length of 6935 nm, which is larger than the 5746 nm for the control film. Such long carrier diffusion length (over 6 μm) is comparable to that observed in single-crystal perovskite [53]. Moreover, the target film shows the highest PLQY (Fig. S19 and Table S5) among the films, consistent with its strongest intensity in the PL mapping measurement (Fig. 2f). These results further indicate that the o-TB-GDY treatment significantly suppresses the bulk and surface non-radiative recombination.

XPS spectra (Fig. S20) show that C–C = O peak (288.4 eV) associated with oxygen/moisture is slightly suppressed after the o-TB-GDY treatment, demonstrating that o-TB-GDY could slow down the degradation of the perovskite film [13, 54]. Both the Pb 4*f* and I 3*d* from the target film shift to lower binding energies compared to the control film, which is caused by electrostatic interaction between Pb^2+^ and o-TB-GDY [55, 56]. We then utilized UPS and low-energy inverse photoemission spectroscopy (LEIPS) to investigate the energy band structure of the perovskite films. As shown in Fig. S21, the secondary electron cutoff edge (*E*_cutoff_) shifts from 16.92 to 16.83 eV with o-TB-GDY treatment, resulting in an increase work function from 4.30 to 4.39 eV. Meanwhile, the conduction band (*E*_CB_) shifts from − 3.92 to − 3.81 eV with o-TB-GDY treatment (Fig. S22). The lowered Fermi level and elevated valence band create favorable surface energetics and band bending, enhancing the built-in electric field, blocking electrons from entering the HTL, and hence reducing carrier recombination and promoting hole extraction (Fig. S23) [3, 57]. Additionally, Kelvin probe force mode (KPFM) was used to study the electrochemical surface properties of the films (Fig. S24), with contact potential difference (CPD) measured by detecting the electrostatic force between the conducting AFM tip and film surface. The target film displayed a higher CPD compared to the control film, which can be attributed to the decreased surface electronic trap density [58, 59], consistent with the UPS results. Furthermore, femtosecond transient absorption (fs-TA) spectroscopy was employed to deeper study the excitonic dynamics of the perovskite films (Fig. S25). Compared to the control film, an enhancement in absorption variation (ΔA) is observed in the target film, corroborating the reduction in Shockley–Read–Hall (SRH) recombination due to the passivation of trap states. Time-resolved absorption at the ground-state bleach (GSB) of 783 nm reveals a decrease in the fast decay lifetime (*τ*_1_) and an increase in the slow decay lifetime (*τ*_2_). The reduction of *τ*_1_ suggests effective passivation, while the increase of *τ*_2_ implies seamless carrier diffusion within the target film [7] (Table S6).

### Mechanism of Perovskite Nucleation and Crystallization

**Fig. 3:**
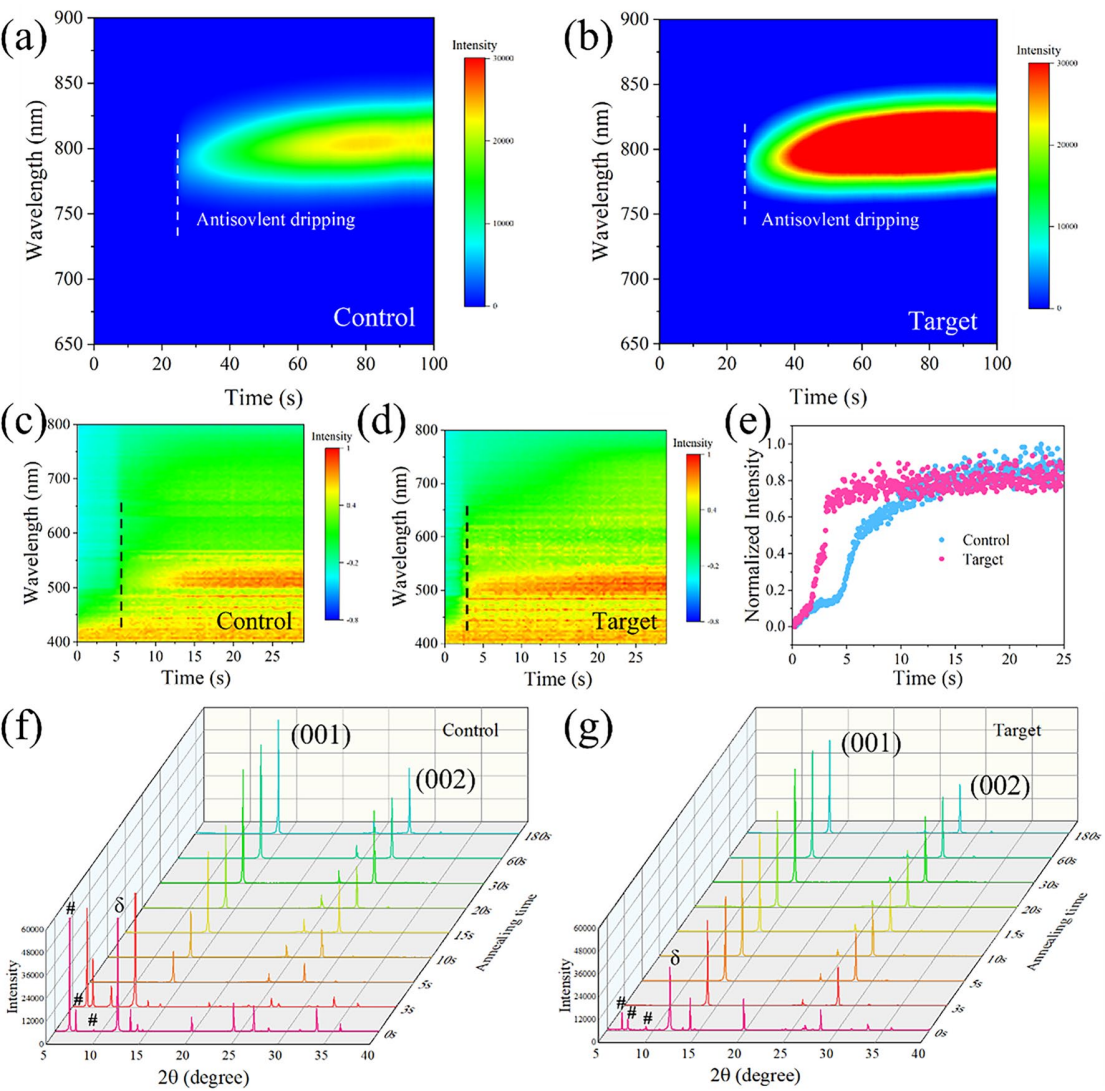
In situ investigation of perovskite nucleation and crystallization process. **a, b** In situ PL spectra of the control and target films during the spin-coating process. **c, d** In situ UV–Vis absorption spectra of the control and target films during the annealing process. **e** UV–Vis absorption intensity at the wavelength of 500 nm as a function of annealing time. **f, g** In situ XRD measurement of the control and target films for different annealing times (0, 3, 5, 10, 15, 20, 30, 60 and 180 s); “#” denotes the peaks of intermediate phase and “σ” denotes the peak of σ-FAPbI_3_

We took in situ PL measurements to monitor perovskite nucleation throughout the spin-coating process, as depicted in Fig. 3a, b. The target film exhibits a sharp increase in PL intensity at approximately 800 nm, indicating rapid nucleation of the perovskite phase on the film surface after anti-solvent dripping [58]. In contrast, the control film maintains consistently weak PL intensity throughout the entire spin-coating process, suggesting a slower nucleation rate. These results indicate that o-TB-GDY can facilitate the nucleation process at nucleation sites [46]. We then tested the in situ UV–Vis absorption spectra to monitor perovskite crystallization during thermal annealing (Fig. 3c, d). The as-prepared intermediate film exhibits only UV absorption at wavelengths shorter than 450 nm which is attributed to the solvent complex [60]. During the annealing process, absorption at wavelengths longer than 450 nm gradually increases, corresponding to the transition from the intermediate phase to α-FAPbI_3_. To quantify the difference between the two films, we further analyze the in situ absorption color mapping by extracting the absorption intensity at 500 nm. We then plotted the absorption intensity as a function of annealing time, as shown in Fig. 3e. The target film demonstrates a faster crystallization process compared to the control film, with film absorption reaching its peak 7 s earlier. From the SEM images of the perovskite films at various annealing time (Fig. S26), the target intermediate film shows large perovskite crystal grains with less pinholes, while the control intermediate film exhibits small grains with obvious pinholes. The different morphology of the two intermediate films can be attributed to the faster crystallization process induced by o-TB-GDY treatment.

Besides, in situ XRD measurements were taken to investigate the perovskite phase transition, as shown in Fig. 3f, g. The XRD patterns were collected at different annealing times (0, 3, 5, 10, 15, 20, 30, 60 and 180 s). Before thermal annealing, two intermediate films (0 s) both exhibit three main peaks (denotes by **#** in the figures) at 6.63°, 7.27° and 9.24°, corresponding to the MA_2_Pb_3_I_8_·2DMSO intermediate phase (Fig. 3f). Compared with target film, the control film exhibits much stronger diffraction peaks of non-perovskite phase (δ-FAPbI_3_) and PbI_2_ (001) at 11.8° and 13.2°, respectively. This indicates that o-TB-GDY effectively suppress the formation of δ-FAPbI_3_ in the intermediate stage. After thermal annealing, for the target film, the majority of the intermediate phase and δ-FAPbI_3_ phase are transformed into the α-phase at the first 3 s, while the control film still showed a strong intermediate phase, indicating that o-TB-GDY accelerates the phase transition of perovskite. As the annealing process continues (5 to 180 s), most of remaining solvent evaporates during the first 5 s, and the intermediate phase almost completely transforms into the perovskite phase. Compared to the control film, the target film also exhibits a much faster crystallization and phase transition processes.

We also performed in situ grazing incidence wide-angle X-ray scattering (GIWAXS) to investigate the processes of crystallization and phase transition during perovskite formation. As shown in Fig. S27, signals at q vector of around 0.82 and 1.0 Å^−1^ are assigned for the δ-FAPbI_3_ and α-FAPbI_3_ phase, respectively. From the GIWAXS results, we found that the o-TB-GDY accelerates both crystallization and phase transition processes, which is consistent with the in situ XRD measurements.

These results demonstrate that o-TB-GDY modulates the crystallization kinetics by acting as nucleation seeds, thereby enhancing film quality. A corresponding schematic illustration of crystallization process is shown in Fig. S28.

### Device Performance and Analysis

**Fig. 4:**
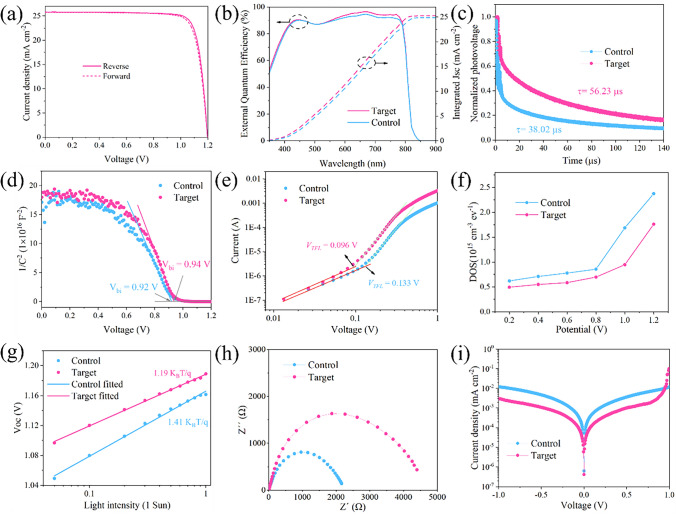
Device characteristics.** a** *J*–*V* curves of the champion target PSC in the reverse and forward modes under AM 1.5G illumination. **b** EQE curve of the control and target PSCs. **c** TPV decay curves of the control and targets PSCs. **d** Mott–Schottky plots for the control and target PSCs. **e** Space-charge-limited current analysis for the control and target hole-only devices. **f** The DOS measurement was assisted by impedance spectroscopy of the control and target PSCs. **g** The relationship between the measured ${V}_{oc}$ and light intensity for the control and target PSCs. **h** Nyquist plots for the control and target PSCs. **i** Dark $J-V$ curves of the control and target PSCs

We examined the impact of o-TB-GDY on the PSCs with a typical n–i–p structure: glass/ ITO/SnO_2_/Cs_0.05_FA_0.95_PbI_3_/Spiro-OMeTAD/Au. The concentration of o-TB-GDY and dosage of anti-solvent were optimized to 0.03 mg mL ^−1^ and 400 μL, respectively, to obtain the best performance (Figs. S29 and S30). The *J*–*V* characteristics of the best-performing target PSC under reverse and forward bias sweeps are illustrated in Fig. 4a. The current density (${J}_{sc}$), open-circuit voltage (${V}_{oc}$), fill factor (FF) and PCE extracted from the *J*–*V* curve in reverse mode are 25.82 mA cm^−2^, 1.197 V, 82.88% and 25.62%, respectively. The corresponding values under forward scan are 25.76 mA cm^−2^, 1.198 V, 81.74% and 25.22%, respectively. The PSCs were sent to Institute of Electrical Engineering Chinese Academy of Sciences for certification, and the certified efficiency is 25.01% (Fig. S31). It is important to highlight that we have fabricated the highest performing n–i–p PSCs to date using GDY materials (Fig. S32, Table S11).

The photovoltaic performances of the control and target PSCs are shown in Fig. S33 and Table S7. Both improvements in ${V}_{oc}$ and FF can be ascribed to the suppressed non-radiative recombination and enhanced charge extraction. The target PSC shows stable-state power output (SPO) over 600 s, with a PCE of 25.37%, confirming both efficiency reliability and operational stability (Fig. S34). Besides, the effect of using o-TB-GDY by perovskite precursor additive engineering was further investigated (for detailed explanation, see in Fig. S35). Figure 4b shows the EQE for the control and target PSCs. The current densities obtained by integrating the EQE over the wavelength of the control and target PSCs are 24.83 and 25.22 mA cm^−2^, respectively, which is in good agreement with the ${J}_{sc}$ in the *J*–*V* curve (Fig. 4b). Analysis of the EQE spectrum suggests a perovskite band gap of ~ 1.53 eV (Fig. S36), which is consistent with that deduced from the optical absorption measurement. We also fabricated 1-cm^2^ PSC, which displays a high PCE of 23.31% (Fig. S37). In addition, we validated the effectiveness of o-TB-GDY treatment with various anti-solvents, including ethyl acetate (EA), anisole (Ani) and chlorobenzene (CB). The performances of PSCs treated with these anti-solvents are shown in Fig. S38, where the PSCs with o-TB-GDY treatment show significant enhanced performance. We also verify the o-TB-GDY treatment in other different perovskite compositions. Both the (FAPbI_3_)_0.99_(MAPbBr_3_)_0.01_ and Cs_0.05_MA_0.05_FA_0.9_PbI_3_ PSCs show significant enhancements in photovoltaic parameters upon o-TB-GDY treatment (Figs. S39 and S40). These findings demonstrate the universality and robustness of this treatment across diverse anti-solvent systems and perovskite compositions.

We then conducted an in-depth analysis of photovoltage loss to understand the improvements in ${V}_{OC}$ of the PSCs. The transient photovoltage (TPV) decay curves (Fig. 4c) show that the target PSC exhibits a slower photovoltage decay in contrast to the control PSC, indicating improved carrier transport and reduced carrier recombination, which further contributing to ${V}_{oc}$ enhancement. Furthermore, the increase in ${V}_{OC}$ is supported by the capacitance–voltage (C–V) plot through Mott–Schottky analysis, following the equation:

4 $$\frac{1}{{C}^{2}}=\frac{2\left({V}_{bi}-V\right)}{{A}^{2}e\varepsilon {\varepsilon }_{0}{N}_{A}}$$

where $A$ is the device area, $\varepsilon$ is the relative permittivity, ${\varepsilon }_{0}$ is the vacuum permittivity and ${N}_{A}$ is the carrier concentration. As shown in Fig. 4d, the target PSC has a larger built-in potential (${V}_{bi}$) compared to the control PSC (0.94 vs. 0.92 V). Larger ${V}_{bi}$ provides enhanced driving force for photogenerated carrier separation and transport, thereby contributing to the enhancement of ${V}_{oc}$ [61]. We also evaluated trap densities of the control and target films based on their hole-only devices (ITO/PEDOT:PSS/perovskite/Spiro-OMeTAD/Au) using the space-charge-limited current (SCLC) method [62, 63] (Figs. 4e and S41). The result show that the target device has a lower trap density (1.39 × 10^15^ vs. 1.92 × 10^15^ cm^−3^, Table S8). The reduced trap density in the target device extends carrier lifetimes and minimizes non-radiative recombination losses, thereby enhancing ${V}_{oc}$. We also measured the density of states (DOS) of the perovskite films to further verify the reduced trap density by o-TB-GDY treatment. As shown in Fig. 4f, the target PSC exhibits a relatively lower DOS, indicating markedly decreased trap states in the target film [64], contributing to ${V}_{oc}$ enhancement.

To gain insights into the FF loss, we studied the dependence of ${V}_{oc}$ on light intensity of the PSCs [65, 66]. As shown in Fig. 4g, the target PSC exhibits higher ${V}_{oc}$ values across all light intensities and a lower slope efficiency, approaching that of an ideal diode, indicating suppressed non-radiative recombination and FF enhancement. Electrochemical impedance spectroscopy (EIS) was utilized to assess interfacial charge transfer and recombination. In the Nyquist plot, the high-frequency semicircle represents charge transfer resistance (${R}_{ct}$), while the low-frequency semicircle corresponds to charge recombination resistance (${R}_{\text{rec}}$). The target PSC exhibits an obvious decrease in ${R}_{ct}$ and an increase in ${R}_{\text{rec}}$ (Figs. 4h, S42 and Table S9), revealing enhanced interfacial charge transfer and effective suppression of charge recombination, contributing to minimized FF loss. Additionally, we evaluated the dark current density–voltage (*J*–*V*) characteristics of the PSCs (Fig. 4i). The leakage current and shunt resistance are quantitatively analyzed using the equation:

5 $$J=\frac{V}{{R}_{\text{sh}}}+{J}_{\text{r}}\left({e}^{\frac{eV}{{m}_{r}{k}_{B}T}}-1\right)+{J}_{\text{d}}\left({e}^{\frac{eV}{{m}_{d}{k}_{B}T}}-1\right)$$

where ${R}_{\text{sh}}$, ${J}_{\text{r}}$, ${J}_{\text{d}}$, ${m}_{r}$ and ${m}_{d}$ are the shunt resistance, recombination current density, diffusion current density and constants, respectively [67]. The increased ${R}_{\text{sh}}$ and reduced ${J}_{\text{r}}$ (Table S10) in the target PSC are consistent with the observed improvement in FF.

**Fig. 5:**
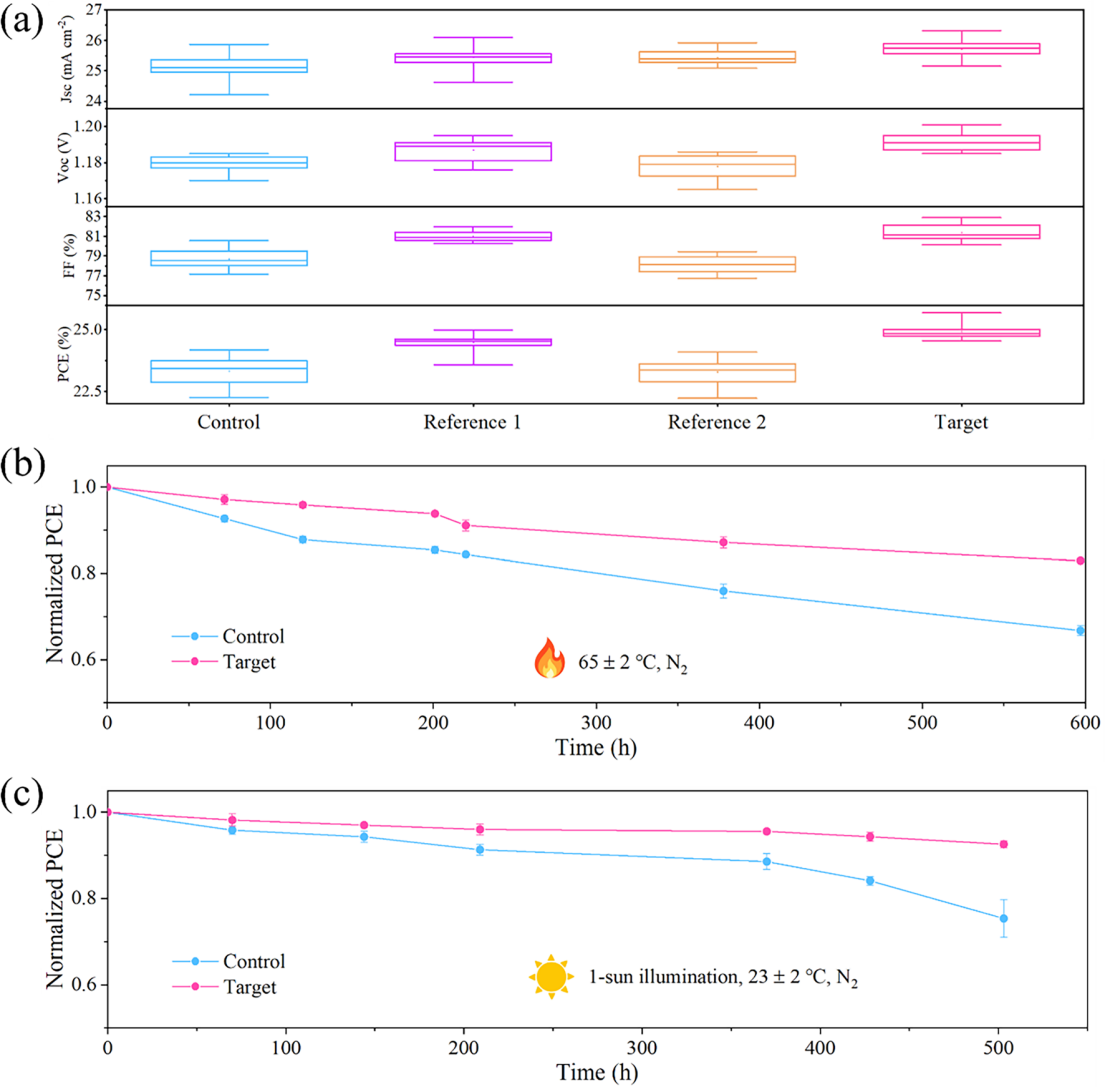
Performance and stability of the PSCs.** a** Statistical distribution of photovoltaic parameters for 15 PSCs (control, reference 1, reference 2 and target). **b** Thermal aging of unencapsulated PSCs at 65 ± 2 °C in a nitrogen atmosphere (ISOS-T-1). **c** Stability of unencapsulated PSCs under 1-sun illumination at 23 ± 2 °C in a nitrogen atmosphere (ISOS-L-1)

Figure 5a displays statistical distribution of ${J}_{sc}$, ${V}_{oc}$, FF and PCE for the PSCs based on the control, reference 1, reference 2 and target films (15 individual devices for each). It is seen that the target PSCs have a higher average PCE (24.94%) compared to that of the control PSCs (23.32%). In addition, we evaluated the stability of the unencapsulated PSCs under illumination and heat conditions according to the Organic Photovoltaic Stability (ISOS) protocols [68]. The target PSCs retain 82.9% of the initial PCEs after accelerated aging at 65 °C in a nitrogen-filled glove box (ISOS-D-1) for 600 h, whereas the control PSCs shows an over 30% efficiency loss (Fig. 5b). Under continuous 1-sun illumination at ~ 23 °C in a nitrogen-filled glove box (ISOS-L-1). The target PSCs retain 92.6% of the maximum PCEs after 503 h, while the control PSCs exhibits only 75% of the initial value (Fig. 5c). The target device can maintain 93% of its initial efficiency after MPP tracking for 500 h under 1-sun illumination (Fig. S43). It is worth noting that the contact angles for the control and target films are 54.3° and 73.1°, respectively (Fig. S44). The improved hydrophobicity is attributed to the hydrophobic nature of o-TB-GDY, which enhances both the film and hence device stability. The unencapsulated PSCs were stored in a relative humidity (RH) range of 30%–40% at ambient condition. The target PSCs maintain 90.1% of their initial PCEs over 500 h, while the control PSCs retain only 70.5% over the same period (Fig. S45).

## Conclusions

In summary, we investigated a facile and effective AAE process to produce highly efficient and stable PSCs. The novel multifunction additive for AAE, o-TB-GDY, with high *π*–electron conjugation, was employed to simultaneously enhance perovskite crystallization and passivate surface defects. We found that the AAE technique enables the deposition of o-TB-GDY at various depths within the perovskite film, forming a gradient distribution near the interface. The o-TB-GDY serves as nucleation seeds, significantly enhancing the nucleation and growth of perovskite crystals. In addition, o-TB-GDY primarily remains on the surface of the perovskite films after crystallization, where it strongly interacts with the undercoordinated Pb defects for effective passivation. As a result, the PSCs achieved a champion PCE of 25.62% (certified as 25.01%). Furthermore, the unencapsulated PSCs exhibit excellent stability under heat, moisture and light conditions. Our work offers a practical approach to improving perovskite crystallization and passivating surface defects synergistically, highlighting the great potential of graphdiyne materials in perovskite-based optoelectronics.

## Supplementary Information

Below is the link to the electronic supplementary material.

Supplementary file1 (DOCX 9784 KB)
