# Supplementary material for: Multifunctional Graphdiyne Enables Efficient Perovskite Solar Cells via Anti-Solvent Additive Engineering
Source: Nanomicro Lett. 2025 Jan 28;17:121. doi: 10.1007/s40820-024-01630-y (PMC11775377; doi:10.1007/s40820-024-01630-y)
Supplement: Supplementary file 1 — Supplementary file1 (DOCX 9784 KB) [file 40820_2024_1630_MOESM1_ESM.docx]

Supporting Information for

**Multifunctional Graphdiyne Enables Efficient Perovskite Solar Cells via Antisolvent Additive Engineering**

Cong Shao^1,2,†^, Jingyi He^1,2,†^, Jiaxin Ma^1,2^, Yirong Wang^2,3^, Guosheng Niu^1,2^, Pengfei Zhang^2,4^, Kaiyi Yang^1,2^, Yao Zhao^5^, Fuyi Wang^2,5^, Yongjun Li^1,2,^*, Jizheng Wang^1,2,^*

^1^Beijing National Laboratory for Molecular Sciences, CAS Key Laboratory of Organic Solids, Institute of Chemistry, Chinese Academy of Sciences, Beijing 100049, P. R. China

^2^University of Chinese Academy of Sciences, Beijing 100049, P. R. China

^3^CAS Key Laboratory of Engineering Plastics, Institute of Chemistry, Chinese Academy of Sciences, Beijing 100049, P. R. China

^4^CAS Key Laboratory of Photochemistry, Institute of Chemistry, Chinese Academy of Sciences, Beijing 100049, P. R. China

^5^Beijing National Laboratory for Molecular Sciences, National Centre for Mass Spectrometry in Beijing, CAS Key Laboratory of Analytical Chemistry for Living Biosystems, Chinese Academy of Sciences, Beijing 100049, P. R. China

† Cong Shao and Jingyi He contributed equally to this work.

*Corresponding authors. E-mail: [liyj@iccas.ac.cn](mailto:liyj@iccas.ac.cn) (Yongjun Li); [jizheng@iccas.ac.cn](mailto:jizheng@iccas.ac.cn) (Jizheng Wang)

**Supplementary Figures and Tables**


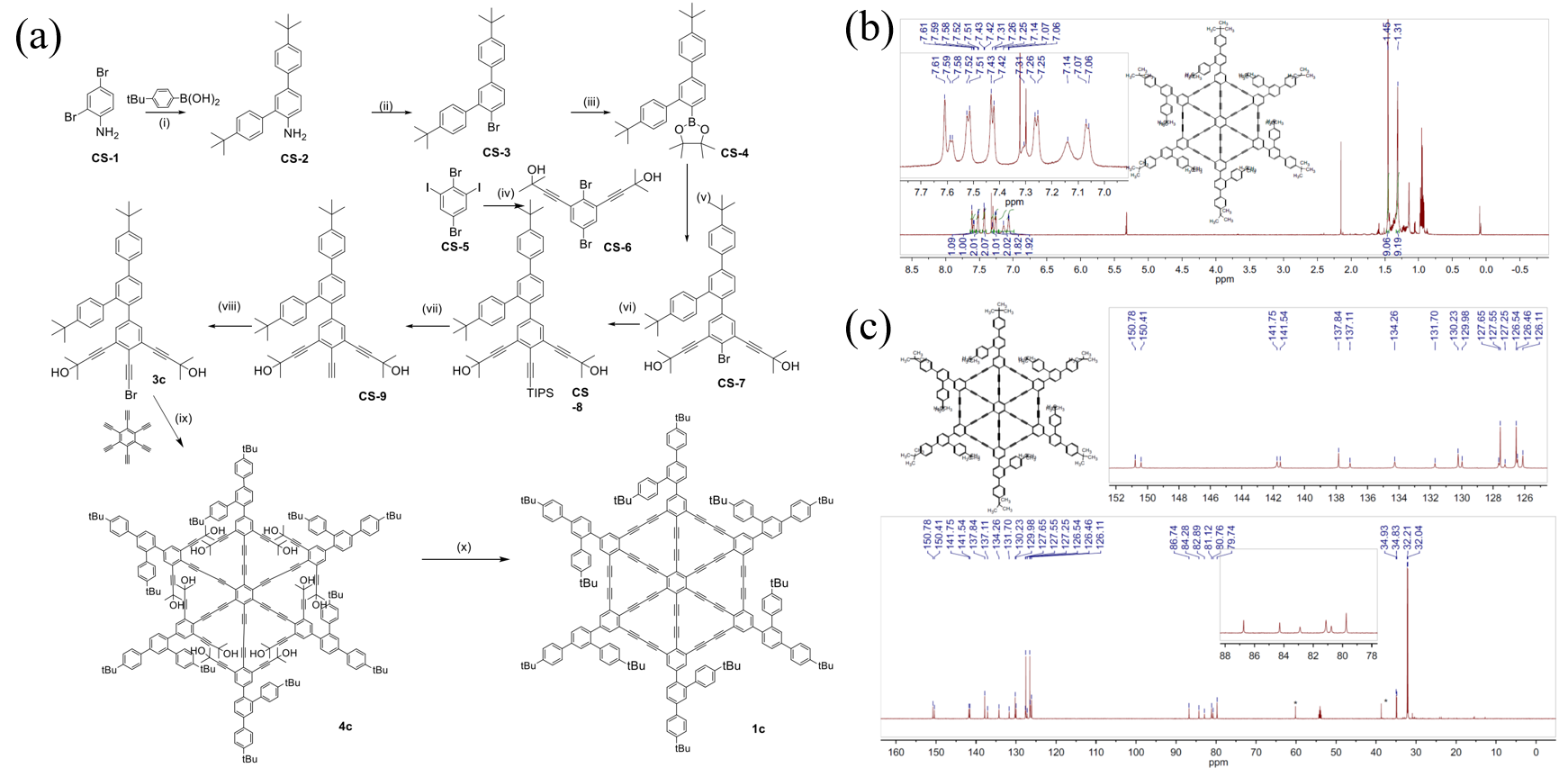


**Fig. S1 a** Reaction scheme of o-TB-GDY (1c) synthesis. **b, c** ^1^H NMR and ^13^C NMR spectra of resultant product. ^1^H NMR (700 MHz, CS_2_/CD_2_Cl_2_ (25/1 v/v), 298 K) δ 7.61 (s, 6H), 7.59 (d, 6H), 7.52 (d, J = 7.0 Hz, 12H), 7.43 (d, J = 7.5 Hz, 12H), 7.32–7.29 (m 6H), 7.26 (d, J = 8.1 Hz, 12H), 7.14 (s, 12H), 7.07 (d, 12H), 1.45 (s, 54H), 1.31 (s, 54H). ^13^C NMR (176 MHz, CS_2_/CD_2_Cl_2_ (25/1 v/v), 298 K) δ 150.78, 150.41, 141.75, 141.54, 137.84, 137.11, 134.26, 131.70, 130.23, 129.98, 127.65, 127.55, 127.25, 126.54, 126.46, 126.11, 86.74, 84.28, 82.89, 81.12, 80.76, 79.74, 34.93, 34.83, 32.21, 32.04. For more details, it can be found in previous report (*J. Am. Chem. Soc.* **2023**, 145, 5400−5409)


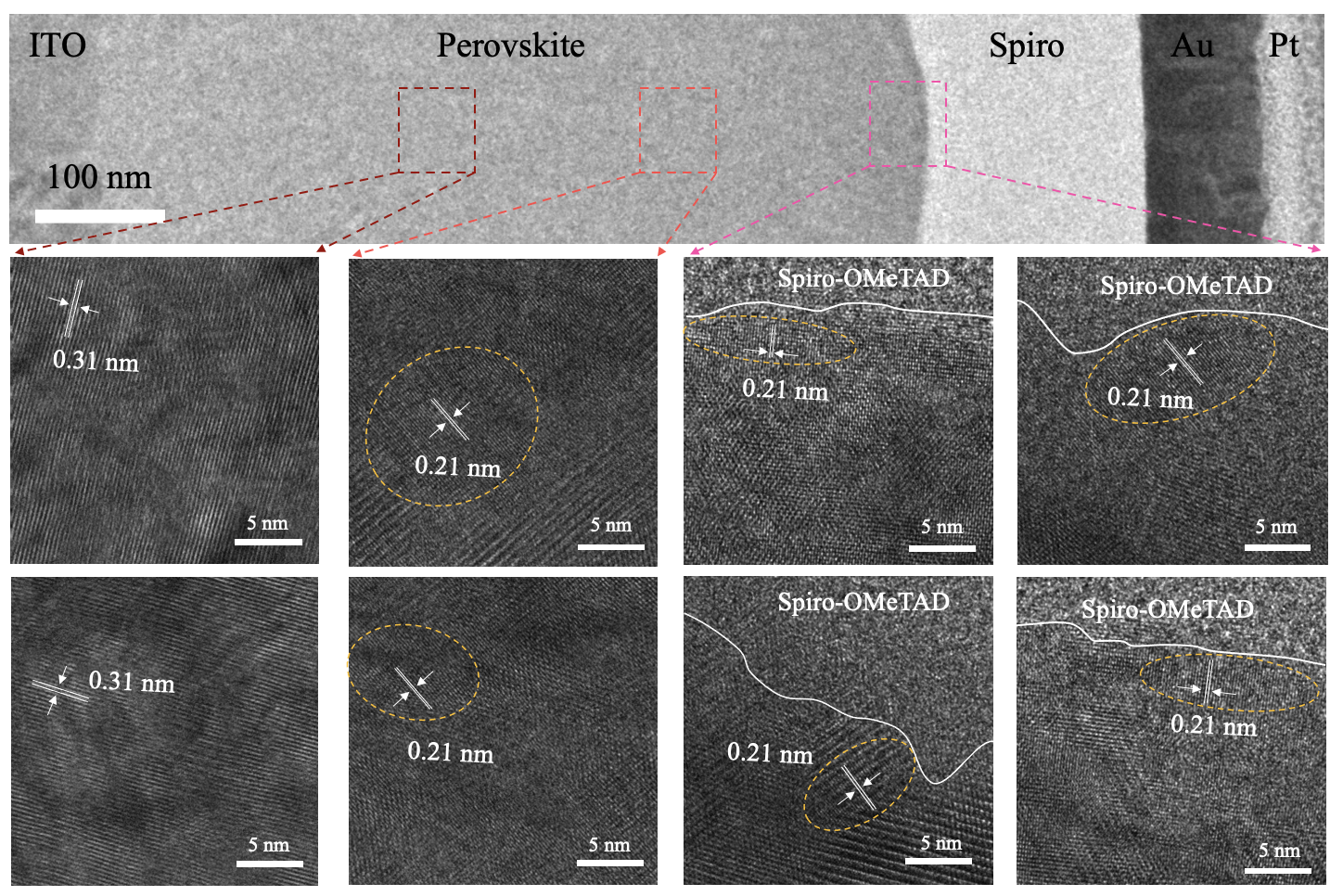


**Fig. S2** Cross- sectional HRTEM image of the target PSC. The inset shows the enlarged TEM image of the region enclosed by three boxes. The areas enclosed by the dashed yellow lines show the introduced o-TB-GDY. The distances measured show the lattice parameters for o-TB-GDY (0.21 nm) and 3D perovskites (0.31 nm)


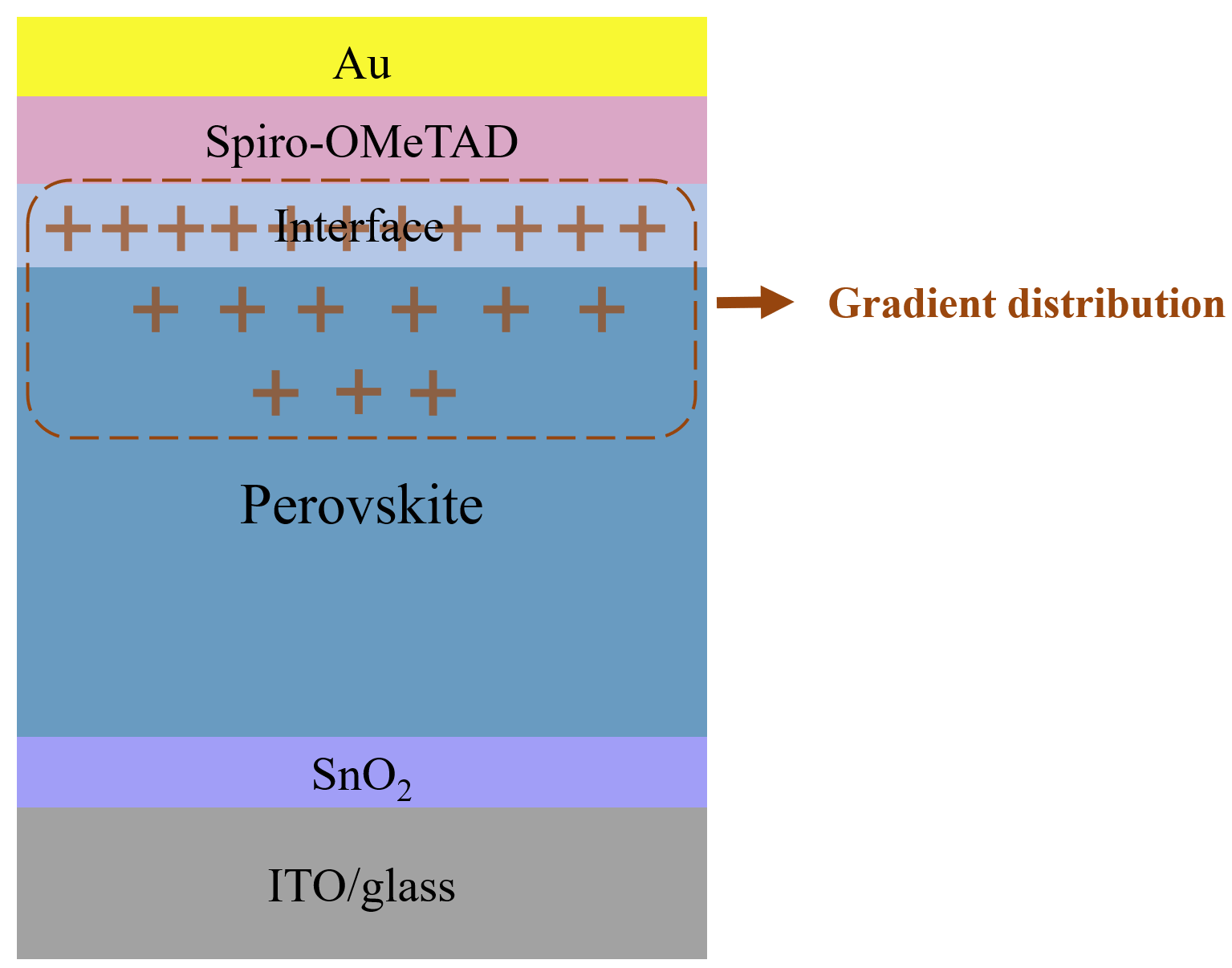


**Fig. S3** Schematic of the target device. The plus symbols represent o-TB-GDY in the PSC


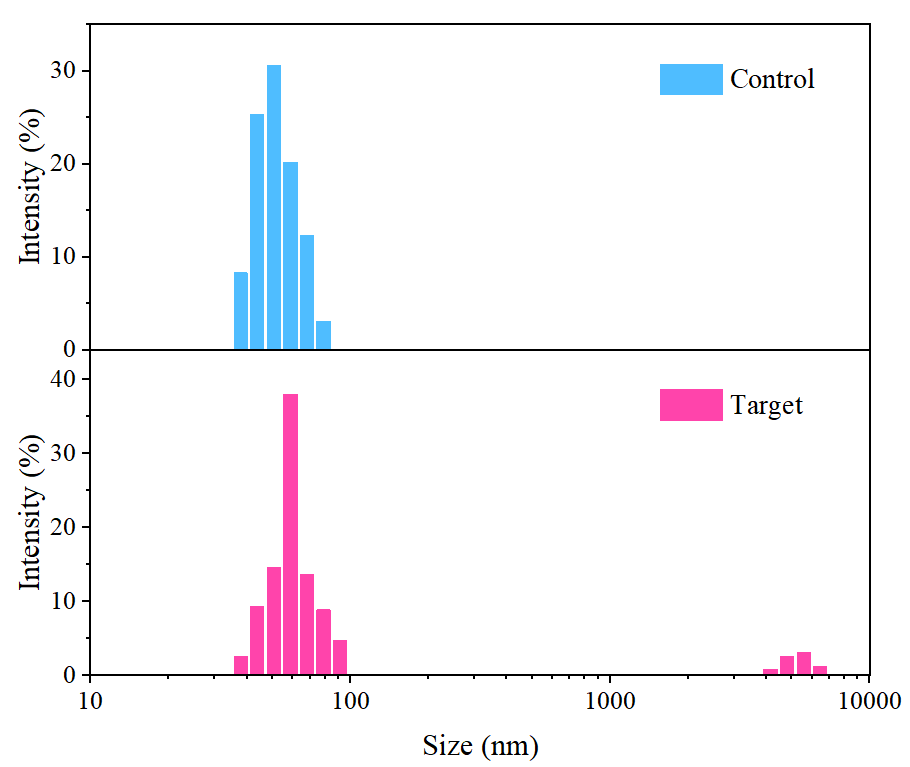


**Fig. S4** DLS spectra of control and target precursor solutions


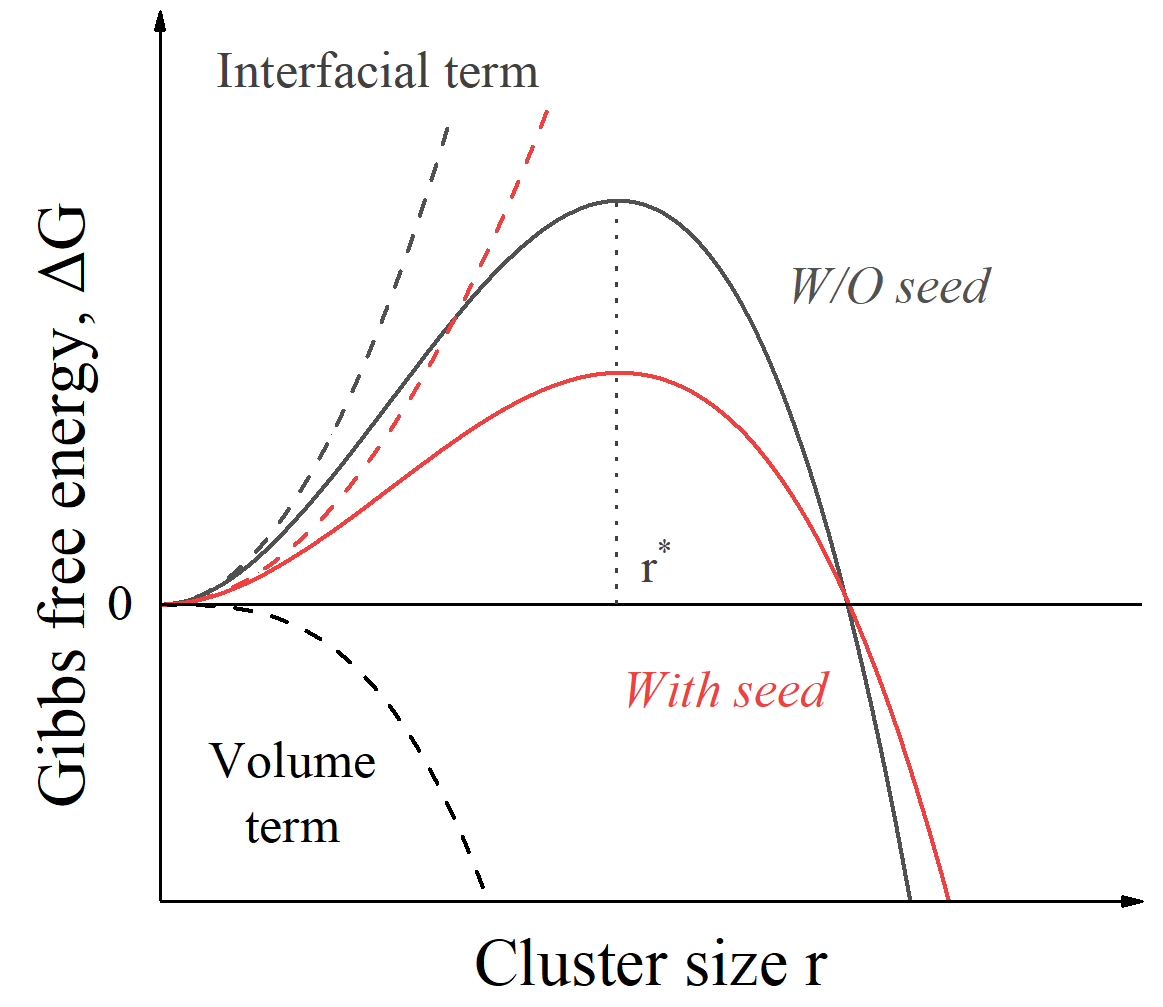


**Fig. S5** Gibbs free energies of perovskite nucleation with and without seed


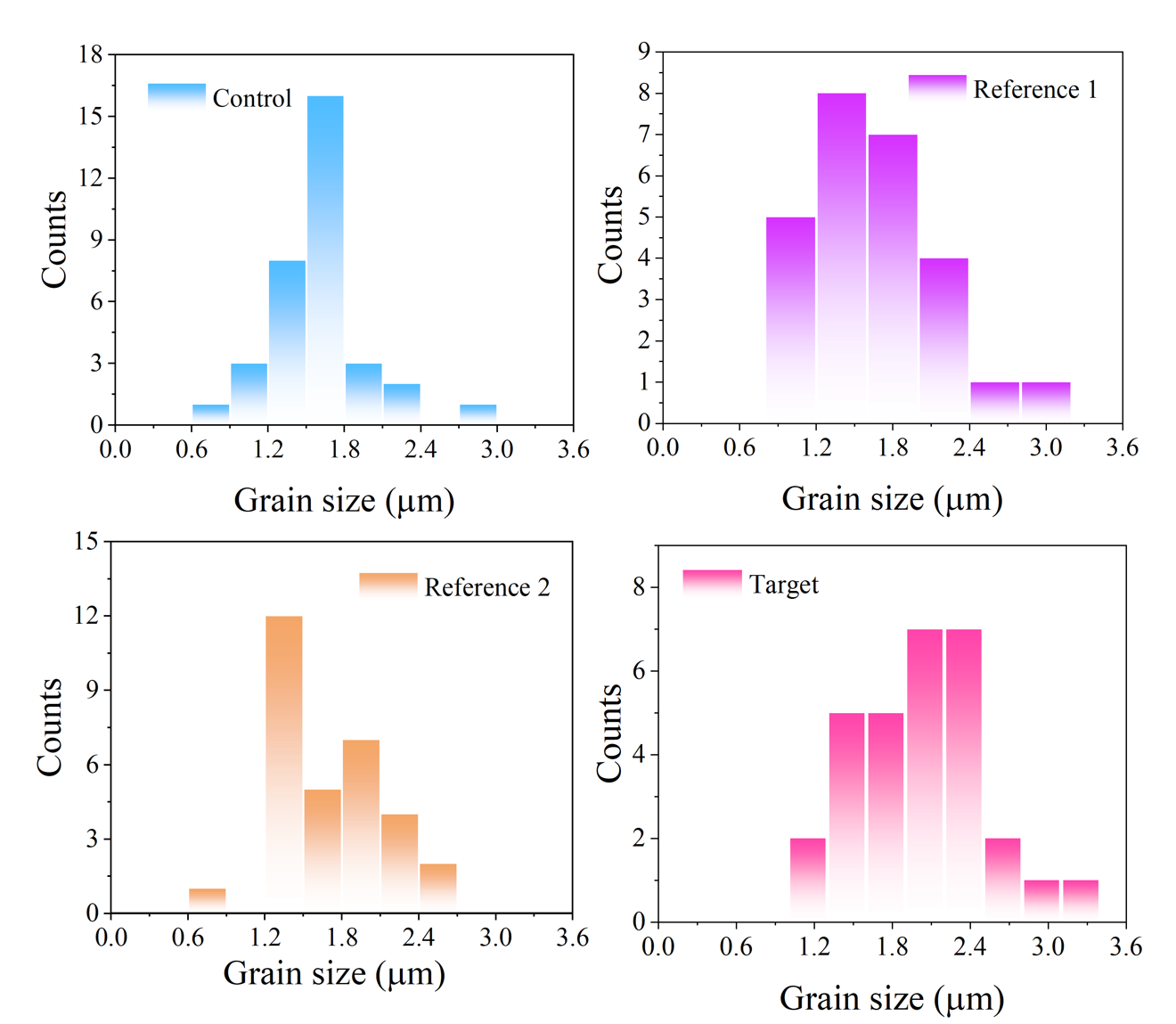


**Fig. S6** Grain size distributions in the control, reference 1, reference 2, and target perovskite films


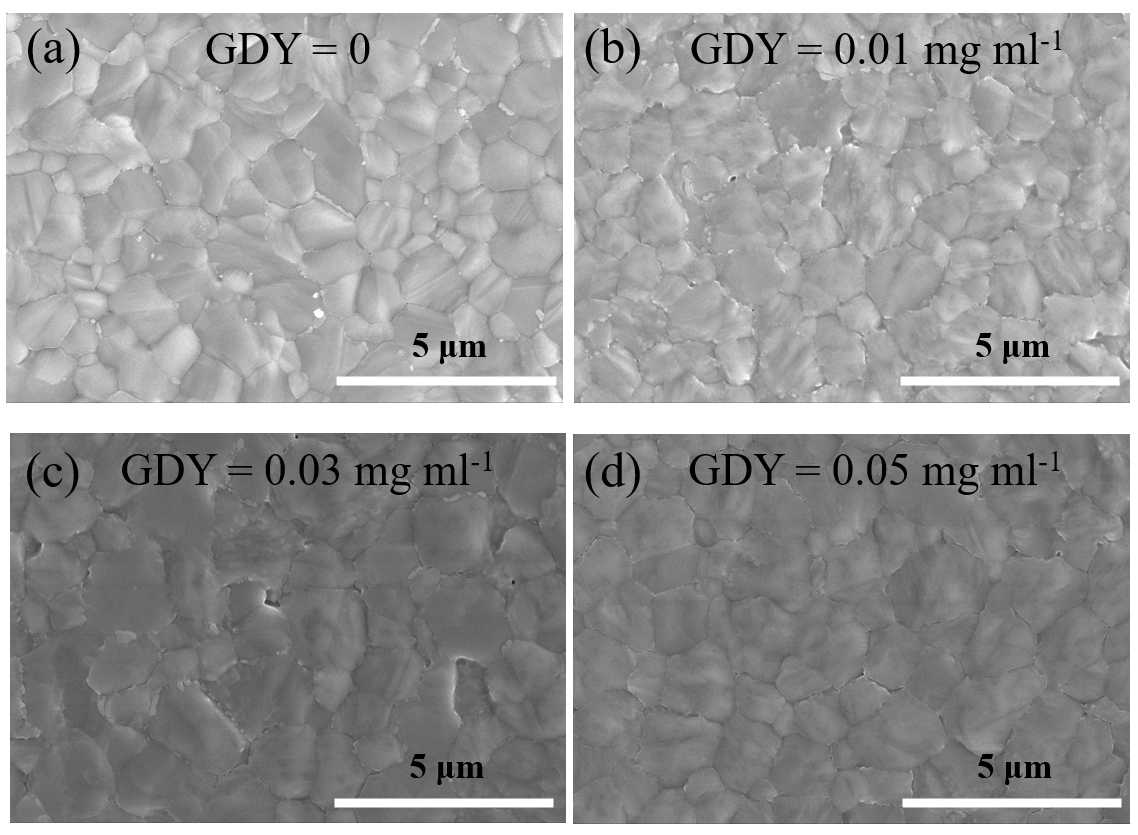


**Fig. S7** Surface morphologies of the perovskite films with different o-TB-GDY loadings **a** 0, **b** 0.01 mg ml^-1^, **c** 0.03 mg ml^-1^ and **d** 0.05 mg ml^-1^


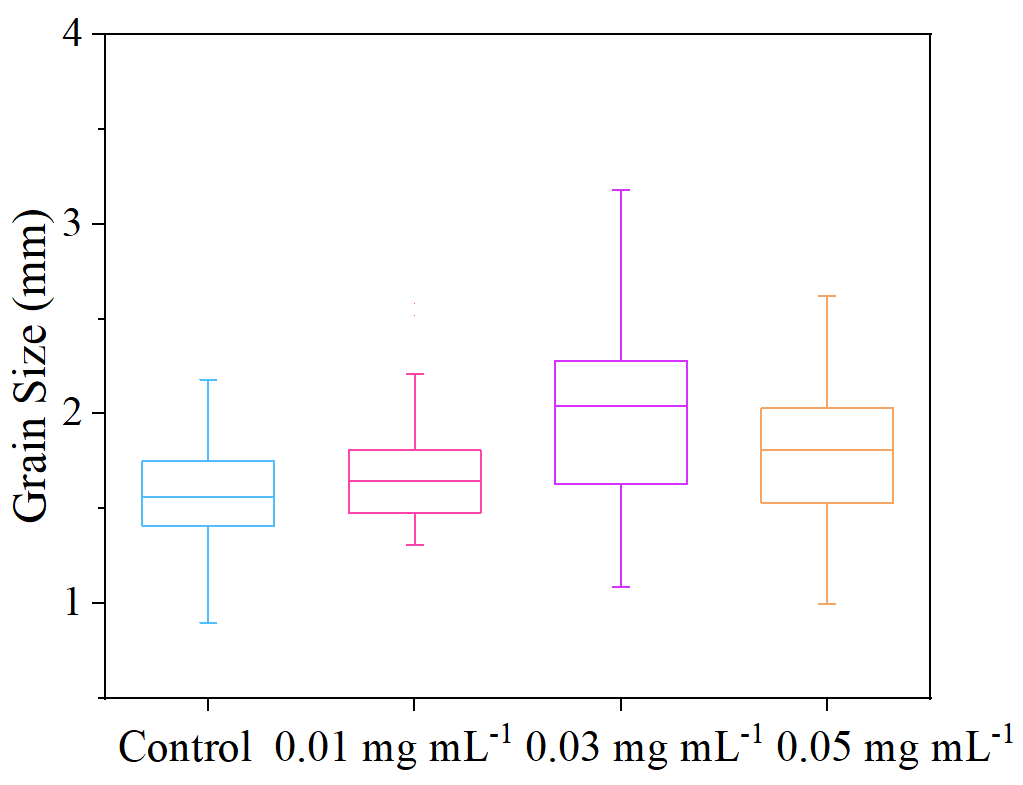


**Fig. S8** Grain size distributions in the perovskite films with different o-TB-GDY loadings (0, 0.01 mg ml^-1^, 0.03 mg ml^-1^ and 0.05 mg ml^-1^)


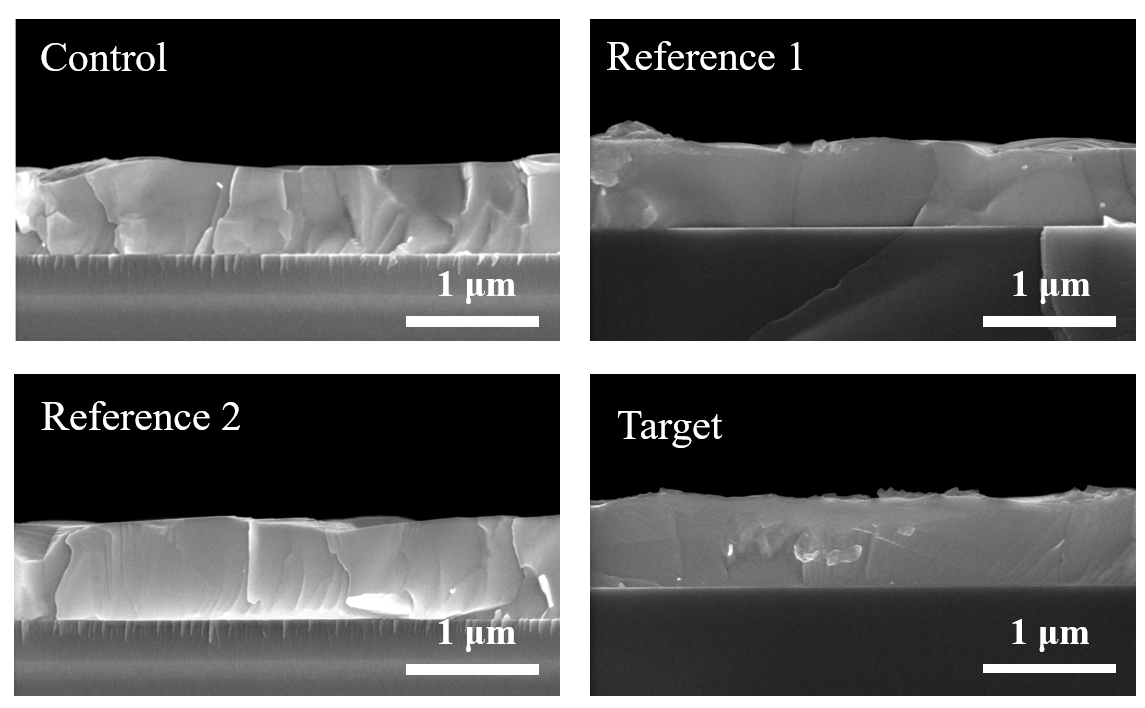


**Fig. S9** Cross-sectional SEM images of the control, reference 1, reference 2 and target films


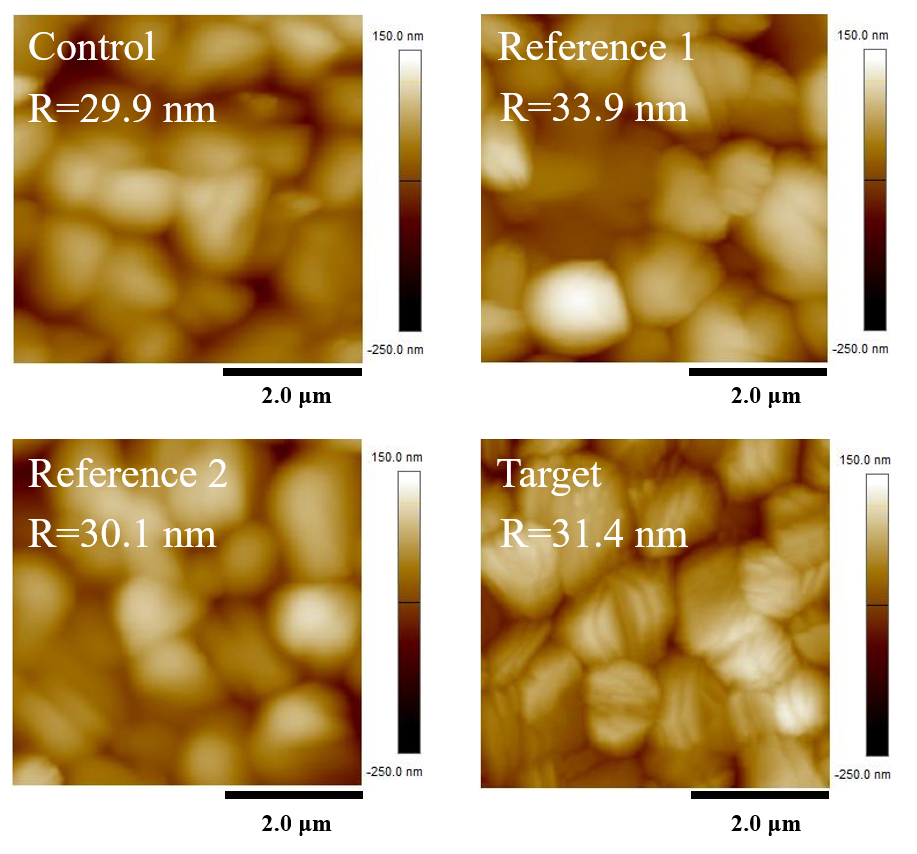


**Fig. S10** AFM images of the control, reference 1, reference 2 and target films. The corresponding surface roughness is around 30 nm for all the four films


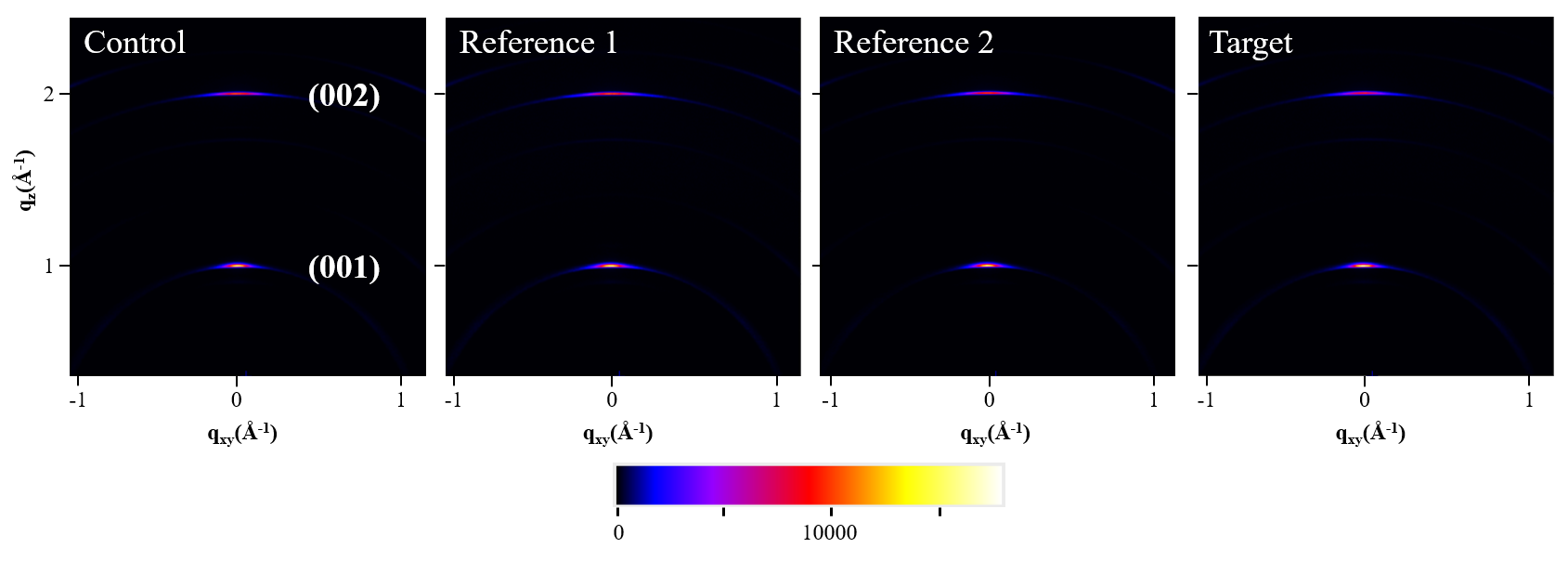


**Fig. S11** 2D-XRD images of the control, reference 1, reference 2 and target films. Reflections at q_z_ ≈ 1 and 2 Å^−1^ are assigned to perovskite (001) and (002), respectively


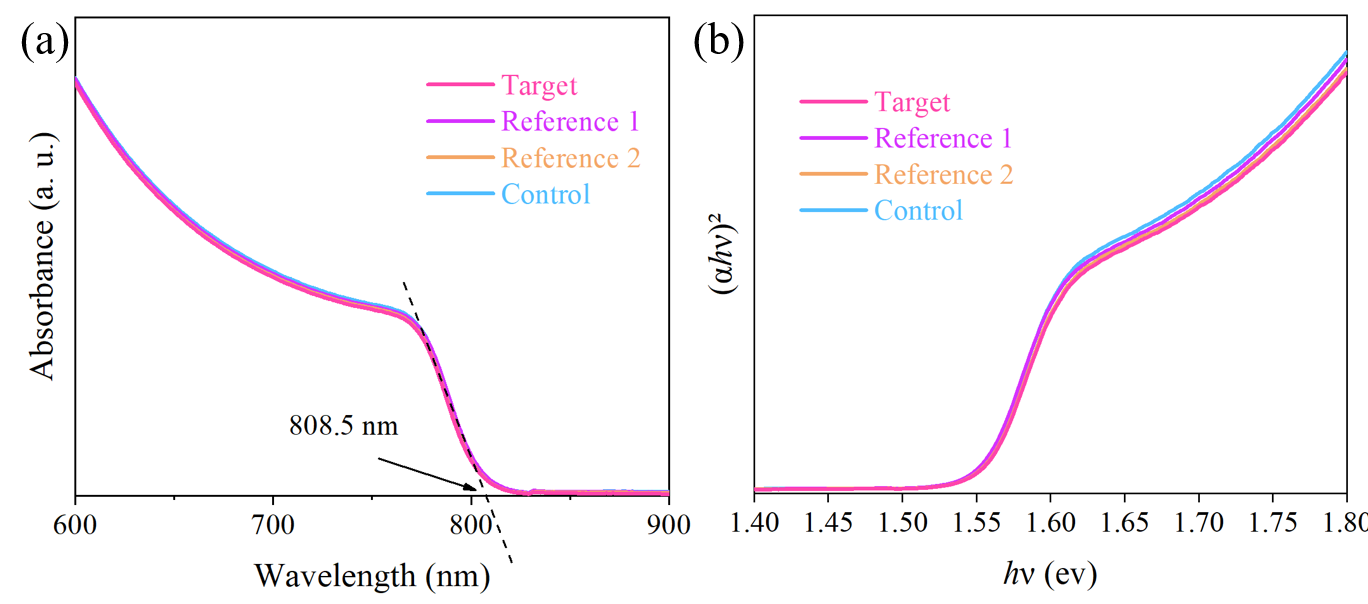


**Fig. S12** **a** UV-vis absorption spectra of the control, reference 1, reference 2 and target films. **b** Tauc plots of the corresponding films


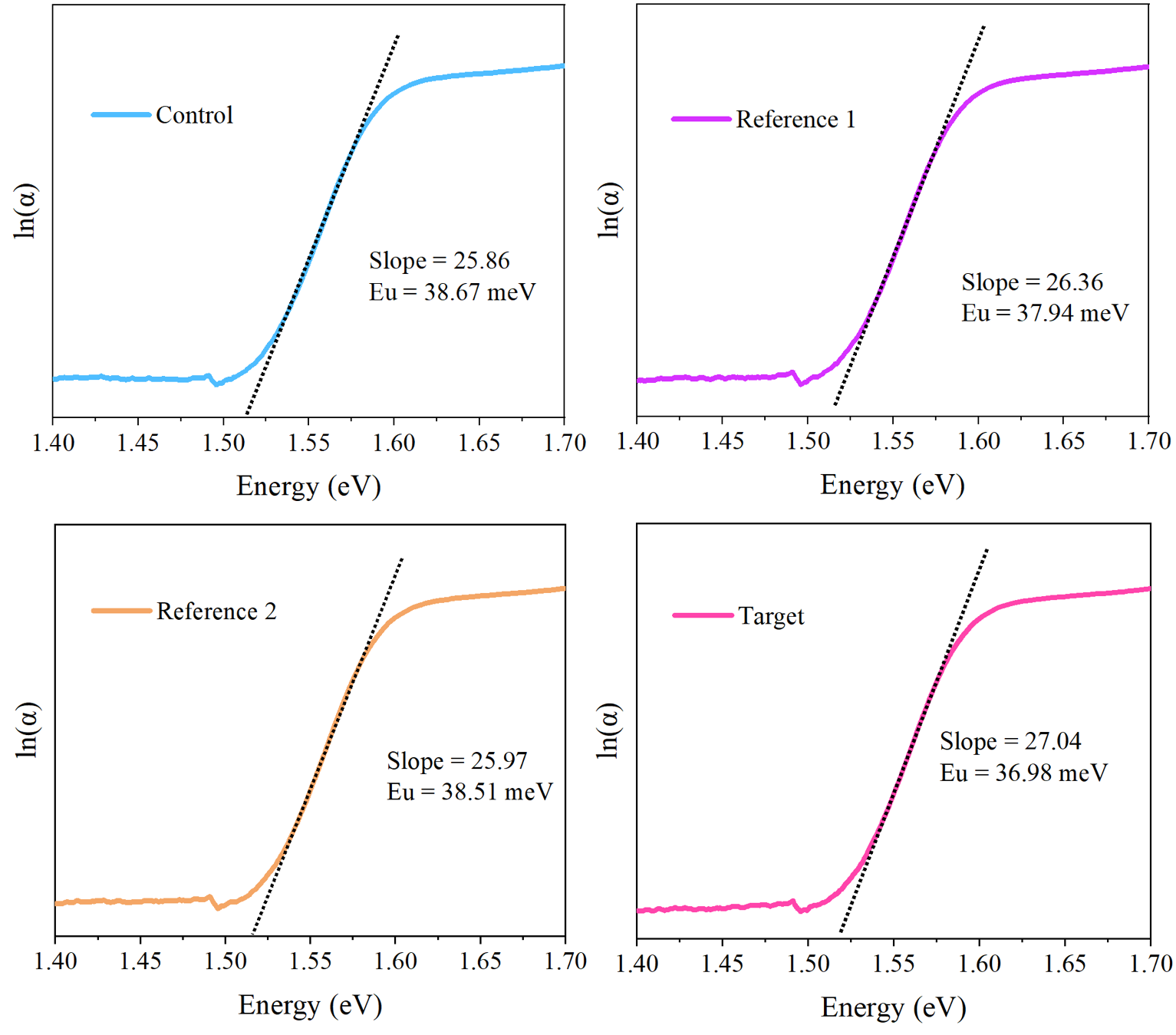


**Fig. S13** Urbach energies obtained from the control, reference 1, reference 2 and target films


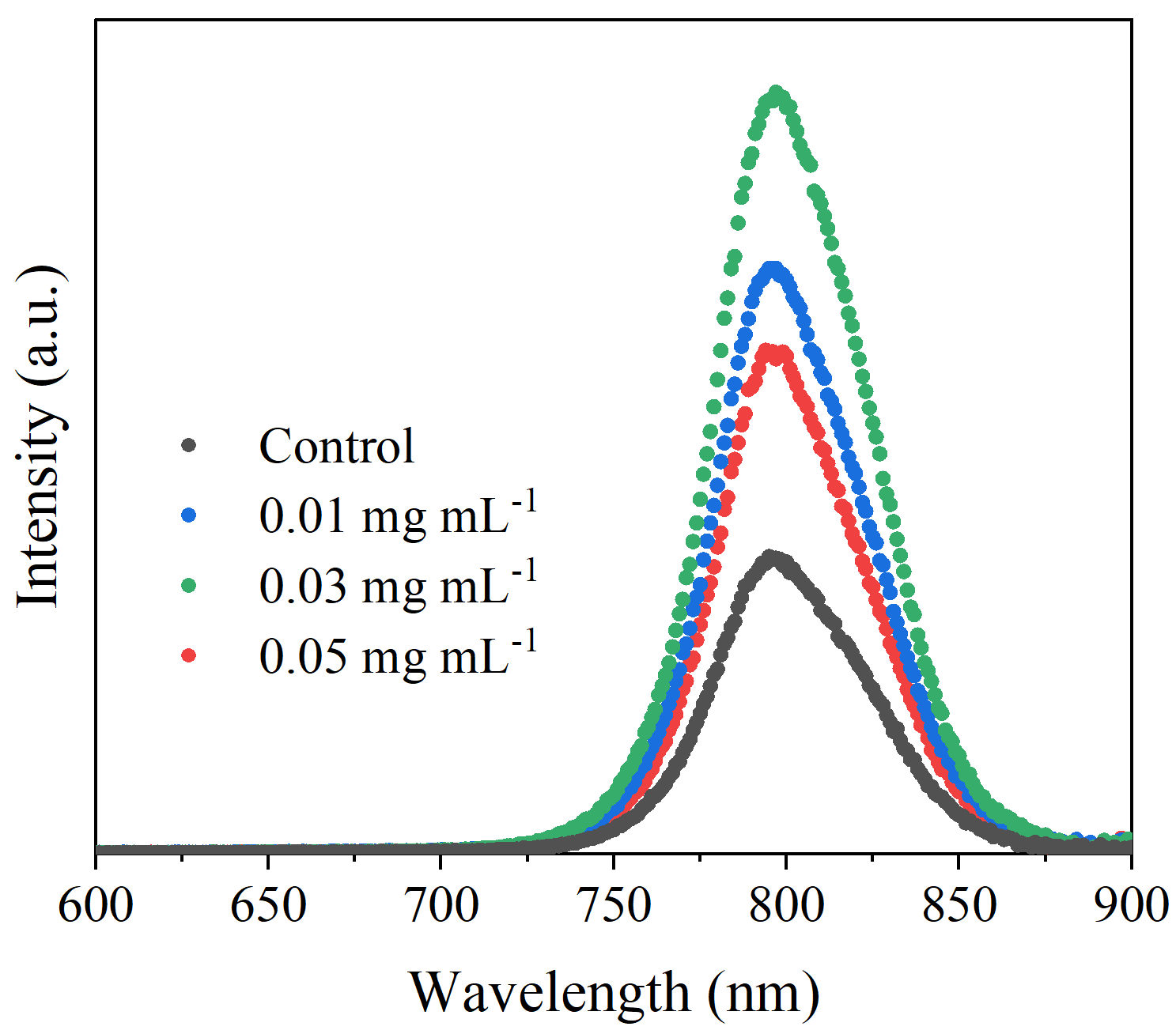


**Fig. S14** Steady-state PL spectra of the perovskite films with different o-TB-GDY loadings


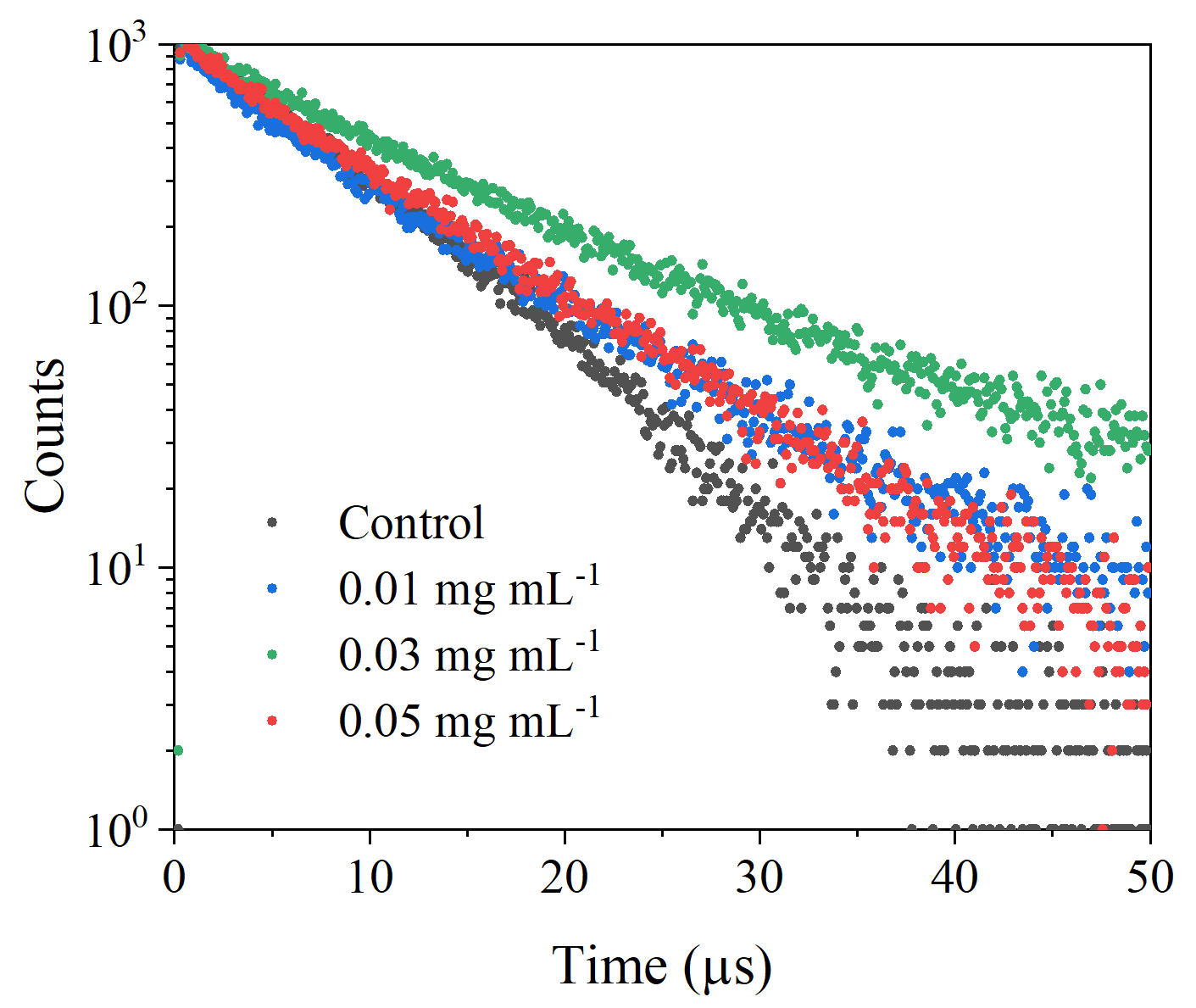


**Fig. S15** Time-resolved PL spectra of the perovskite films with different o-TB-GDY loadings.


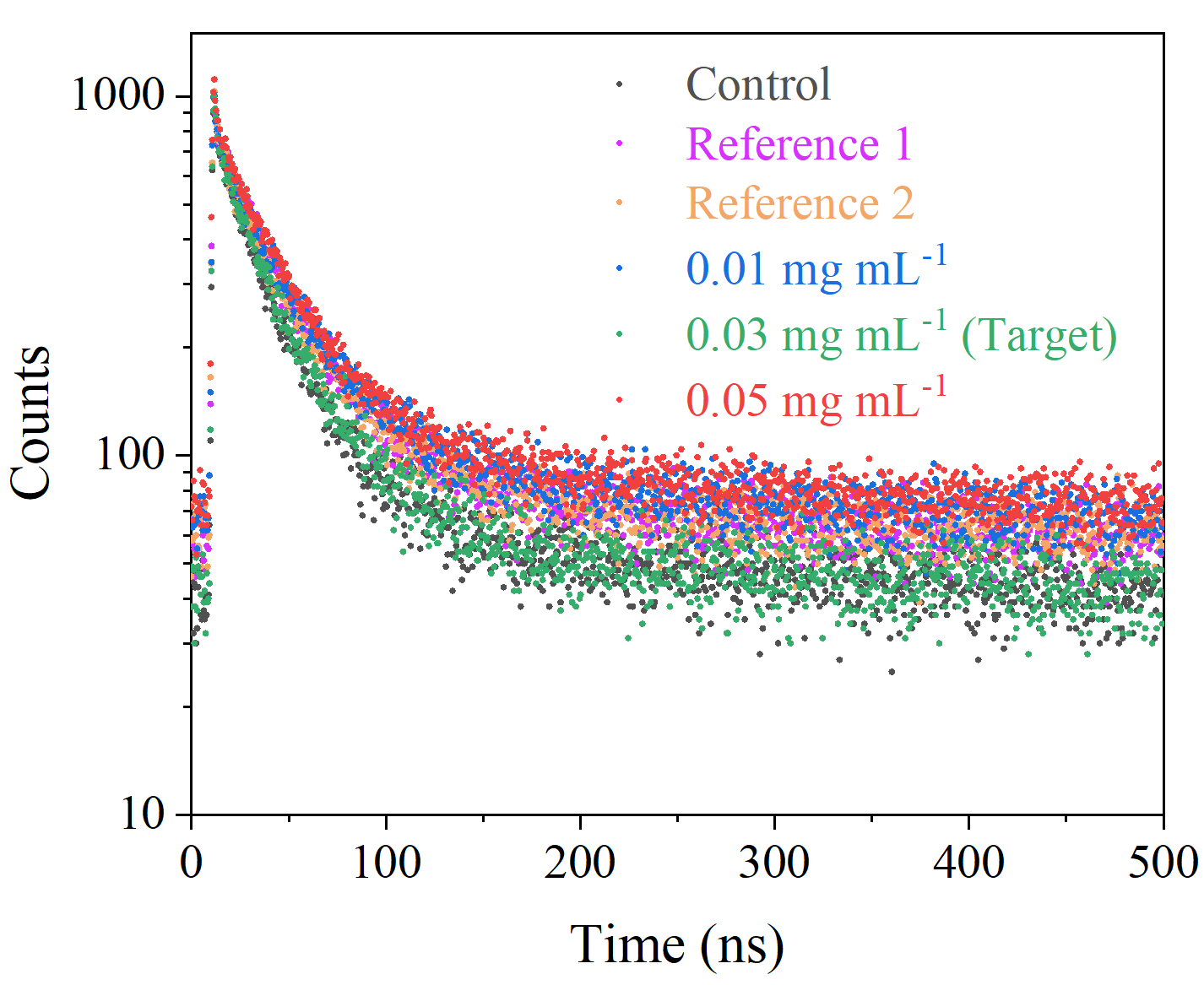


**Fig. S16** Time-resolved PL spectra of different perovskite films with spiro-OMeTAD atop


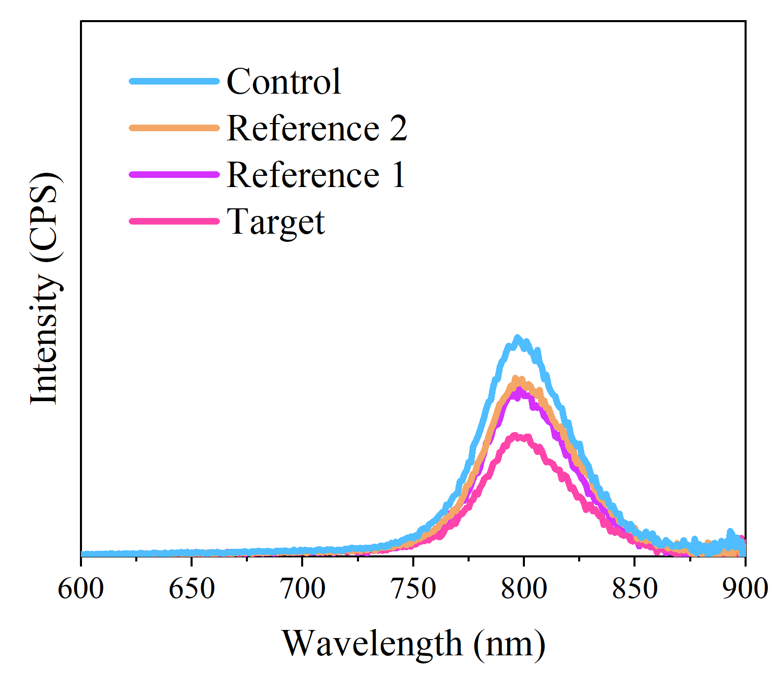


**Fig. S17** Steady-state PL spectra of different perovskite films with spiro-OMeTAD


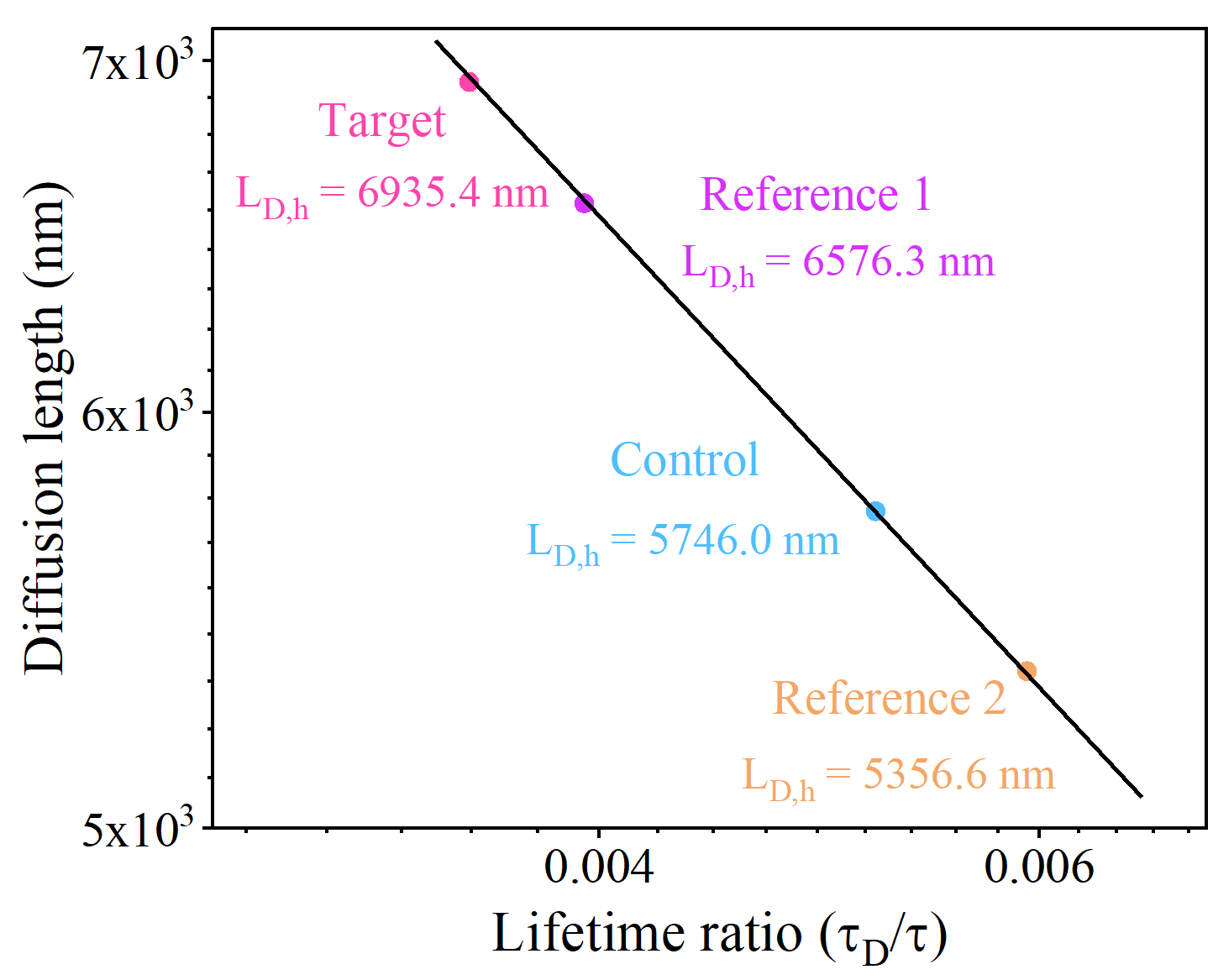


**Fig. S18** The charge-carrier diffusion length of the control, reference 1, reference 2, and target films


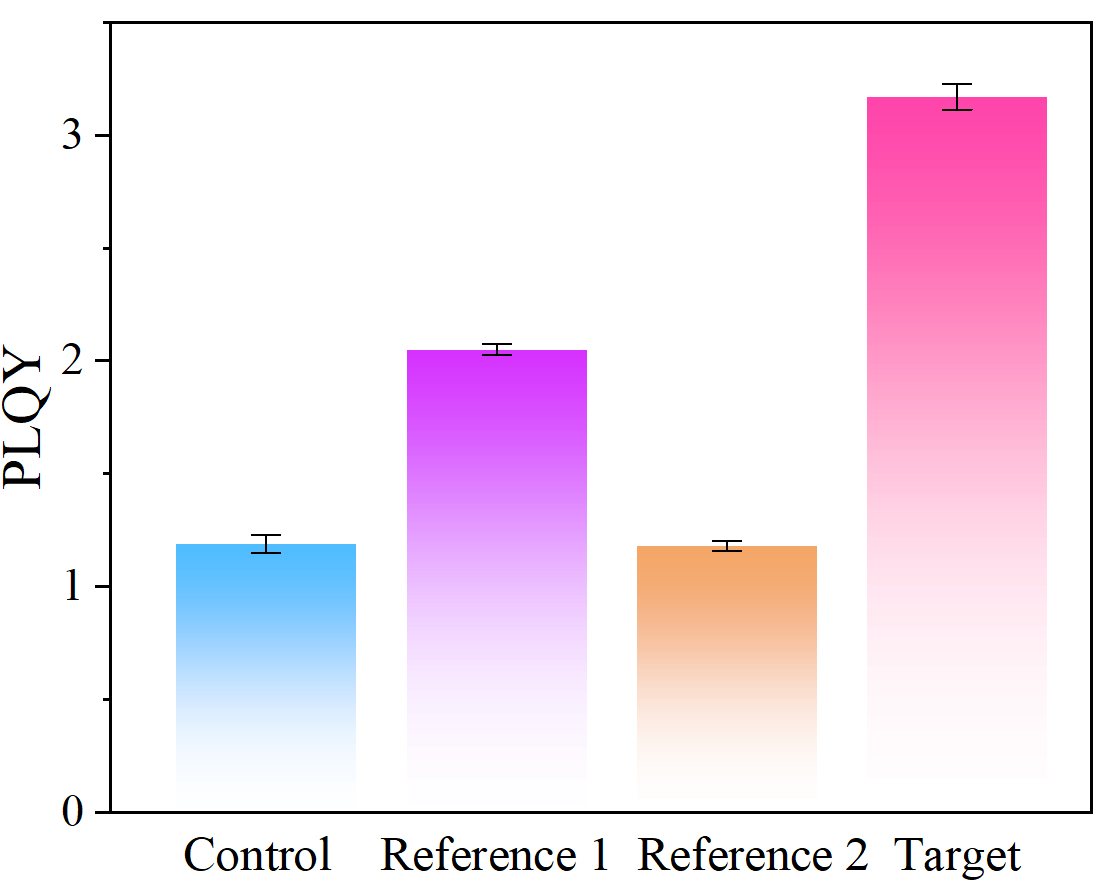


**Fig. S19** Photoluminescence quantum yield (PLQY) of the control, reference 1, reference 2, and target films. Data are presented as mean values ± s.e.m


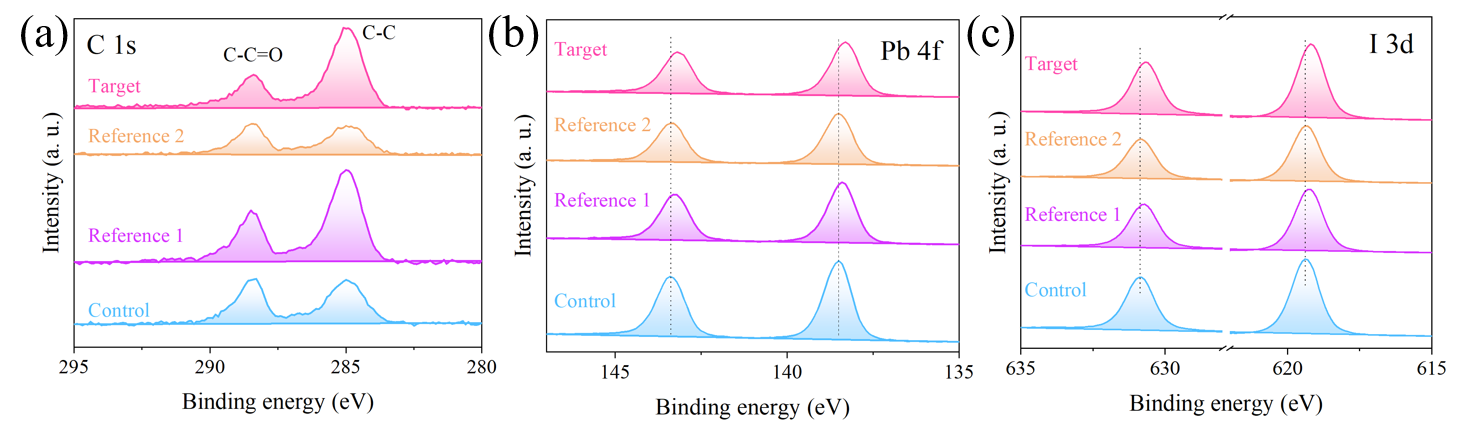


**Fig. S20** XPS spectra of the control, reference 1, reference 2, and target films **a** C 1s, **b** Pb 4f, **c** I 3d


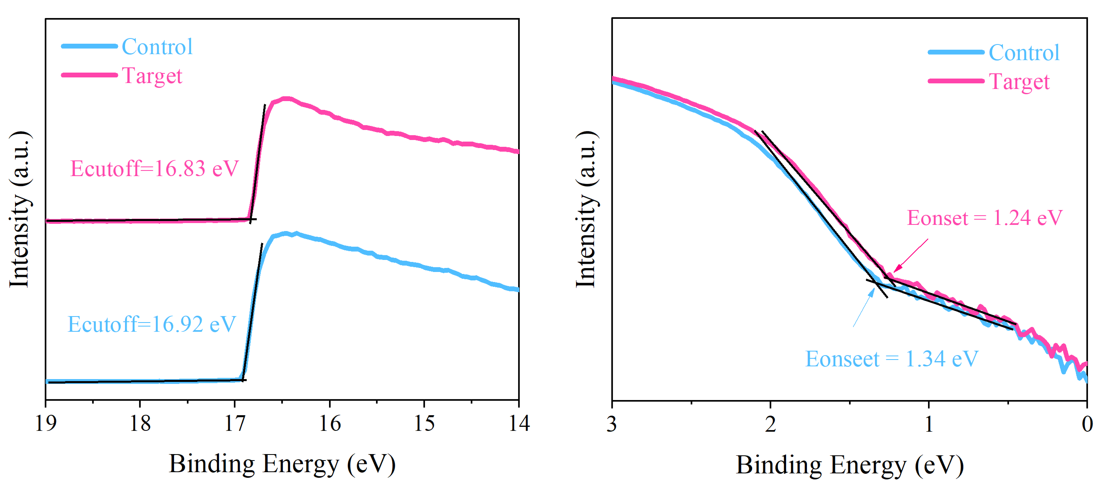


**Fig. S21** UPS measurements for the control and target films


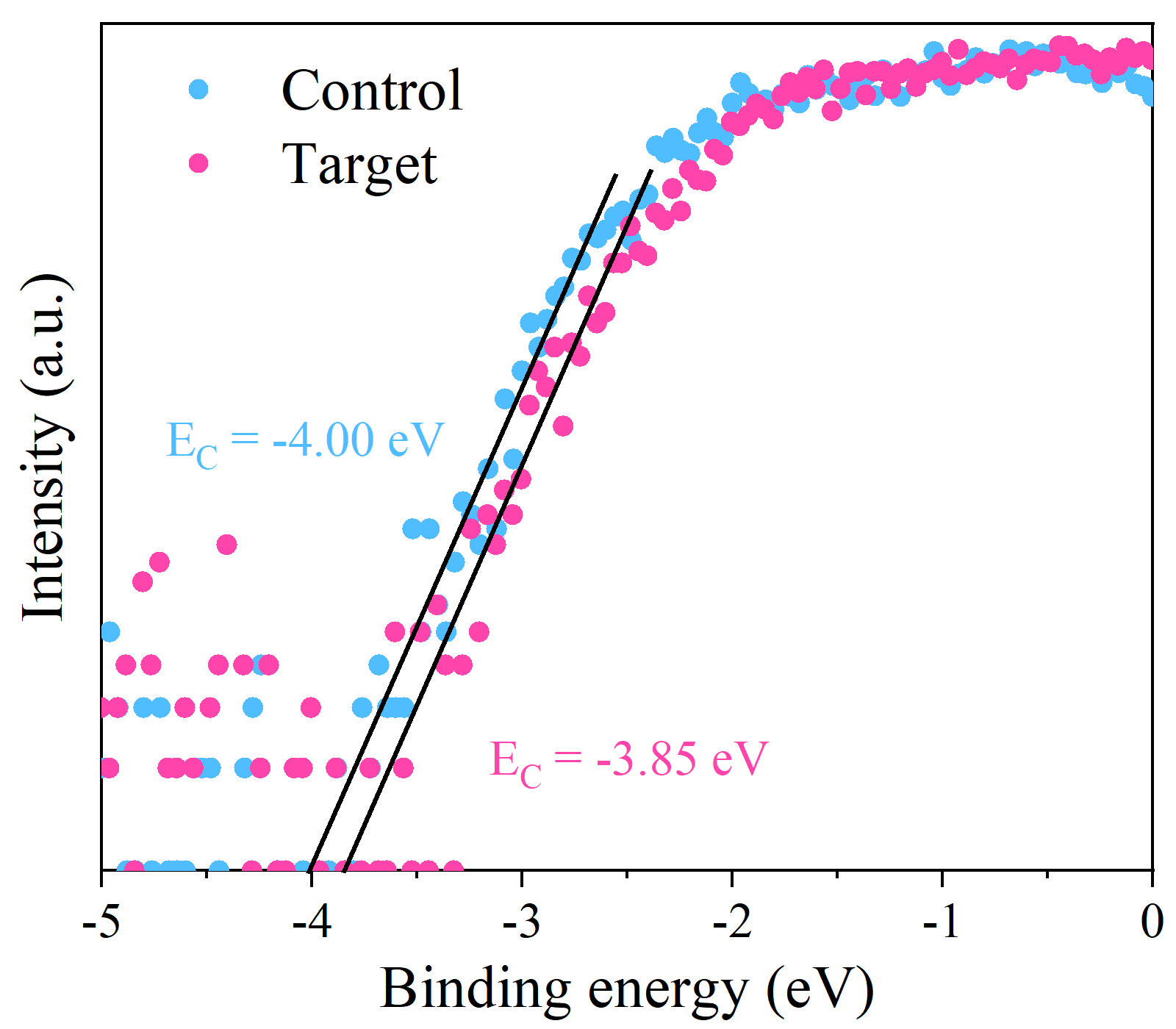


**Fig. S22** LEIPS spectra of the control and target films


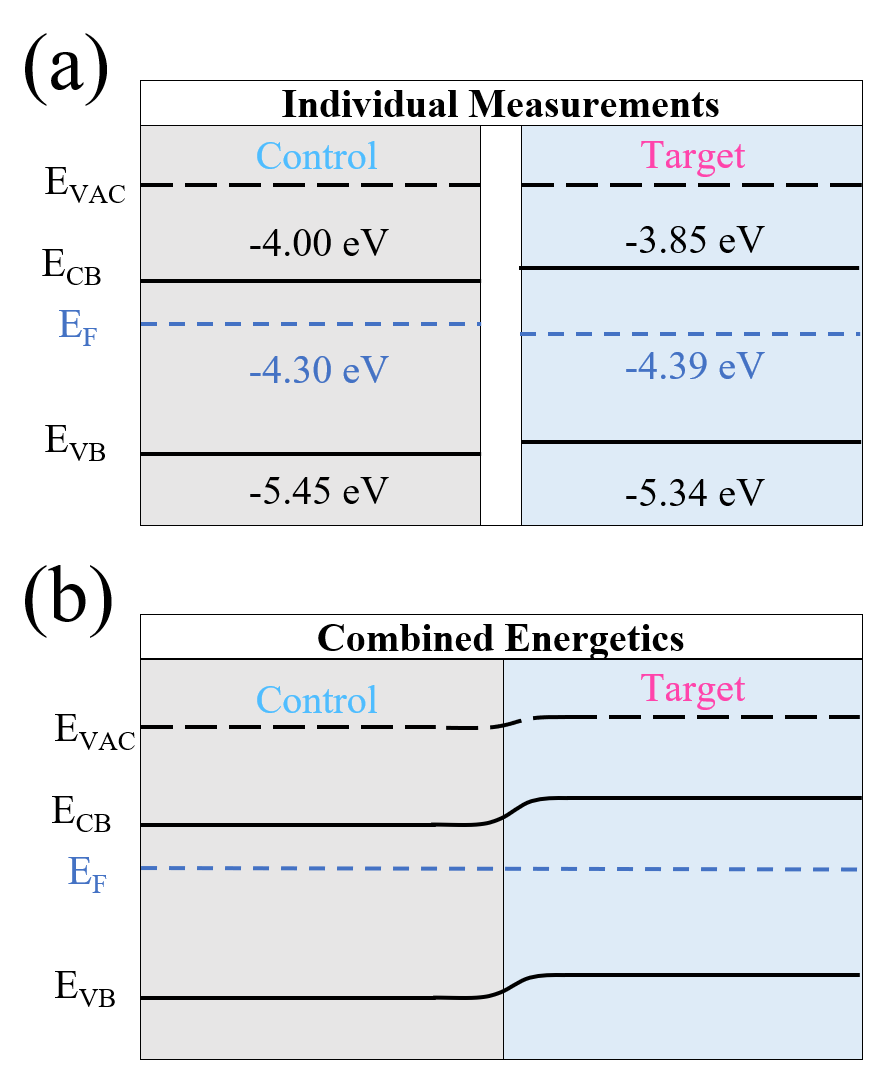


**Fig. S23** Schematics of energy level diagram. **a** Individual measurements of energy levels of the control and target films and **b** the corresponding band-bending schematics


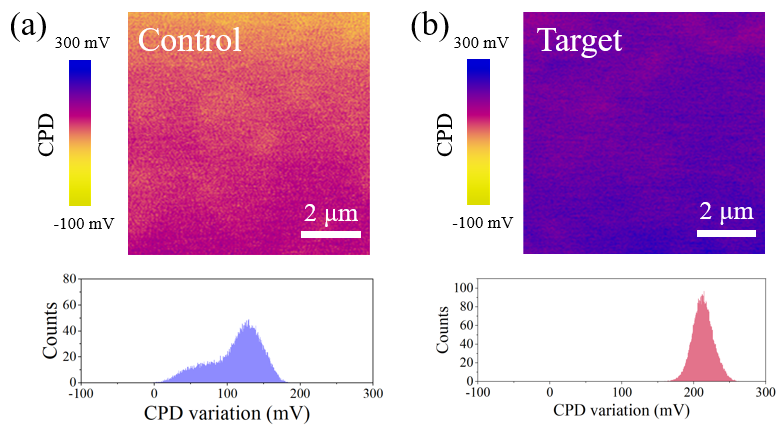


**Fig. S24** KPFM images and the statistical surface potential distributions of the **a** control and **b** target films


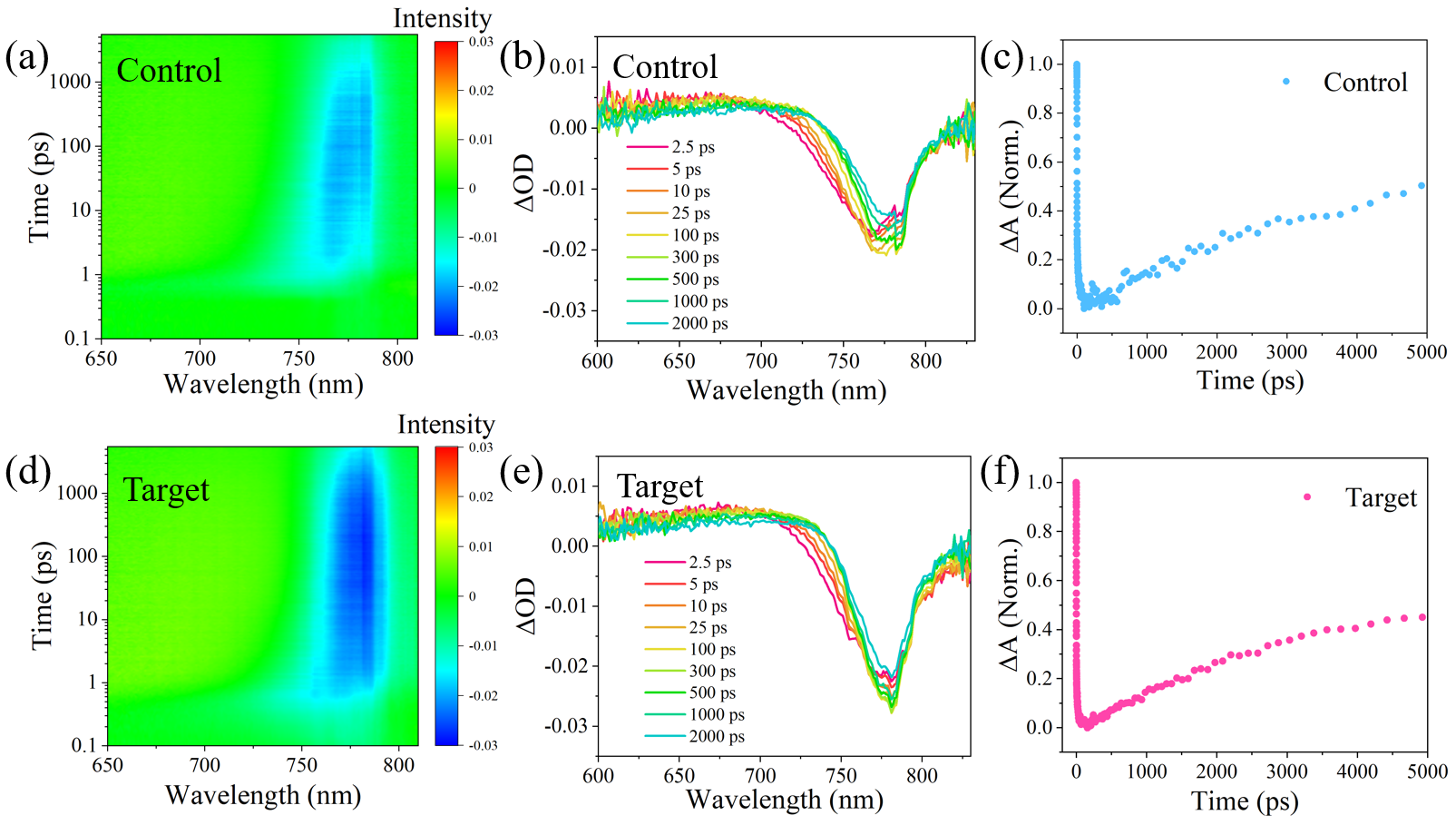


**Fig. S25** TA mapping of the **a** control and **d** target films, TA spectra at different delay times of the **b** control and **e** target films under front excitation, Decay kinetics at 783 nm of the **c** control and **f** target films


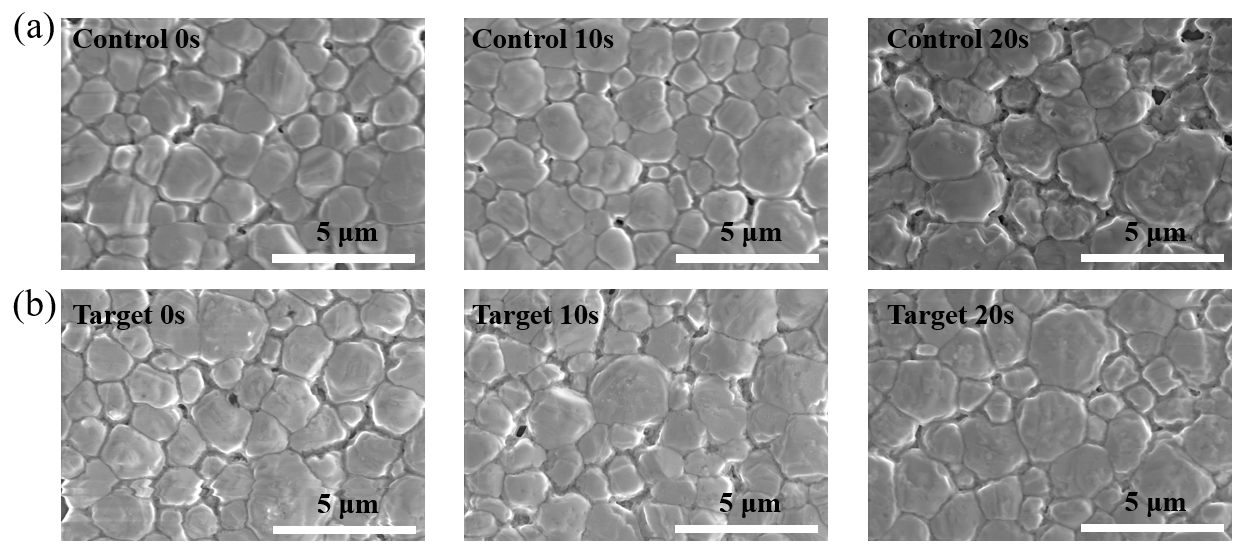


**Fig. S26** Top view SEM images of **a** control film and **b** target film annealing for different times (0s, 10s and 20s)


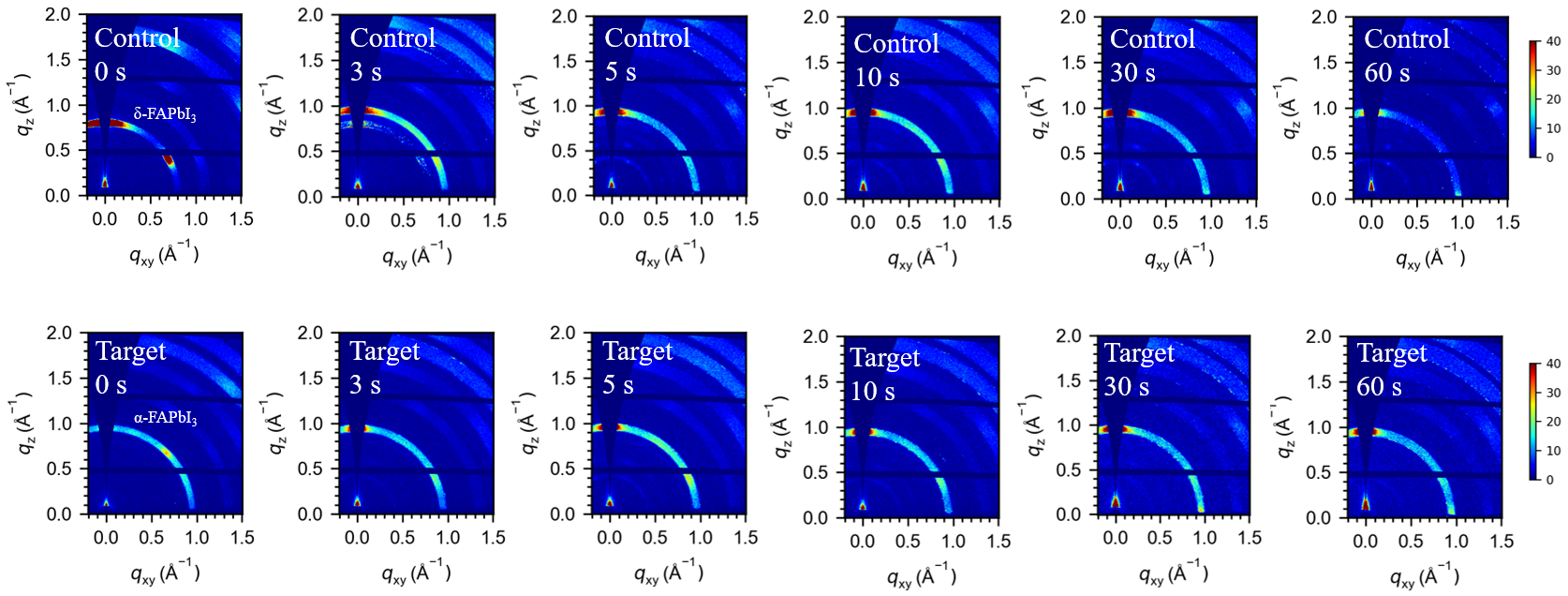


**Fig. S27** In situ GIWAXS measurement of the **a** control and **b** target films for different annealing times (0, 3, 5, 10, 30 and 60 s)


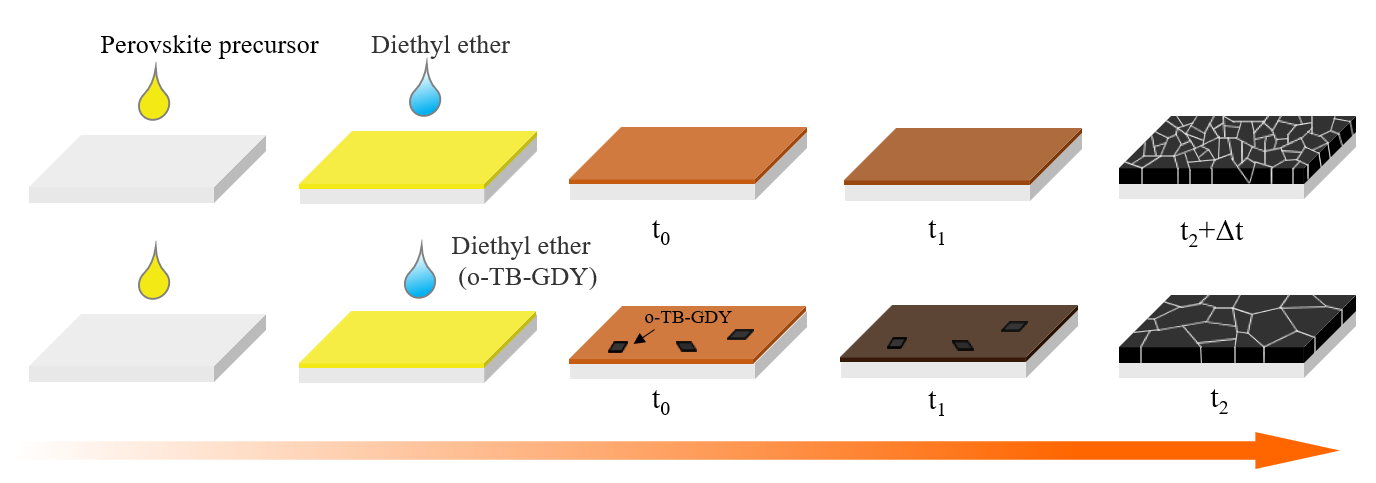


**Fig. S28** The schematic illustration of crystallization process


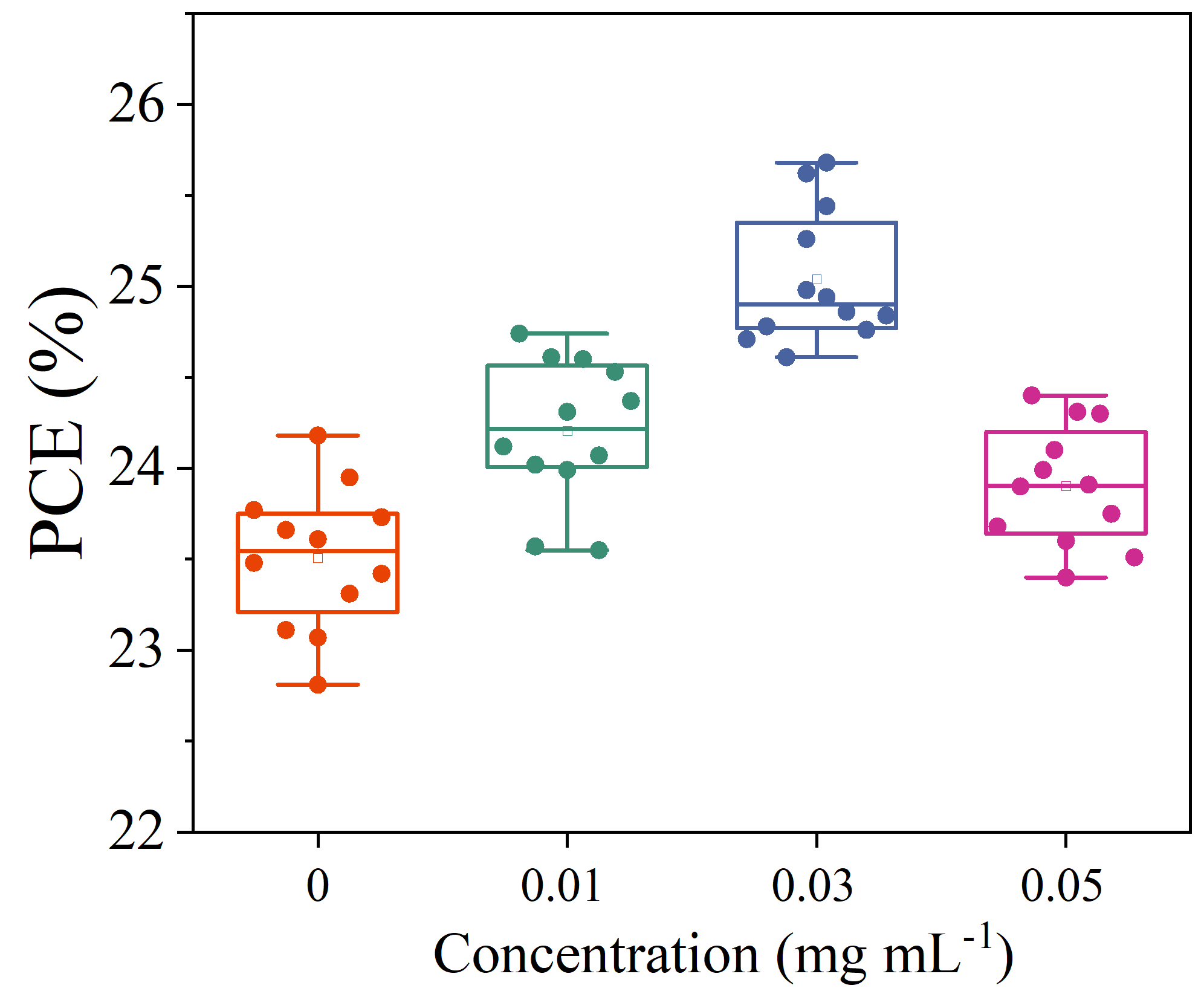


**Fig. S29** Statistical distributions of PCE for 12 PSCs with different concentrations of o-TB-GDY


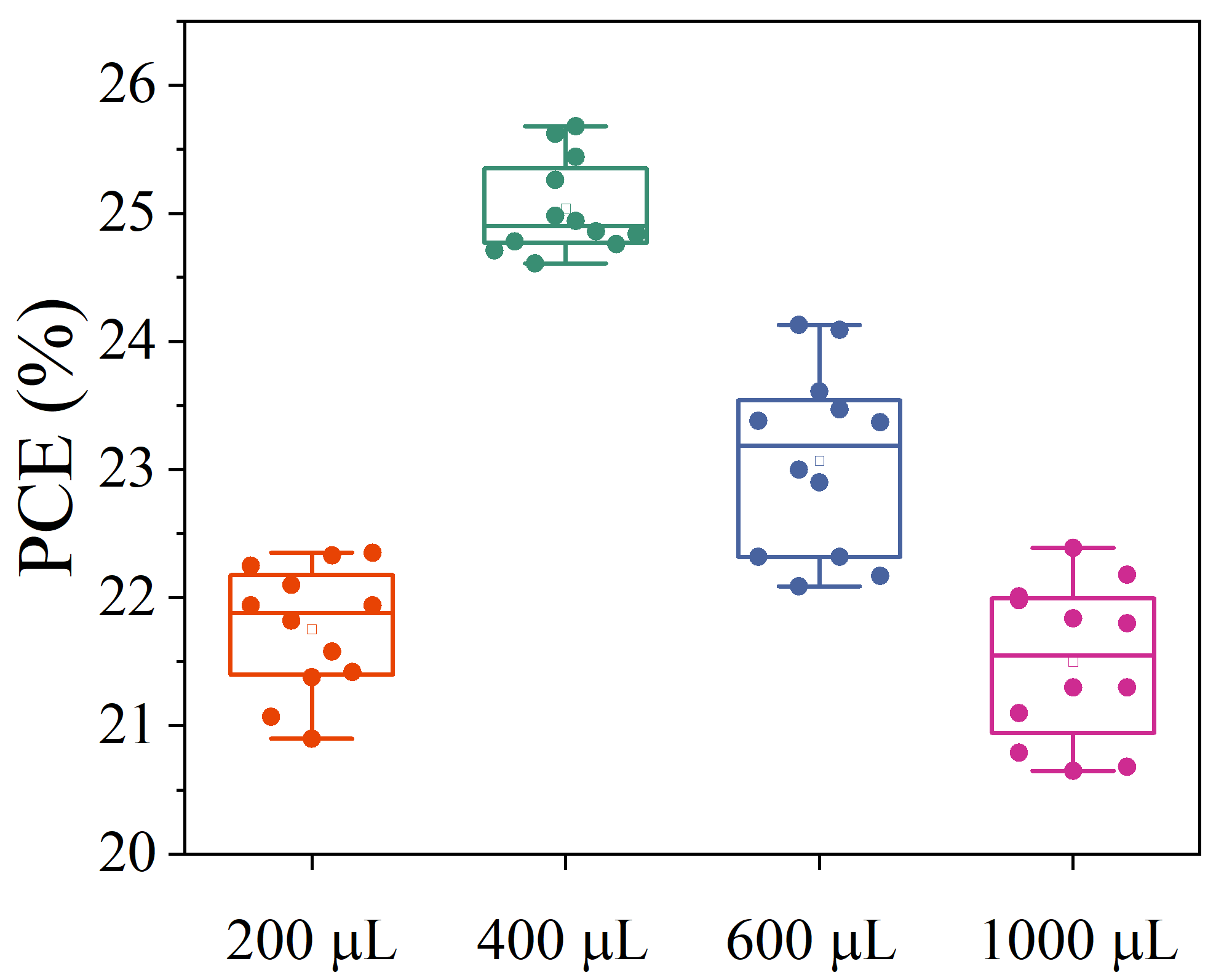


**Fig. S30** Statistical distributions of PCE for 12 PSCs based on different dosage of antisolvent (0.03 mg mL^-1^ o-TB-GDY in DE: CB= 95:5, v:v%)


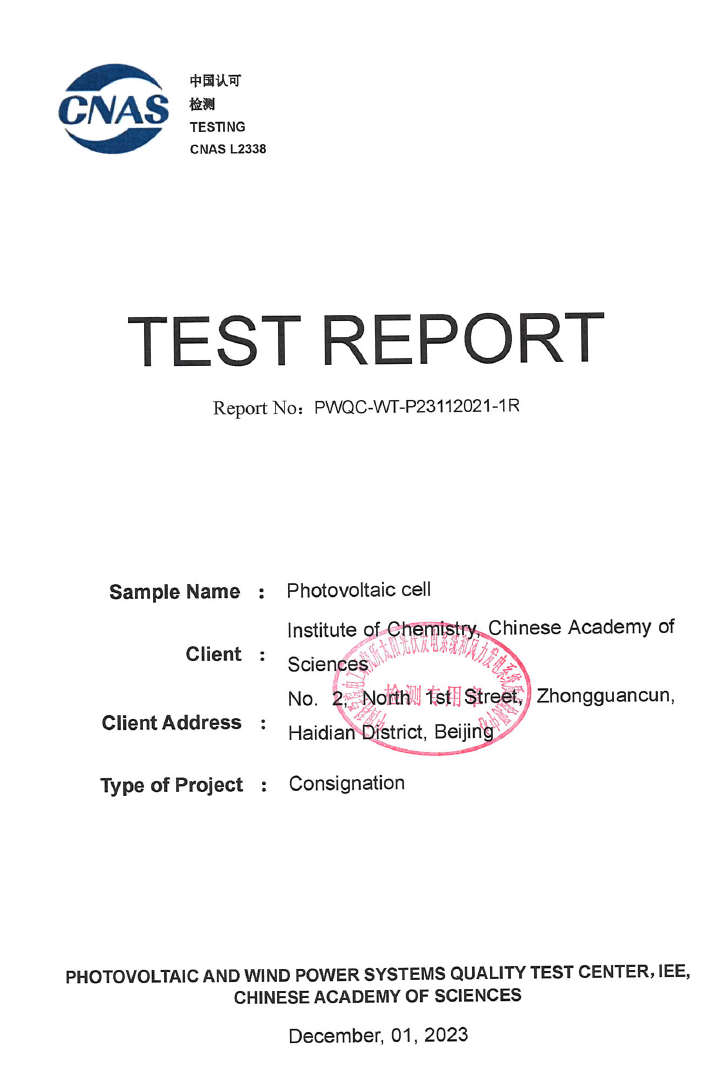

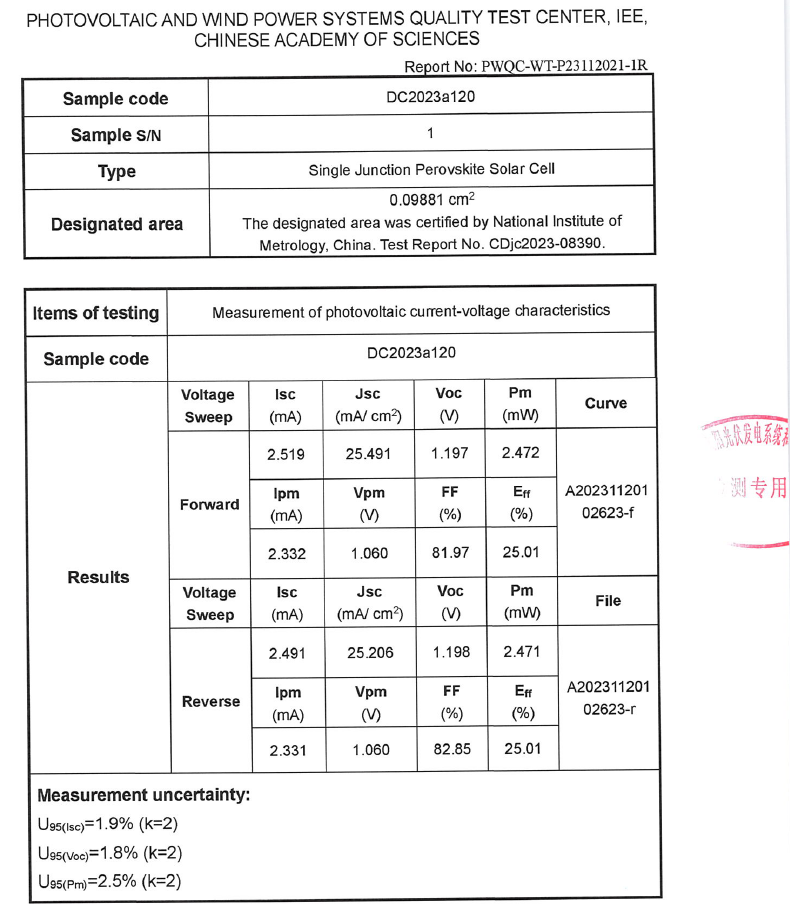


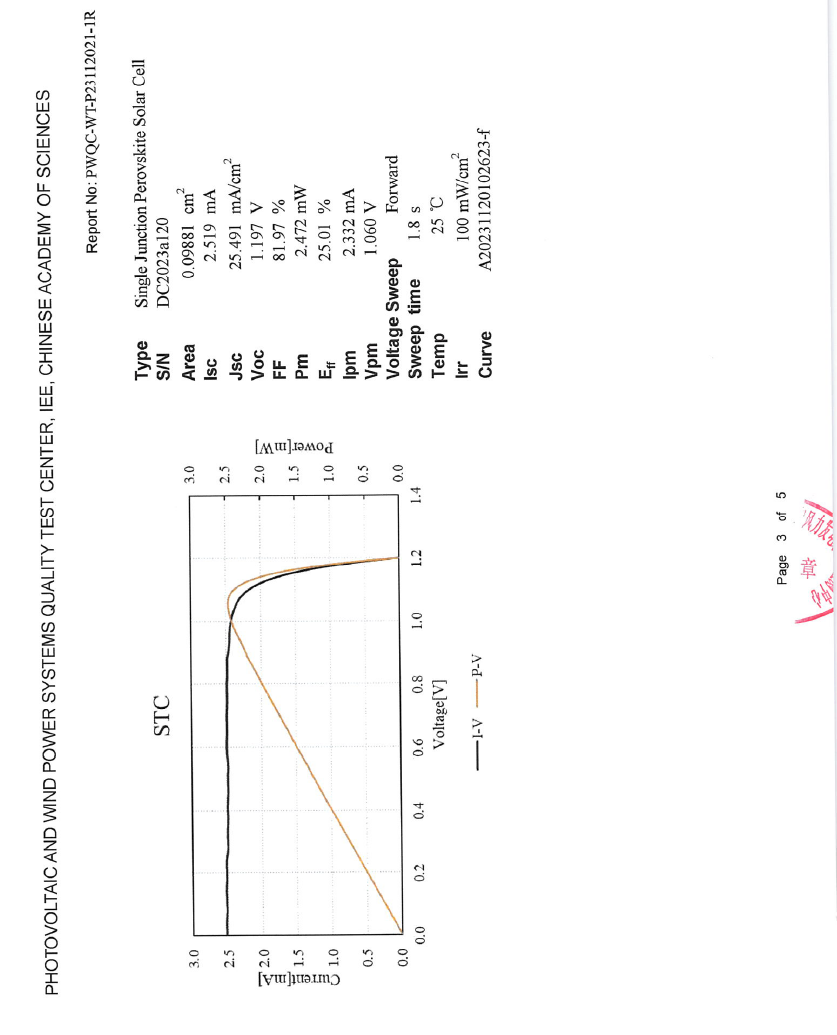


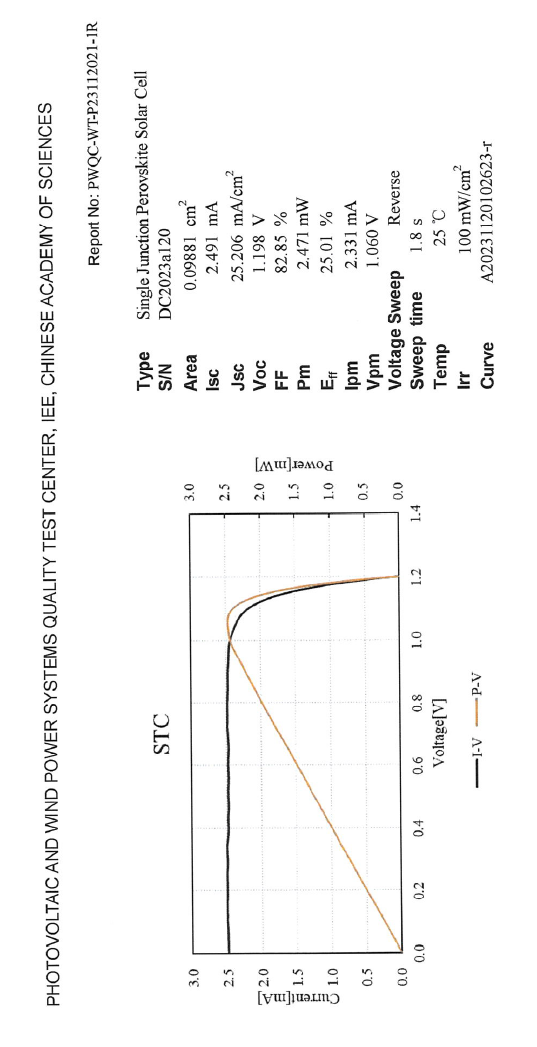


**Fig. S31** Certification of PSC from Institute of Electrical Engineering Chinese Academy of Sciences. The certified efficiency is 25.01%


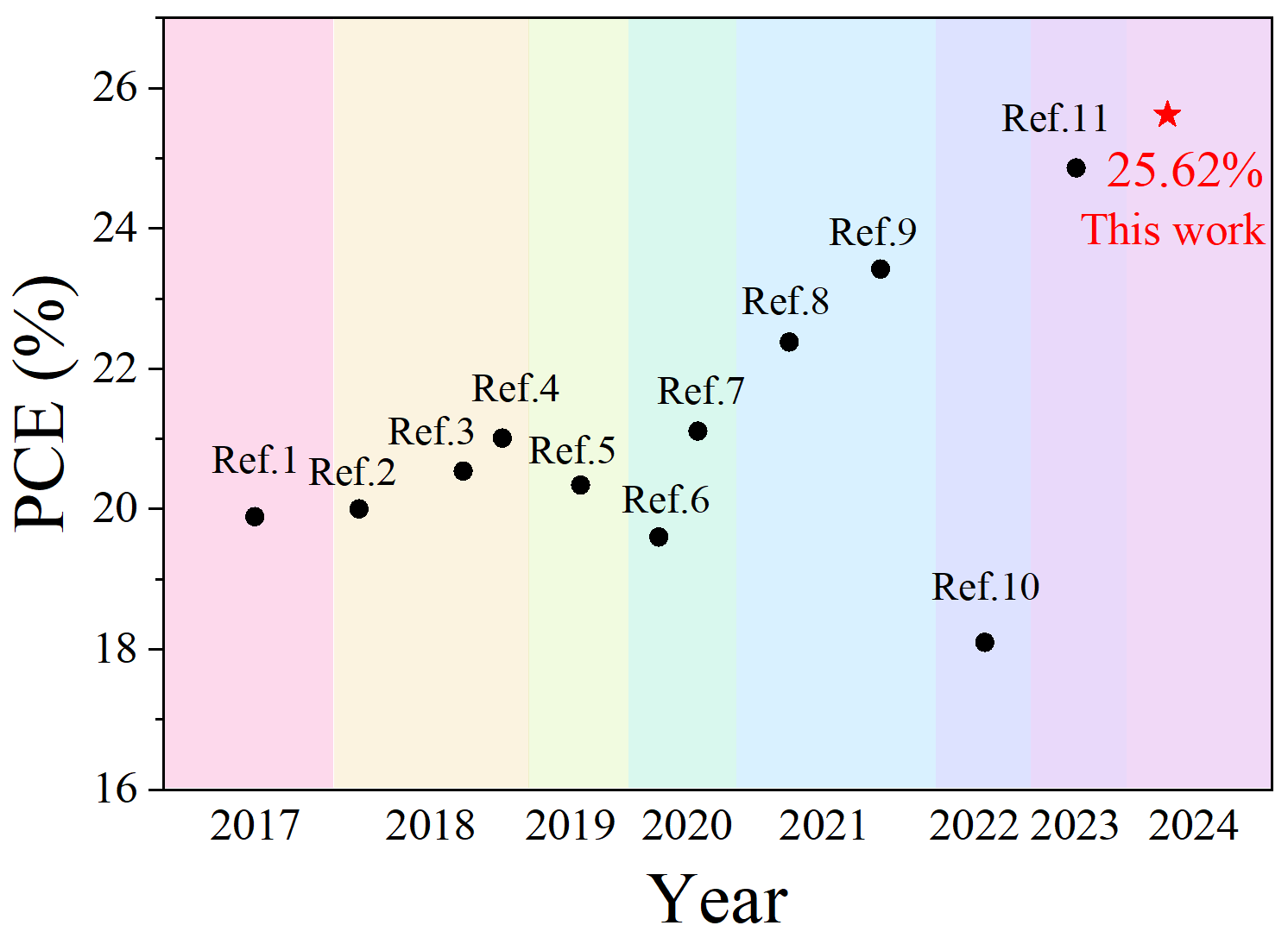


**Fig. S32** Photovoltaic efficiency of GDY-involved PSCs in recent years


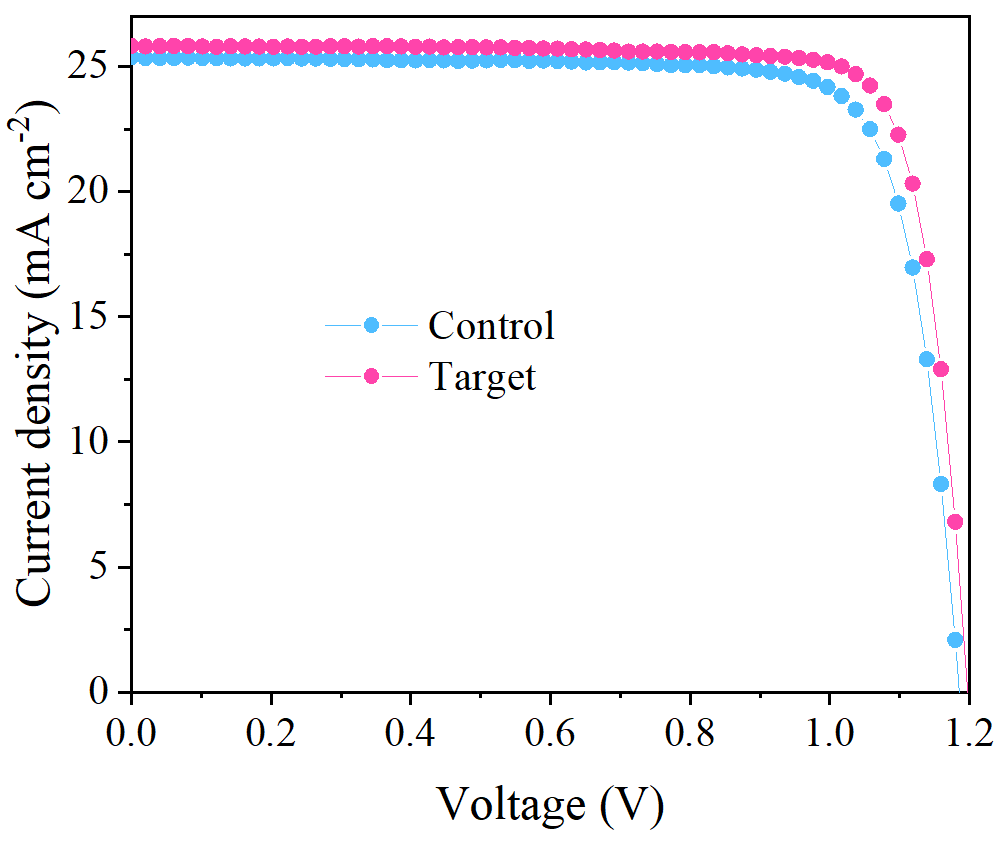


**Fig. S33** The J-V curves of the Cs_0.05_FA_0.95_PbI_3_-based PSCs in the reverse scan (RS) direction


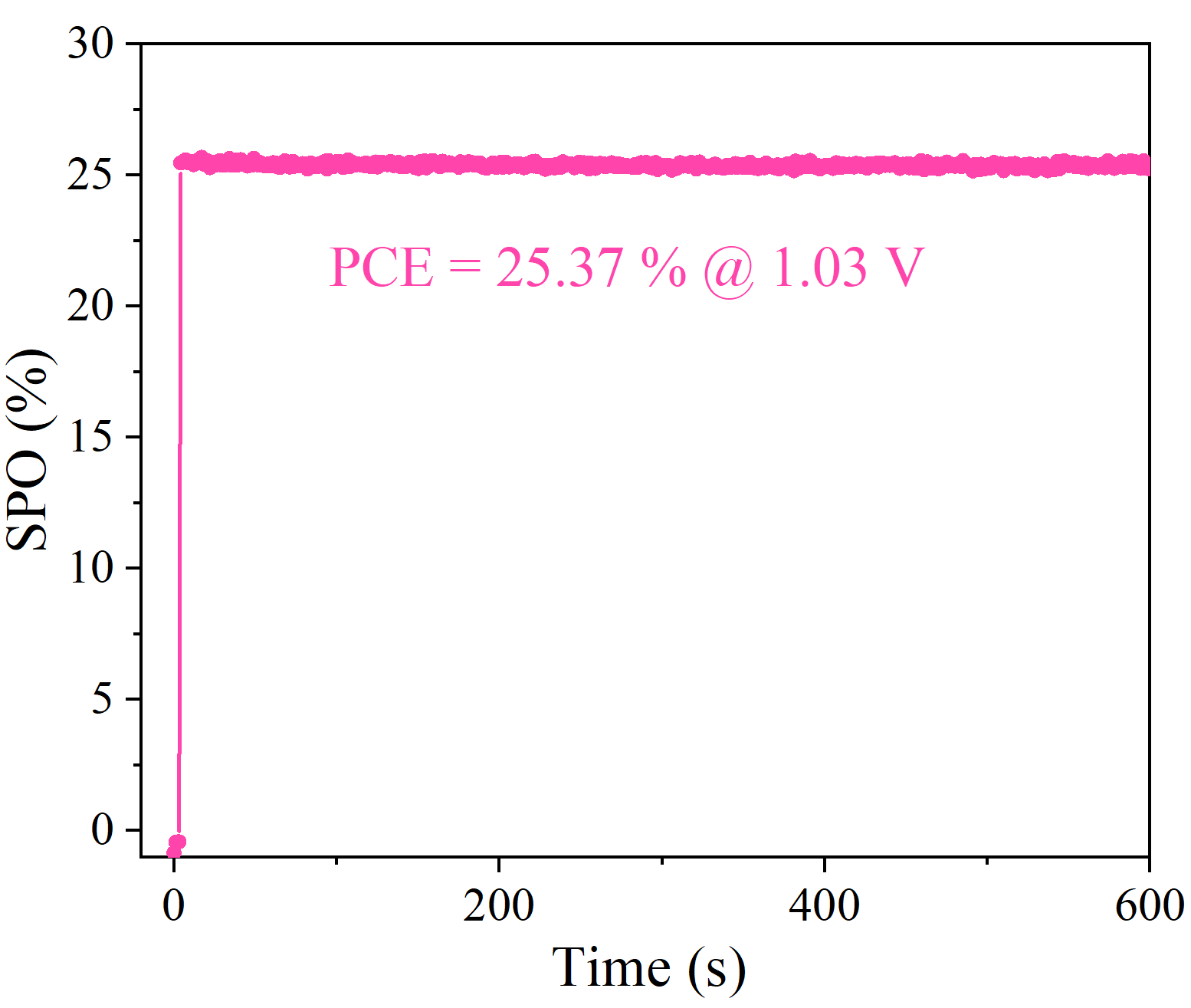


**Fig. S34** The stabilized power output of the target PSC


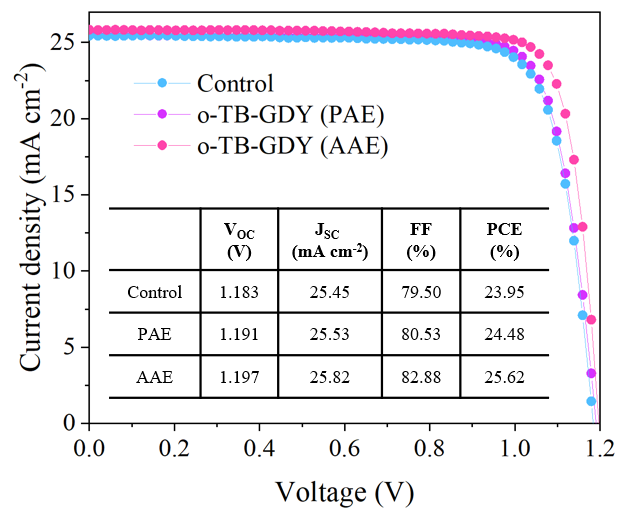


**Fig. S35** The J-V curves of PSCs without o-TB-GDY (control), with o-TB-GDY by perovskite precursor additive engineering (PAE) and with o-TB-GDY by antisolvent additive engineering (AAE) in the reverse scan (RS) direction. We found that the PAE can also moderately improve the performance of PSCs. However, the AAE can more effectively improve photovoltaic performance. This may be because that o-TB-GDY in the precursor can improve the perovskite crystallization, but the surface defects remain to be passivated. While, the AAE can simultaneously achieve surface passivation and better perovskite grain growth, the performance is greatly enhanced


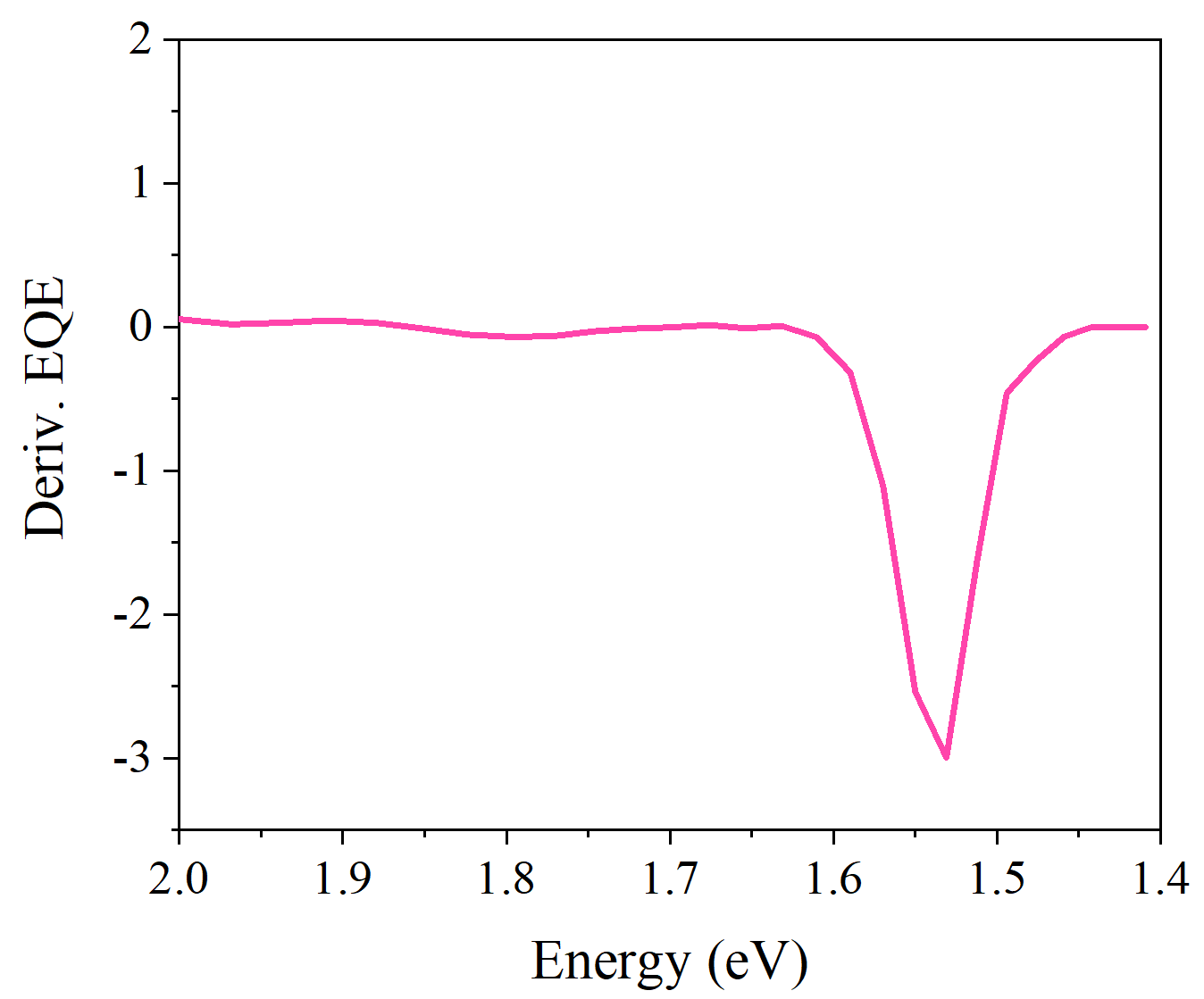


**Fig. S36** Analysis of perovskite bandgap from the derivative of the external quantum efficiency (EQE) spectrum. The bandgap is estimated to be ~ 1.53 eV


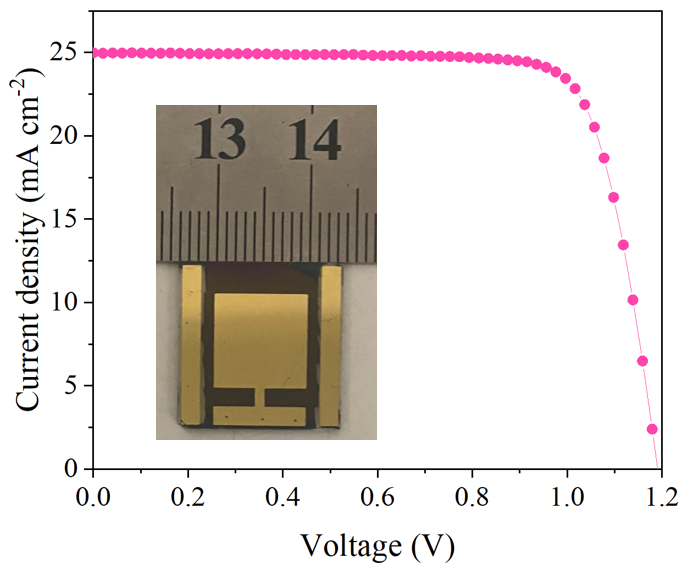


**Fig. S37** J–V curves of a target PSC with an aperture area of 1 cm^2^


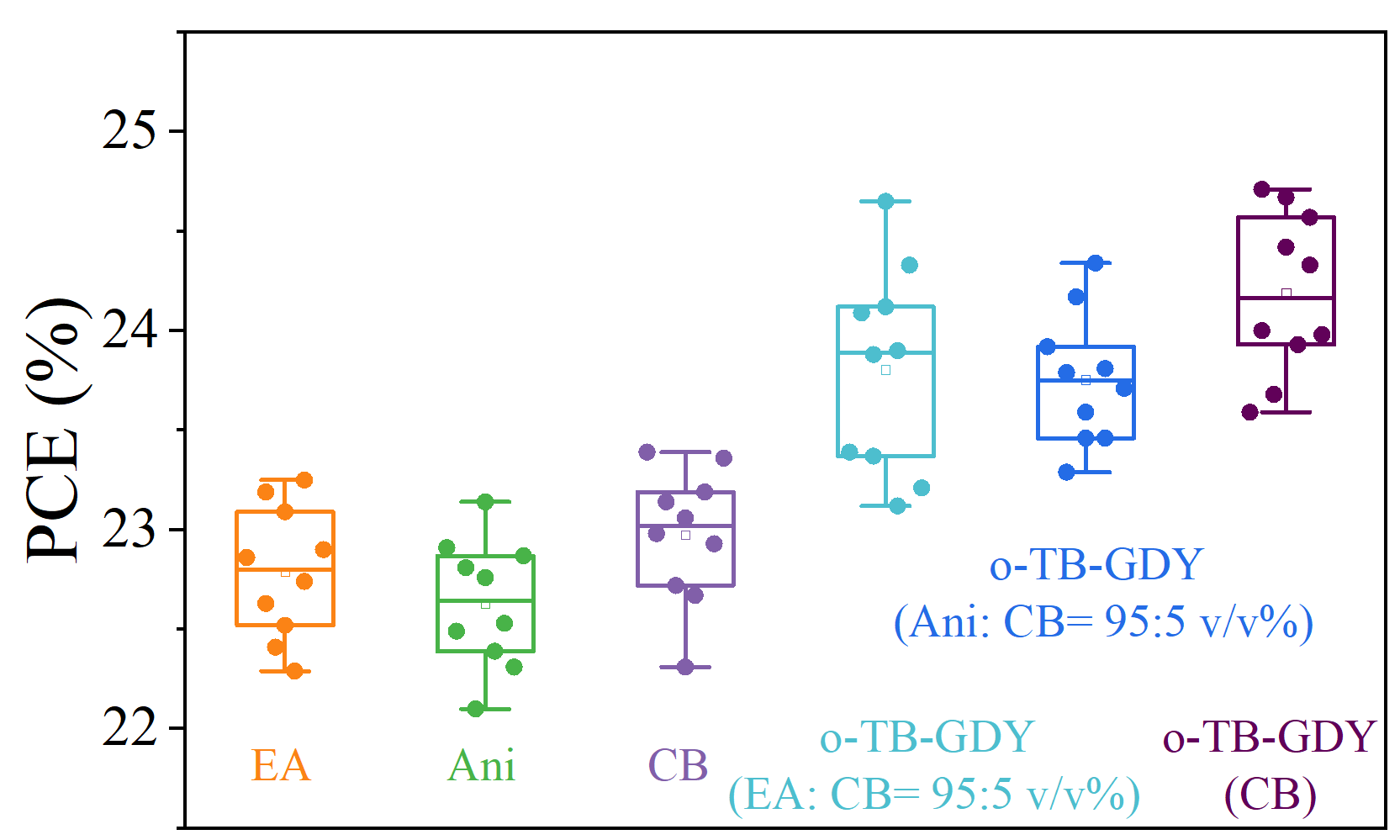


**Fig. S38** Statistical distributions of PCE for 10 PSCs based on different antisolvents


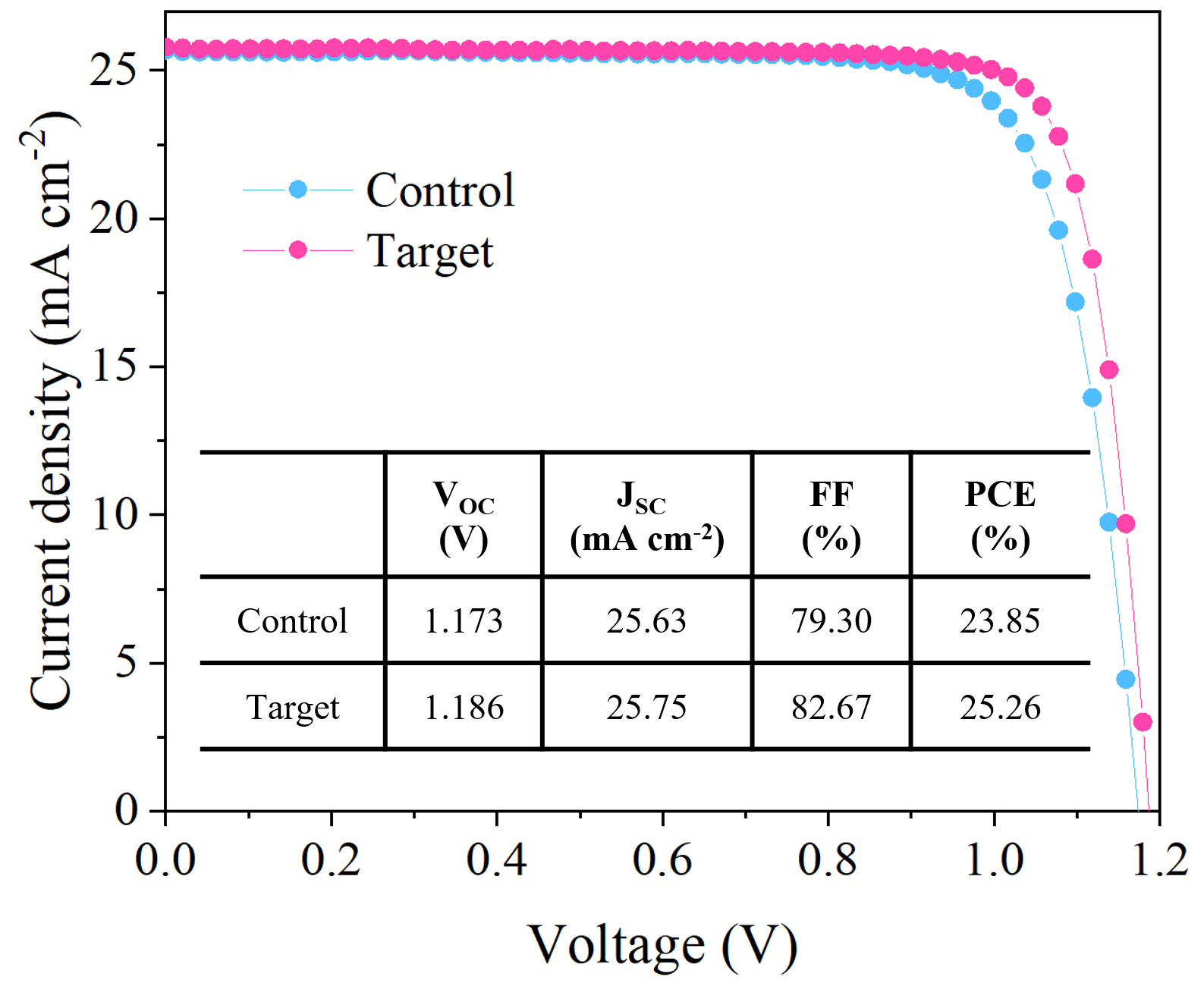


**Fig. S39** The J-V curves of the Cs-free (FAPbI_3_)_0.99_(MAPbBr_3_)_0.01_-based PSCs without (control) and with (target) o-TB-GDY treatment in the reverse scan (RS) direction


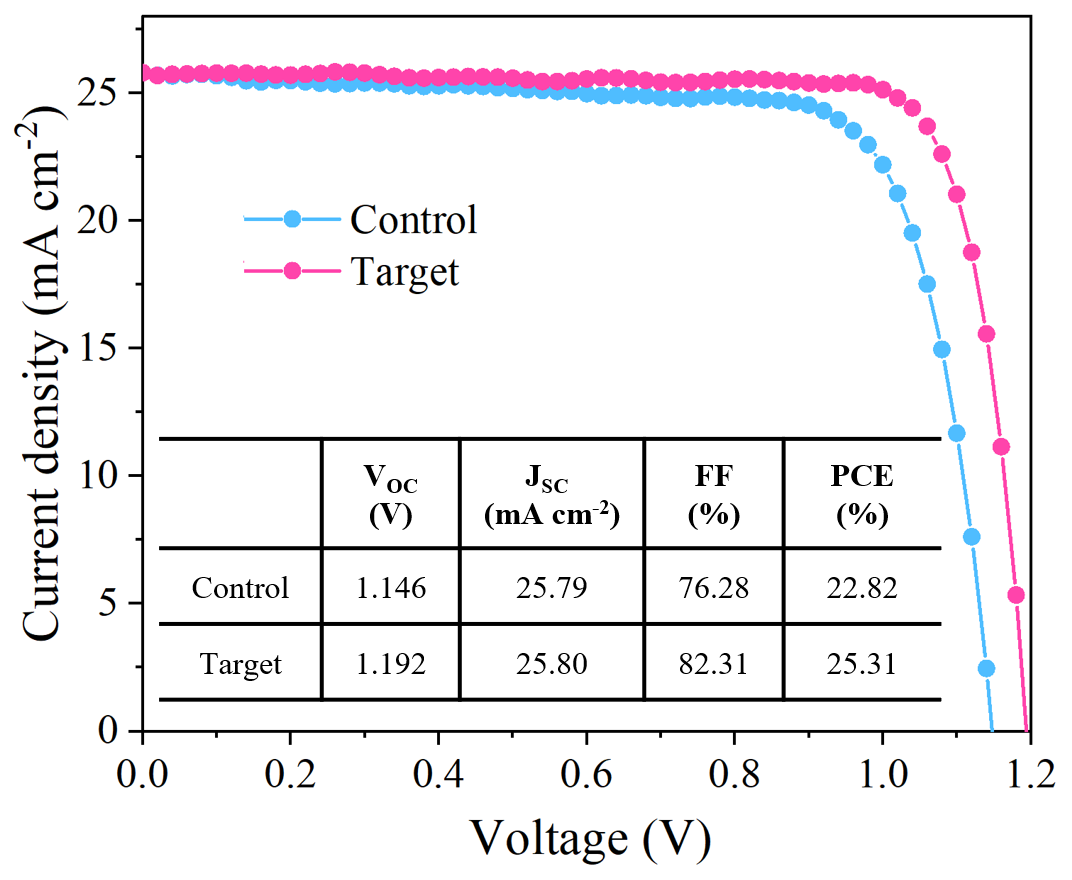


**Fig. S40** The J-V curves of the triple cation mixed-halide perovskite system, Cs_0.05_MA_0.05_FA_0.9_PbI_3_-based PSCs without (control) and with (target) o-TB-GDY treatment in the reverse scan (RS) direction


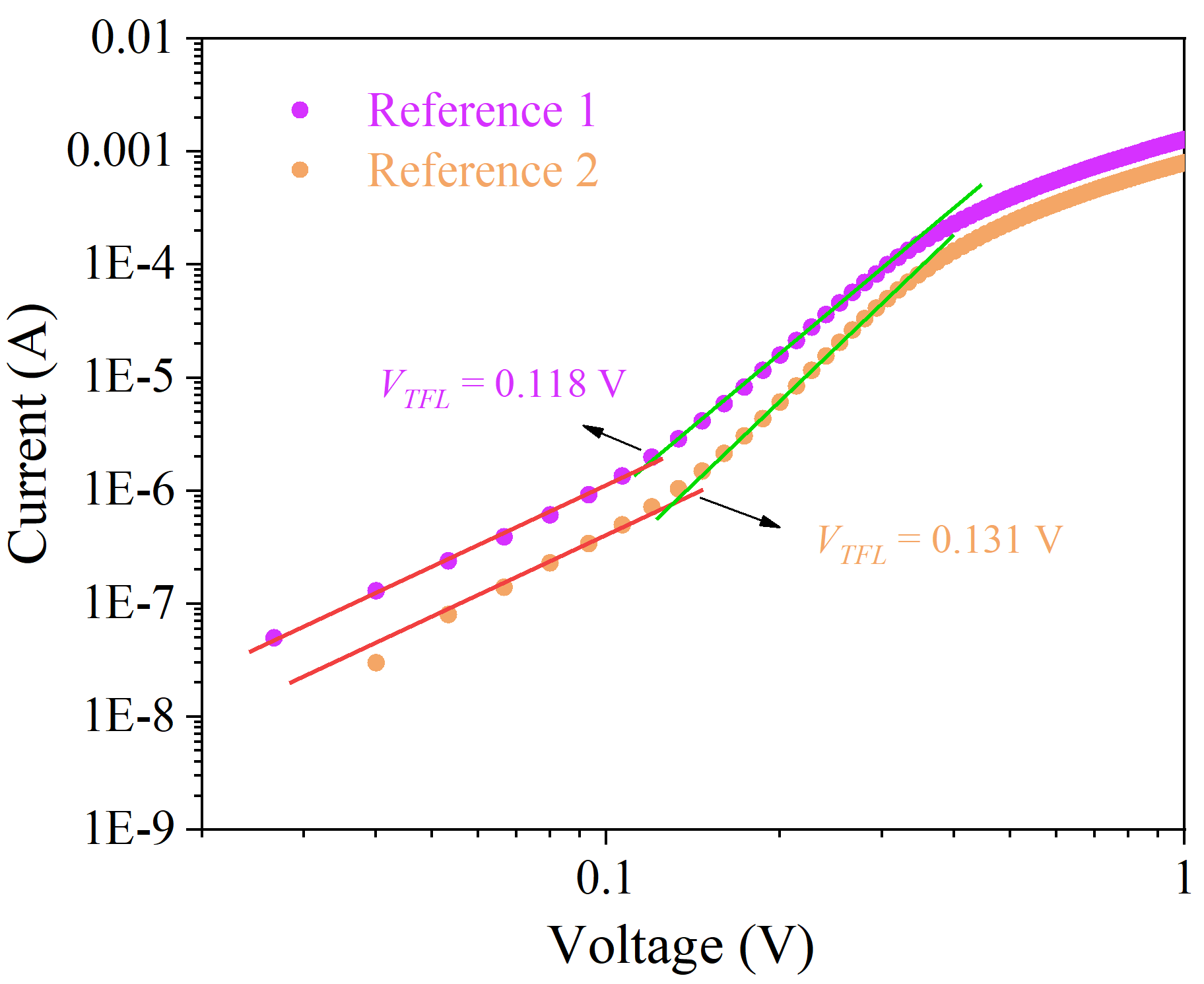


**Fig. S41** Space charge-limited current (SCLC) analysis for the reference 1 and reference 2 hole-only devices. V_TFL_ is trap filled limit voltage


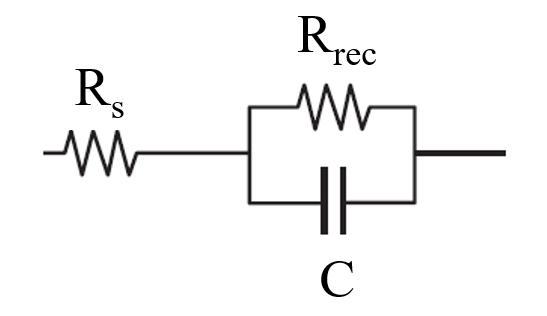


**Fig. S42** The equivalent-circuit model (ECM) employed for EIS fitting. It consists of the series resistance (Rs), the recombination resistance (Rrec) and the capacitance


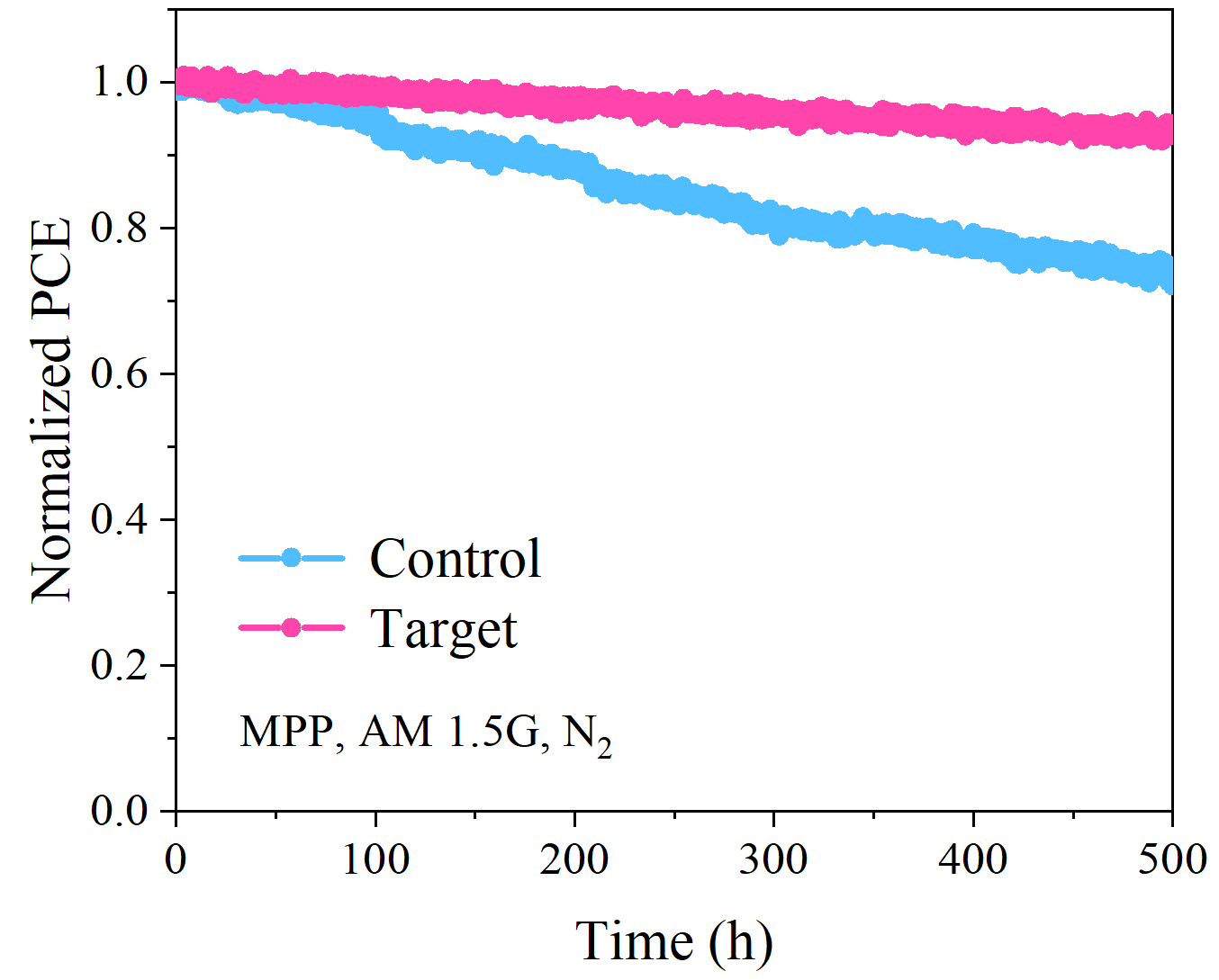


**Fig. S43** Continuous MPP tracking of the control and target PSCs


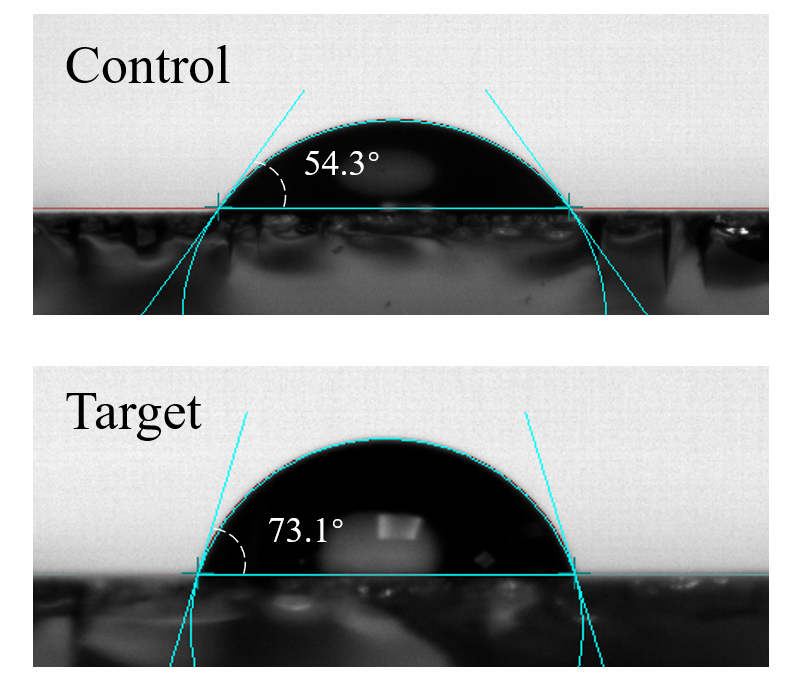


**Fig. S44** Contact angles of water on the control and target films


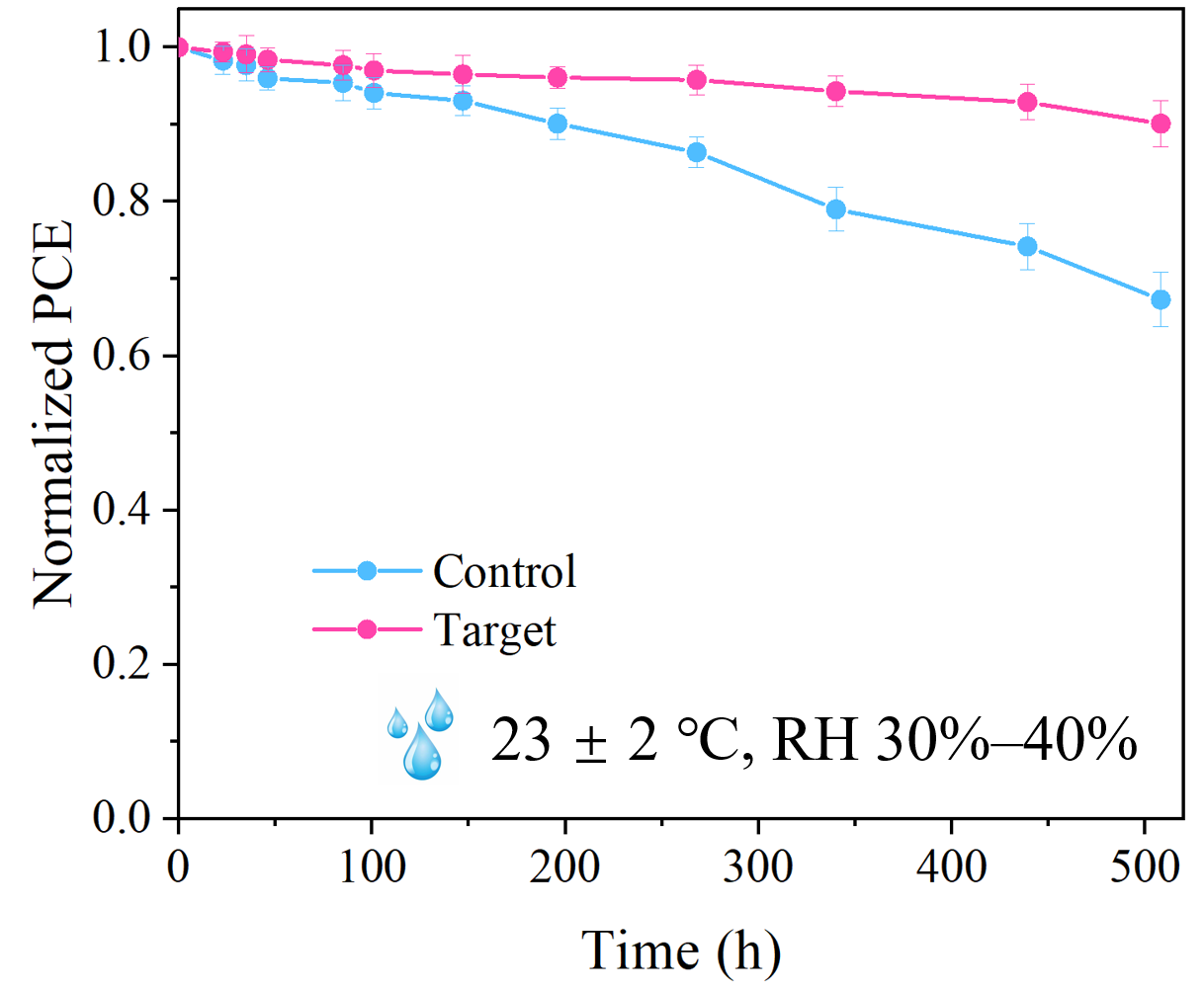


**Fig. S45** Stability of unencapsulated devices at 23 ± 2 °C and 30%-40% RH

**Table S1** DFT calculation results of o-TB-GDY/PbI_2_ bonding energies

|  | o-TB-GDY/PbI_2_ |
| --- | --- |
| Binding energy (eV) | 4.08 |

**Table S2** Fitting results from time-resolved PL measurements of the control, reference 1, reference 2, and target films. The TRPL spectra were fitted using the double exponential decay function: $Y=A_{1}exp(-t/\tau_{1})+A_{2}exp(-t/\tau_{2})$, where A_1_ and A_2_ represent the relative amplitudes, $\tau_{1}$ and $\tau_{2}$ denote the fast and slow decay time constants, relating to the radiative and trap-assisted non-radiative recombination processes, respectively. The average lifetime was calculated using the equation $\tau_{ave}=\frac{A_{1}\tau_{1}^{2}+A_{2}\tau_{2}^{2}}{A_{1}\tau_{1}+A_{2}\tau_{2}}$.

|  | **A_1_** | $\boldsymbol{\tau}_{\boldsymbol{1}}$ **(ns)** | **A_2_** | $\boldsymbol{\tau}_{\boldsymbol{2}}$ **(ns)** | $\boldsymbol{\tau}_{\boldsymbol{ave}}$ **(ns)** |
| --- | --- | --- | --- | --- | --- |
| **Target** | 26.7 | 200 | 972.2 | 11439.4 | 11434.0 |
| **Reference 1** | 74.2 | 2000 | 947.9 | 9806.4 | 9683.7 |
| **Reference 2** | 43.9 | 357.3 | 1021.4 | 7554.6 | 7540.0 |
| **Control** | 43.5 | 87.45 | 1067.1 | 7730.9 | 7727.4 |

**Table S3** Fitting results from time-resolved PL measurements of the perovskite films with different o-TB-GDY loadings

|  | **A_1_** | $\boldsymbol{\tau}_{\boldsymbol{1}}$ **(ns)** | **A_2_** | $\boldsymbol{\tau}_{\boldsymbol{2}}$**(ns)** | $\boldsymbol{\tau}_{\boldsymbol{ave}}$ **(ns)** |
| --- | --- | --- | --- | --- | --- |
| **Control** | 43.5 | 87.45 | 1067.1 | 7730.9 | 7727.4 |
| **0.01 mg ml^-1^** | 227.6 | 2317.6 | 753.5 | 9628.1 | 9132.6 |
| **0.03 mg ml^-1^ (Target)** | 26.7 | 200 | 972.2 | 11439.4 | 11434.0 |
| **0.05 mg ml^-1^** | 74.4 | 200 | 961.8 | 9082.9 | 9067.8 |

**Table S4** Fitting results from time-resolved PL measurements of different perovskite films with spiro-OMeTAD atop

|  | **A_1_** | $\boldsymbol{\tau}_{\boldsymbol{1}}$ **(ns)** | **A_2_** | $\boldsymbol{\tau}_{\boldsymbol{2}}$ **(ns)** | $\boldsymbol{\tau}_{\boldsymbol{ave}}$ **(ns)** |
| --- | --- | --- | --- | --- | --- |
| **Control** | 636.94 | 21.30 | 96.54 | 74.38 | 39.87 |
| **Reference 1** | 593.53 | 21.84 | 136.19 | 62.92 | 38.19 |
| **Reference 2** | 647.00 | 24.86 | 127.82 | 77.14 | 44.73 |
| **0.01 mg ml^-1^** | 728.48 | 27.15 | 76.84 | 116.32 | 54.90 |
| **0.03 mg ml^-1^ (Target)** | 534.22 | 23.46 | 175.05 | 60.73 | 40.56 |
| **0.05 mg ml^-1^** | 621.52 | 28.74 | 69.72 | 121.93 | 58.79 |

**Table S5** PLQY results of the control, reference 1, reference 2, and target films. Data are presented as mean values ± s.e.m for three samples

| Sample | PLQY (%) | |
| --- | --- | --- |
|  | Value | Mean values |
| **Control** | 1.23 | 1.19 ± 0.040 |
|  | 1.15 |  |
|  | 1.18 |  |
| **Reference 1** | 2.05 | 2.05 ± 0.025 |
|  | 2.03 |  |
|  | 2.08 |  |
| **Reference 2** | 1.17 | 1.18 ± 0.021 |
|  | 1.20 |  |
|  | 1.16 |  |
| **Target** | 3.12 | 3.17 ± 0.057 |
|  | 3.15 |  |
|  | 3.23 |  |

**Table S6** Fitting results of fs-TA spectra at 783 nm

|  | **A_1_ (%)** | **τ_1_ (ps)** | **A_2_ (%)** | **τ_2_ (ps)** | **τ_avg_ (ps)** |
| --- | --- | --- | --- | --- | --- |
| **Control** | 21.9 | 48.8 | 78.1 | 7490 | 7476.4 |
| **Target** | 16.9 | 32.5 | 83.1 | 9060 | 9053.4 |

**Table S7** Summary of photovoltaic parameters of the control and target PSCs in reverse scan (RS) and forward scan (FS)

|  | **Scan Directions** | **V_OC_**  **(V)** | **J_SC_**  **(mA cm^-2^)** | **FF**  **(%)** | **PCE**  **(%)** | **Hysteresis**  **(%)** |
| --- | --- | --- | --- | --- | --- | --- |
| **Control** | RS | 1.185 | 25.36 | 80.50 | 24.18 | 1.57% |
|  | FS | 1.182 | 25.54 | 78.80 | 23.80 |  |
| **Target** | RS | 1.197 | 25.82 | 82.88 | 25.62 | 1.56% |
|  | FS | 1.198 | 25.76 | 81.74 | 25.22 |  |

**Table S8** Parameters from SCLCs of the hole-only devices. Trap densities were calculated using the V_TFL_ from the SCLC measurements and equation N_traps_ = 2ε_0_ε_r_V_TFL_/(qL^2^), where the ε_0_ is the vacuum permittivity, ε_r_ (47) is the relative dielectric constant of FAPbI_3_, q is the elementary charge, and L (600 nm) is the thickness of the perovskite films

|  | **Control** | **Reference 1** | **Reference 2** | **Target** |
| --- | --- | --- | --- | --- |
| **V_TFL_ (V)** | 0.133 | 0.118 | 0.131 | 0.096 |
| **Trap**  **density (cm^-3^)** | 1.9183 × 10^15^ | 1.7019 × 10^15^ | 1.8894 × 10^15^ | 1.3846 × 10^15^ |

**Table S9** EIS fitting parameters of the control and target PSCs

|  | **Rs (Ω)** | **Rrec (Ω)** |
| --- | --- | --- |
| **Control** | 80.59 | 2057 |
| **Target** | 69.20 | 4495 |

**Table S10** Analysis of the dark $J\text{-}V$ curves of the control and target PSCs

|  | ***R_sh_* (MΩ cm^-2^)** | ***J_r_* (mA cm^-2^)** | ***J_d_* (mA cm^-2^)** |
| --- | --- | --- | --- |
| **Control** | 1.6 | 4.8 × 10^-7^ | 1.6 × 10^-10^ |
| **Target** | 5.2 | 1.3 × 10^-7^ | 6.6 × 10^-11^ |

**Table S11** PCE Summary of GDY-involved PSCs depicted in Fig. S30

| Device structure | PCE (%) | Year | Refs. |
| --- | --- | --- | --- |
| FTO/TiO_2_/MAPbI_3_/Spiro-OMeTAD/Au | 19.89 | 2017 | [S1]Adv. Mater. Interfaces 2018, 5, 1701117 |
| FTO/TiO_2_/ FA_0.85_MA_0.15_Pb(I_0.85_Br_0.15_)_3_ /  Spiro-OMeTAD/Au | 20.54 | 2018 | [S2] *Adv Energy Mater*, 2018, **8**, 1802012. |
| ITO/P3CT-K/MAPbI_3_/PCBM/Al | 21.01 | 2018 | [S3] *Nano Lett*, 2018, **18**, 6941-6947 |
| ITO/P3CT-K/MAPbI_3_/PCBM/ZnO/Al | 20.0 | 2018 | [S4] *Nano Energy*, 2018, **46**, 331-337 |
| ITO/P3CT-K/MAPbI_3_/PCBM/Al | 20.34 | 2019 | [S5] *Solar RRL*, 2019, **3**, 1900241 |
| ITO/SnO_2_/FA/MA perovskite/Spiro-OMeTAD/Au | 21.11 | 2020 | [S6] *Angew. Chem. Int. Ed*, 2020, **59**, 11573-11582 |
| FTO/TiO_2_/PCBM/MAPbI_3_/Spiro-OMeTAD/Au | 19.6 | 2020 | [S7] *Small*, 2020, **16**, e1907290 |
| FTO/TiO_2_/ Cs_0.06_FA_0.78_MA_0.16_Pb(I_0.85_Br_0.15_)_3_/ Spiro-OMeTAD/Au | 22.38 | 2021 | [S8] *Adv Funct Mater*, 2021, **31**, 2104633 |
| ITO/SnO_2_/(FAPbI_3_)_1-x_(MAPbBr_3-y_Cl_y_)_x_/  Spiro-OMeTAD/Au | 23.42 | 2021 | [S9] *EcoMat*, 2021, **3**, e12092. |
| ITO/PEDOT:PSS/MAPbI_3_/PCBM/Ag | 18.10 | 2022 | [S10] *Nano Research*, 2022, **15**, 573-580 |
| ITO/SnO_2_/ FA_0.85_MA_0.15_Pb(I_0.95_Br_0.05_)_3_/  Spiro-OMeTAD/Au | 24.86 | 2023 | [S11] *Angew. Chem. Int. Ed*, 2023, **62**, e202311865 |
| ITO/SnO_2_/Cs_0.05_FA_0.95_PbI_3_/Spiro-OMeTAD/Au | 25.62 | 2024 | This work |

**Supplementary References**

1. X. Zhang, Q. Wang, Z. Jin, Y. Chen, H. Liu et al., Graphdiyne quantum dots for much improved stability and efficiency of perovskite solar cells. Adv. Mater. Interfaces **5**, 1701117 (2018). <http://doi.org/10.1002/admi.201701117>
2. H. S. Li, R. Zhang, Y. S. Li, Y. M. Li, H. B. Liu et al., Graphdiyne-based bulk heterojunction for efficient and moisture-stable planar perovskite solar cells. Adv. Energy Mater. **8**, 1802012 (2018). <http://doi.org/10.1002/aenm.201802012>
3. J. Li, T. Jiu, S. Chen, L. Liu, Q. Yao et al., Graphdiyne as a host active material for perovskite solar cell application. Nano Lett. **18**, 6941-6947 (2018). <http://doi.org/10.1021/acs.nanolett.8b02863>
4. J. Li, T. Jiu, C. Duan, Y. Wang, H. Zhang et al., Improved electron transport in MAPbI_3_ perovskite solar cells based on dual doping graphdiyne. Nano Energy **46**, 331-337 (2018). <http://doi.org/10.1016/j.nanoen.2018.02.014>
5. J. Li, N. Wang, F. Bi, S. Chen, C. Zhao et al., Inverted MAPbI_3_ perovskite solar cells with graphdiyne derivative‐incorporated electron transport layers exceeding 20% efficiency. Solar RRL **3**, 1900241 (2019). <http://doi.org/10.1002/solr.201900241>
6. S. Zhang, H. Si, W. Fan, M. Shi, M. Li et al., Graphdiyne: Bridging SnO_2_ and perovskite in planar solar cells. Angew. Chem. Int. Ed. **59**, 11573-11582 (2020). <http://doi.org/10.1002/anie.202003502>
7. J. Zhang, J. Tian, J. Fan, J. Yu, W. Ho, Graphdiyne: A brilliant hole accumulator for stable and efficient planar perovskite solar cells. Small **16**, e1907290 (2020). <http://doi.org/10.1002/smll.201907290>
8. W. Q. Fan, S. C. Zhang, C. Z. Xu, H. N. Si, Z. Z. Xiong et al., Grain boundary perfection enabled by pyridinic nitrogen doped graphdiyne in hybrid perovskite. Adv. Funct. Mater. **31**, 2104633 (2021). <http://doi.org/10.1002/adfm.202104633>
9. Y. Luan, F. Wang, J. Zhuang, T. Lin, Y. Wei et al., Dual‐function interface engineering for efficient perovskite solar cells. EcoMat. **3**, e12092 (2021). <http://doi.org/10.1002/eom2.12092>
10. H. Huang, B. Liu, D. Wang, R. Cui, X. Guo et al., Different mechanisms of improving CH3NH3PbI3 perovskite solar cells brought by fluorinated or nitrogen doped graphdiyne. Nano Research **15**, 573-580 (2022). <http://doi.org/10.1007/s12274-021-3522-9>
11. J. He, G. Hu, Y. Jiang, S. Zeng, G. Niu et al., Dual-interface engineering in perovskite solar cells with 2d carbides. Angew. Chem. Int. Ed. **62**, e202311865 (2023). <http://doi.org/10.1002/anie.202311865>
